# USP33 alleviates FIS1-dependent mitochondrial fission and cardiac microvascular injury in diabetic cardiomyopathy via deubiquitinating and stabilizing ATG7

**DOI:** 10.1038/s41419-026-08930-8

**Published:** 2026-05-30

**Authors:** Yuqiong Chen, Xiangyu Sun, Xinyan Li, Bo Guan, Xiaopei Yan, Chao Huang, Nannan Zhang, Wenjun Mao, Yuan Tian, Chao Chen, Yao Lu, Su Li

**Affiliations:** 1 Department of Cardiology, The Affiliated Suzhou Hospital of Nanjing Medical University, Suzhou Municipal Hospital, Gusu School, Nanjing Medical University, Suzhou, China; 2 Department of Breast Surgery, Cancer Hospital of China Medical University, Liaoning Cancer Hospital and Institute, Shenyang, China; 3 Center for Precision Cancer Medicine & Translational Research, Tianjin Medical University Cancer Institute and Hospital, National Clinical Research Center for Cancer, Key Laboratory of Cancer Prevention and Therapy, Tianjin’s Clinical Research Center for Cancer, Tianjin, China; 4 Department of Geriatrics, The Affiliated Suzhou Hospital of Nanjing Medical University, Suzhou Municipal Hospital, Gusu School, Nanjing Medical University, Suzhou, China; 5 Department of Respiratory Medicine, The Affiliated Suzhou Hospital of Nanjing Medical University, Suzhou Municipal Hospital, Gusu School, Nanjing Medical University, Suzhou, China; 6 Ministry of Science and Technology, the Affiliated Suzhou Hospital of Nanjing Medical University, Suzhou Municipal Hospital, Suzhou, Jiangsu China; 7 Department of Cardiology, Jinshan Hospital, Fudan University, China, Shanghai, China; 8 XuZhou Clinical School of Xuzhou Medical University, Department of Cardiology, Xuzhou Central Hospital, XuZhou Institute of Cardiovascular Disease, Xuzhou, P.R. China; 9 Department of cardiology, Zhongshan Hospital, Fudan University, Shanghai Institute of Cardiovascular Diseases, Shanghai, China

**Keywords:** Ubiquitylation, Vascular diseases

## Abstract

Endothelial dysfunction plays a key role in the development of diabetic cardiomyopathy (DCM), but the underlying mechanisms of endothelial dysfunction remain to be elucidated. Recent studies have revealed that dysregulated mitochondrial dynamics contributes to the development of cardiac microvascular dysfunction. Fission-1 (FIS1), a key effector of mitochondrial fission, functions as an outer mitochondrial membrane adapter that recruits dynamin-related protein-1 (Drp1) from the cytosol to the outer mitochondrial membrane for activating mitochondrial fission. The present study screened a library targeting deubiquitinases, and identified the regulatory role of USP33 on FIS1-dependent mitochondrial fission. We found USP33 silencing elevated FIS1 protein expression and resulted in excessive mitochondrial fission in endothelial cells, which in turn impaired mitochondrial function and worsen endothelial and cardiovascular dysfunction in DCM. Mechanistically, USP33 interacted with FIS1 at the TPR2 domain and promoted FIS1 degradation via lysosomal degradation. Further studies revealed that USP33 stabilized autophagy-related 7 (ATG7) at protein level by blocking K63-linked ubiquitination of human ATG7 at K48 (mouse K44) site. This process led to lysosomal degradation of FIS1 via ATG7-mediated autophagy. In summary, our findings reveal that USP33 plays a critical role in endothelial dysfunction in DCM and demonstrate that ATG7-FIS1 pathway acts as one of the potential downstream mechanisms.

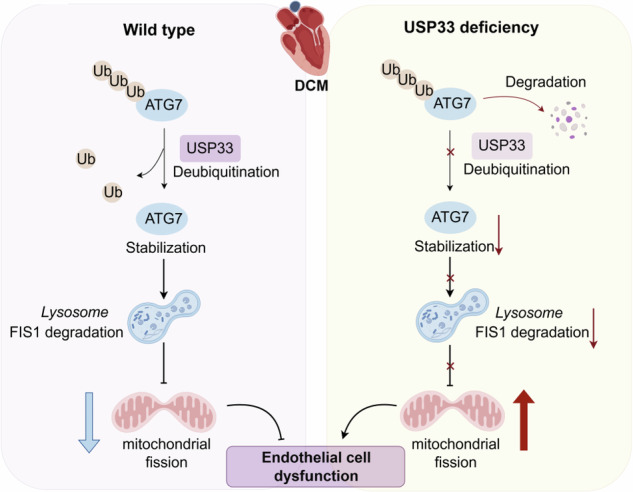

## Introduction

Microvascular dysfunction plays a crucial role in the development of diabetic cardiomyopathy (DCM) [1–4]. Cardiac microvascular endothelial cells (CMECs), the major components of cardiac microvasculature, are essential for maintaining myocardial perfusion and coronary reserves [5]. CMEC dysfunction has been identified as a key contributor to the microvascular injury in DCM, with diabetes mellitus (DM) and hyperglycemia as the primary contributors to CMEC dysfunction [1, 6, 7]. Thus, exploring the cellular and molecular mechanisms of cardiac microvascular dysfunction and identifying promising therapeutic targets are critical for improving the treatment of DCM.

Mitochondrial dynamics plays a critical role in shaping the mitochondrial network and regulating mitochondrial quality control [8]. Dysregulated mitochondrial dynamics have been implicated in the development of cardiac microvascular dysfunction [2, 6]. Altered mitochondrial dynamics is a contributing cause of endothelial dysfunction in the settings of DM [9]. Endothelial cells (ECs) collected from patients with DM exhibit loss of mitochondrial networks and increased mitochondrial fragmentation of mitochondria [9]. Notably, these alterations were accompanied by elevated levels of fission-1 (FIS1), an outer mitochondrial membrane (OMM) adapter that recruits dynamin-related protein-1 (Drp1) from the cytosol to the OMM for mitochondrial fission activation [10]. Even though limited evidence demonstrated FIS1 was upregulated and contributed to endothelial dysfunction in diabetes mellitus, the regulation of FIS1 in microvascular disturbance and DCM remains poorly understood [9]. Besides, endothelial FIS1 deSUMOylation exerts promotive effects on pulmonary endothelial function against hypoxic stress by maintaining endothelial mitochondrial integrity [11]. Therefore, post-translational modifications of FIS1 may significantly influence mitochondrial dynamics, although their precise mechanisms have yet to be fully elucidated.

Deubiquitinases (DUBs) are proteases that remove ubiquitin from substrates to regulate ubiquitylation and proteasomal degradation of proteins [12]. Dysregulation of DUBs has been implicated in various pathological processes, highlighting the importance of DUB function in cellular homeostasis [13]. USP33, a member of the USP family of DUBs, has been shown to deubiquitinate a wide range of proteins involved in diverse biological processes, including centrosome biogenesis, reticulophagy/autophagy, glycolysis, immune response and beta2 adrenergic receptor recycling [14–18]. However, whether USP33 plays a role in mitochondrial fission and FIS1 regulation remains unclear and deserves further investigations.

Autophagy is a protective degradation process that recycles cytoplasmic constituents through lysosomal degradation [19]. Accumulating evidence has demonstrated that autophagy plays an essential role in regulating EC function and maintaining endothelial homeostasis [20]. Endothelial autophagy has been shown to maintain homeostasis and function of cardiac endothelial cells [3, 20]. The improvement of endothelial autophagy alleviated cardiac microvascular dysfunction in DCM [3]. Autophagy related 7 (ATG7) is critical for canonical degradative macroautophagy/autophagy [21]. Inhibition of endothelial autophagy by EC-specific *Atg7* deletion results in inhibited angiogenesis in the ischemia diseases [22]. Besides, EC-specific *Atg7* deletion exacerbated doxorubicin-induced cardiotoxicity [23]. However, the role of ATG7-mediated autophagy in cardiac microvascular dysfunction in DCM remains to be fully elucidated.

Here, we screened a library targeting DUBs, and identified USP33 as the most powerful candidate for regulating the protein levels of FIS1. USP33 deficiency impaired microvascular function via the enhancement of FIS1-mediated mitochondrial fission. Mechanistically, USP33 blocked K63-linked ubiquitination of human ATG7 at K48, and therefore promoted the degradation of FIS1 via ATG7-mediated autophagy. In summary, our findings reveal that USP33-ATG7-FIS1 pathway plays a critical role in balancing endothelial mitochondrial dynamics during diabetes, which suggested USP33 may serve as a promising target for treating cardiac microvascular dysfunction in DCM.

## Methods

### Animals

All animal experiments were ethically approved by the Animal Experimental Ethics Committee and performed according to the animal experiment guidelines of Nanjing Medical University. Inducible endothelial-specific deletion of *USP33* (*USP33*^EC-KO^) mice were generated and purchased from M.Q. MICROBE Co., Ltd (Suzhou, China). *USP33*^flox/flox^ mice were crossed with mice carrying tamoxifen-inducible *Cdh5-*Cre^ERT2^ promoter. The homozygous were identified using polymerase chain reaction (PCR). 50 mg/kg tamoxifen dissolved in corn oil was injected intraperitoneally for 5 days to induce USP33 deletion. Mice were fed with high-fat diet (HFD) for 4 weeks, and then intraperitoneal injected 3 dose of streptozotocin (STZ; 40 mg/kg, Sigma, USA) to induce the type 2 diabetes mellitus (T2DM) model [7].

Adult male db/db mice and littermate nondiabetic male db/m mice were purchased from M.Q. MICROBE Co., Ltd (Suzhou, China). Four-week-old male db/m and db/db mice were transfected with Tie2-enhanced adeno-associated virus (AAV9) carrying mouse ATG7-wild-type (WT) or mouse ATG7-K44R mutant to achieve EC-specific overexpression of these proteins (QEgene, China). A total of 6 × 10^11^ vector genomes were injected via the tail vein every 8 weeks for 24 weeks [4]. The EC transfection efficiency and specificity of AAV9 were confirmed by western blotting and immunofluorescence.

### Cell culture and treatment

Human cardiac microvascular endothelial cells (HCMECs) were purchased from ScienCell Research Laboratories (USA). Human aortic endothelial cells (HAECs), human umbilical vein endothelial cells (HUVECs), and human coronary artery endothelial cells (HCAECs) were purchased from ZQXZBIO (Shanghai, China). The above endothelial cell lines were cultured in fibronectin-coated dishes with complete endothelial cell medium and used between passages 5 and 8. To induce in vitro diabetes model, cells were treated with 25 mM glucose and 0.5 mM free fatty acids (HG/FFA) injury for 72 h [2]. Cells in control were treated with 5.5 mM glucose, 19.5 mM mannitol, and 1% BSA.

CQ (10 μM) or Rapamycin (125–500 nM) were used to inhibit or stimulate autophagy, respectively. MA5 (5 μM) or AR7 (5 μM) were used to stimulate mitophagy and chaperone-mediated autophagy (CMA), respectively. FCCP (1 μM) or MFI8 (20 μM) were used to induce mitochondrial fission. SC9 (2 μM) was used to inhibit DRP1 and FIS1 interaction. CHX (100 μM) was adopted to block protein synthesis. MG132 (10 μM) was used to inhibit proteasome. Tenilsetam (5 μM) was used to suppress AGE. JSH-23 (10 μM) was used to inhibit NF-κB signaling. Go 6983 (100 nM) was used to reduced PKC pathway. Tempol (3 μM) was used to inhibit oxidative stress. ML385 (10 μM) was used to inhibit Nrf2 pathway.

### Cell transfection

Plasmids overexpressing 40 DUBs, USP33 wild type, USP33 mutants, FIS1 wild type, FIS1 mutants and ubiquitin (Ub) were designed and constructed by QEgene (Shanghai, China). HCMECs were transfected with plasmids using Lipofectamine 3000 reagents (Invitrogen™, USA) to overexpress target genes. Lentiviruses (LVs) encoding USP33, shUSP33, ATG7, and shATG7 were designed and constructed by QEgene (Shanghai, China). LVs encoding mRFP-GFP-LC3 (Hanbio Biotechnology, China) and adenoviruses (ADVs) encoding mt-Keima (Hanbio Biotechnology) were used to detect autophagy and mitophagy, respectively[3]. Cells were incubated with LVs at the recommended multiplicity of infection (MOI) for stable transfection.

### Reagents and antibodies

Chloroquine (CQ, S6999) and MG132 (S2619) were purchased from Selleck Chemicals (USA). FCCP (HY-100410), MFI8 (HY-150031), SC9 (HY-155656), cycloheximide (CHX, HY-12320), Rapamycin (HY-10219), Tenilsetam (HY-131528), JSH-23 (HY-13982), Go 6983 (HY-13689), Tempol (HY-100561) and ML385 (HY-100523 were purchased from MedChemExpress (USA).

Anti-USP33 antibody (20445-1-AP), anti-FIS1 antibody (66635-1-Ig), anti-Drp1 antibody (12957-1-AP), anti-ATG7 antibody (10088-2-AP), anti-Parkin antibody (14060-1-AP), anti-PINK1 antibody (23274-1-AP), anti-HA-tag (51064-2-AP), anti-MYC-tag (16286-1-AP) and anti-β-actin antibody (66009-1-Ig) were purchased from Proteintech (China). Anti-p-DRP1at Ser616 (4494S), anti-p-DRP1 at Ser637 (20990S), anti-p-IRS1 S612 antibody (2386), anti-IRS1 antibody (2382), anti-p-AKT1 473 antibody (4060) and anti-AKT1 antibody (2938) were purchased from Cell Signaling Technology (USA). Anti-FIS1 antibody (ab229969), anti-MFN2 antibody (ab124773), anti-LC3B antibody (ab192890), anti-P62 antibody (ab109012), anti-Tomm20 antibody (ab186735) and anti-FLAG-tag antibody (ab205606) were purchased from Abcam (USA). Anti-USP33 antibody (sc-100632) were purchased from Santa Cruz (USA).

### Echocardiography and sample collection

Two-dimensional echocardiography was performed to monitor cardiac function. The left ventricular ejection fraction (LVEF), left ventricular fractional shortening (LVFS), and E/A ratio were calculated. After echocardiography, the mice were euthanized and collected for serum and cardiac tissue. The information of heart weight and tibia length was collected to calculate the heart hypertrophy index.

### Histopathologic staining

Fresh cardiac tissues were fixed with 4% paraformaldehyde (PFA), dehydrated, embedded in paraffin and serially sectioned at a 5 μm thickness. Massons trichrome staining and wheat germ agglutinin (WGA) staining were performed to evaluate cardiac fibrosis and myocardial hypertrophy. The degree of cardiac fibrosis and myocardial hypertrophy was calculated using ImageJ software (version 1.53c, NIH, USA).

### RNA extraction and real-time PCR

The total RNA of cells was extracted by a Total RNA Extraction Kit (Solarbio), and reverse transcription was performed using a cDNA reverse transcription kit (Invitrogen, USA) according to the manufacturer’s instructions. The level of RNA was determined on the SYBR Green I (TSINGKE, China) on a Bio-Rad real-time PCR system. Gene expression was calculated by the 2 − ΔΔCt method.

### Assessment of mitochondrial function

Oxygen consumption rates (OCRs) were performed to assess the mitochondrial respiration activities using the Seahorse XF96 Extracellular Flux Analyzer (Seahorse Bioscience, USA). HCMECs and HCAECs were seeded at a concentration of 5×10^4^ cells/well and exposed to HG/FFA conditions for 72 h. After being starved for 6 h, oligomycin, FCCP, rotenone, and antimycin A were added to the plate following the instructions of the manufacturer before the OCRs were evaluated. Parameters including basal OCR, ATP-linked OCR, maximal OCR and spare respiratory OCR were recorded as previously reported[24].

### Western blotting and immunoprecipitation (IP) assay

Cells were prepared using RIPA lysis buffer (Beyotime, China) containing 1% protease inhibitor and then centrifuged at 12,000 rpm at 4 °C for 20 min. Protein samples were separated via SDS‒PAGE, and electrophoretically transferred to PVDF membranes (Millipore, USA). The membranes were then sealed with blocking buffer (Epizyme, China) for 20 min and incubated overnight with primary antibodies at 4 °C. Next, the membranes were incubated with conjugated secondary antibodies at room temperature for 1 h. The protein bands were visualized by electrochemiluminescence western blotting substrate (Thermo Fisher, USA), and the luminescence signals were measured with ImageJ software (version 1.53c, NIH, USA).

For co-immunoprecipitation (Co-IP) assay, the cells were lysed with Cell Lysis Buffer for IP (Beyotime, China) for protein extraction and incubated with Ig-A/G-magnetic beads (BioLinkedIn, China) that pre-conjugated with primary antibodies overnight. After washing, the samples were added with loading buffer and boiled at 100 °C for 10 min to prepare for western blot.

### Fluorescence staining of cells and tissues

For myocardial tissue microvascular perfusion, FITC-conjugated lectin (100 μL, 1 mg/mL) was injected intravenously into the mice through the tail vein to evaluate myocardial perfusion [24]. After 10 min, cardiac samples were harvested, sectioned for 5 μm slices and incubated with anti-CD31 antibody (ab7388, Abcam). The perfused microvascular density is calculated as the ratio of lectin-FITC-labeled microvessels to CD31-expressing microvessels.

For immunofluorescence staining of HCMECs, cells were seeded in confocal dishes, fixed with 4% PFA, permeabilized with 0.1% Triton X-100, and blocked with QuickBlock™ Blocking Buffer (Beyotime, China). The cells were incubated with primary antibodies against USP33, FIS1, ATG7, Parkin, Tomm20 and LC3B respectively at 4 °C overnight and fluorescence-labeled secondary antibodies for 1 h. Fluorescence images were captured by a laser confocal microscope (FV3000, Olympus, Japan) and qualified using ImageJ software (version 1.53c, NIH, USA).

Mitochondria were stained with 200 nM MitoTracker Red (Invitrogen, USA) according to the manufacturer’s instructions. Mitochondrial length was measured by Mitochondria Analyzer, a plug-in in ImageJ [25]. Quantification was based on a minimum of 30–50 cells in 5–8 images per sample.

For measurements of reactive oxygen species (ROS) and mitochondrial ROS (mitoROS), cells were plated in confocal dishes. HCMECs and HCAECs were seeded in a 96-well plate incubated with 10 μM DCFH-DA (Beyotime, China) and 1 μM MitoSOX (Invitrogen, USA) in fetal bovine serum (FBS)-free medium for 30 min at 37 °C in the dark according to the manufacturer’s instructions. Fluorescence images were captured by a laser confocal microscope (FV3000, Olympus, Japan) and qualified using ImageJ software (version 1.53c, NIH, USA). Mitochondrial membrane potential (MMP) was detected by TMRM staining. Cells were seeded in a 96-well black plate and incubated with 100 nM TMRM (Invitrogen, USA) for 30 min at 37 °C in the dark. The fluorescence intensities of TMRM were measured using a microplate reader (FlexStation 3, Molecular Devices) and adjusted according to the cell number.

### Measurements of cell viability and LDH release

Cell Counting Kit-8 (CCK-8, Epizyme, China) was used to detect cell viability following the manufacturer’s instructions. HCMECs were seeded in a 96-well plate with 10 μl CCK-8 reagent and 90 μl complete culture medium in each well. The plate was incubated for 4 h at 37 °C and the absorbance was monitored at 450 nm by a microplate reader (FlexStation 3, Molecular Devices). LDH Assay Kit (Beyotime) was used for detecting LDH release according to the manufacturer’s instructions, and the absorbance was monitored at 490 nm by a microplate reader (FlexStation 3, Molecular Devices).

### Transwell assays

For the Transwell assay, 5 × 10^5^ serum-free HCMECs were placed in the upper chambers of Transwell chambers (Corning, 8 µm well), and 500 μL complete culture medium containing 10% FBS was added to the lower chamber. After 24 h of culture, the migrated HCMECs were stained with crystal violet, and images were captured by an optical microscope (OLYMPUS CKX53, Japan) before calculated by ImageJ software (version 1.53c, NIH, USA).

### Detection of nitric oxide (NO) content

NO assay kit (Beyotime) was used to detect NO content in myocardial tissues and NO release from HCMECs. For measurement of NO content in myocardial tissues, the samples were homogenized and centrifuged (12,000 × g, 15 min) to collect the supernatant. The protein concentration was quantified using a Bradford protein assay kit (Solarbio, China). Then, the NO content in cardiac tissue was normalized to the protein concentration. For detecting NO release from HCMECs (5 × 10^5^ cells), the cell culture medium was directly assessed according to the manufacturer’s instructions, and the value was normalized to the cell number.

### Measurement of tissue and cellular monolayer permeability

The permeability of cardiac tissues was assessed by the Evans blue (EB) assay. EB dye (10 mg/ml) was injected intravenously via the tail vein for 30 min before sacrifice. The cardiac samples with the same weight were dissected and placed into 500 μL formamide solution, fully ground, incubated at 70 °C overnight. Then, the samples were centrifuged at 10,000 × g for 40 min to collect the supernatant. The absorbance of the supernatant and standards was measured using a microplate reader at 620 nm.

For assessing the cellular monolayer permeability in vitro, HCMECs were placed in the upper chambers of Transwell chambers (Corning, 0.4 µm well) for 3 days, and 100 μL FITC-dextran (1 mg/mL, Solarbio) was added to the upper chamber. The amount of FITC-dextran that penetrated to the lower chamber was measured by the fluorescence intensity of FITC-dextran. Transendothelial electrical resistance (TEER) was used to assess junctional function. HCMECs were seeded onto fibronectin-coated inserts. After the cells reached confluence and were subjected to the above treatments, a MilliCell ERS-2 system (Millipore, USA) was used to assess the TEER.

### Insulin-mediated vasorelaxation

A wire myograph system (Danish Myo Technologies, Denmark) was employed to record the isometric tension in the distal segments of the thoracic aorta [26, 27]. Experiments were conducted in 37 °C Krebs buffer that constantly gassed with 95% O2/5% CO2. Upon mounting, vessels were adjusted to a 2 g baseline. Viability was confirmed by a ≥ 70% contractile response to 60 mM KCl. After achieving 70% preconstriction of the resting diameter via norepinephrine (5 × 10^−8 ^M), cumulative dose-response curves for insulin (0.01–100 ng/ml) were generated, with results calculated as a percentage change of the initial baseline.

### Statistical analyses

All data presented in the study were expressed as the mean ± SEM. Differences between groups were analyzed by Student t test, one-way ANOVA followed by Tukey test, or two-way ANOVA with Tukey test. All analyses were performed by the GraphPad Prism software (version 8.0.1; San Diego, CA). Statistical significance was set as *P*-values less than 0.05.

## Results

### USP33 silencing promoted FIS1 upregulation and mitochondrial fission

**Fig. 1:**
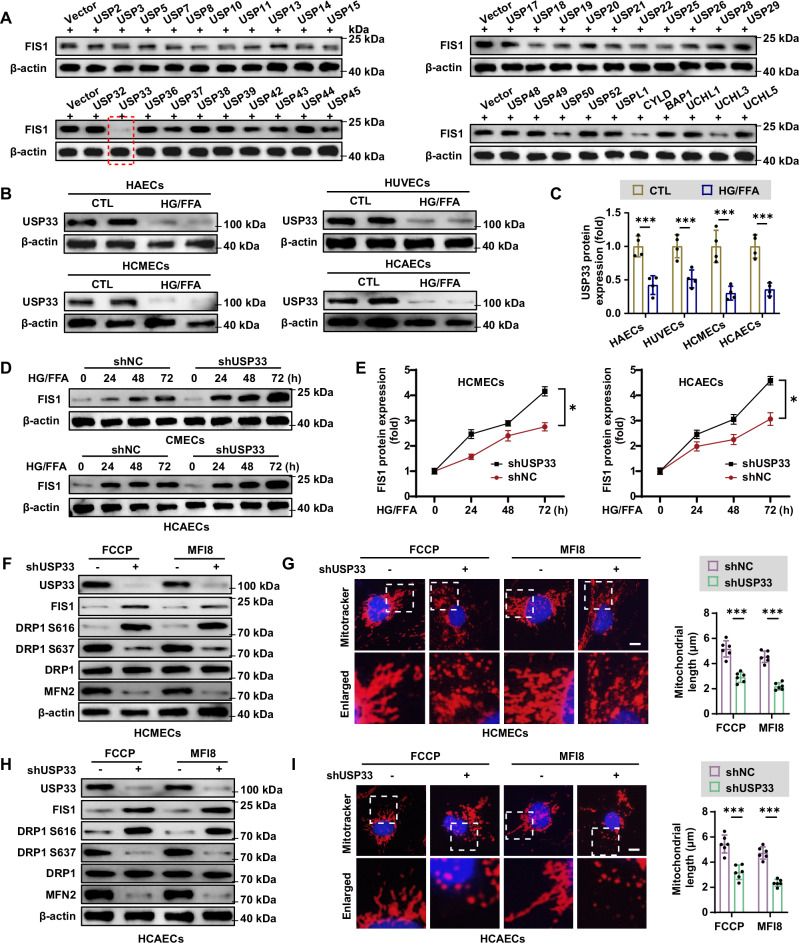
USP33 silencing promoted FIS1 upregulation and mitochondrial fission. **A** HCMECs were transfected with plasmids expressing DUBs for 48 h, and the expression of FIS1 was analyzed by western blot to screen potent DUBs that regulate FIS1 expression. **B**, **C** HAECs, HUVECs, HCMECs and HCAECs were subjected to HG/FFA injury for 72 h, the protein expression of USP33 were analyzed by western blot. **D**, **E** HCMECs and HCAECs were transfected with lentivirus expressing shUSP33 (LV-shUSP33) for 48 h, followed by HG/FFA exposure for the indicated durations. FIS1 protein levels were assessed by western blot. **F** HCMECs were transfected with LV-shUSP33 for 48 h and exposed to FCCP (1 μM, 6 h) or MFI8 (20 μM, 6 h) treatment, the expression of FIS1, USP33, DRP1 and MFN2, as well as the phosphorylation of DRP1 at Ser 616 and Ser 637 were analyzed by western blot. **G** Mitochondrial morphology in HCMECs was visualized by MitoTracker (red) staining, and mitochondrial length was quantified. Scale bar = 5 μm. **H** HCAECs were transfected with LV-shUSP33 for 48 h and exposed to FCCP (1 μM, 6 h) or MFI8 (20 μM, 6 h) treatment. The expression of FIS1, USP33, DRP1 and MFN2, as well asl the phosphorylation of DRP1 at Ser 616 and Ser 637 were analyzed by western blot. **I** Mitochondrial morphology in HCAECs was visualized by MitoTracker (red) staining, and mitochondrial length was quantified. Scale bar = 5 μm. *p < 0.05, **p < 0.01, ***p < 0.001 indicate significant differences. Four to six biological replicates were performed, and the results are indicated in scatter plots.

Whether DUBs could regulate FIS1 expression has not been reported. Herein, we transfected HCMECs with plasmids overexpressing DUBs and screened the DUB(s) responsible for FIS1 degradation. Among the 40 DUBs, USP33 was one of the most powerful candidates for downregulating the protein level of FIS1 (Fig. 1A). We then conducted a re-analysis of our previous RNA-Seq datasets from db/db mice and HG/FFA-treated HCMECs [1, 3], and found USP33 was suppressed at the transcriptional level (Fig. S1A, B). AGE-RAGE axis, NF-κB signaling, the PKC pathway, and oxidative stress/Nrf2 signaling were acknowledged canonical pathways implicated in hyperglycemia-induced gene regulation. We found the blockade of oxidative stress increased USP33 gene expression, and the inhibition of Nrf2 further decreased USP33 gene expression in HCMECs (Fig. S1C). Importantly, both the protein and mRNA levels of USP33 were significantly decreased after 72 h HG/FFA injury in multiple endothelial cell lines (Fig. 1B, C, and Fig. S1D).

Then, short hairpin RNA (shRNA) targeting USP33 were transfected to HCMECs and HCAECs to silence USP33 (Fig. S1E). Silencing USP33 did not affect FIS1 mRNA levels under HG/FFA conditions but significantly increased FIS1 protein levels in a time-dependent manner, suggesting that USP33 regulates FIS1 expression post-transcriptionally (Fig. 1D, E, and Fig. S1F, G). Next, we assessed whether USP33 regulated mitochondrial fission. Previous studies found that treatment of cells with mitochondrial uncouplers such as FCCP and MFI8 leads to activation of mitochondrial fission [28, 29]. We found USP33 silencing increased the expression of FIS1 and the phosphorylation of Drp1 at serine 616 (Ser616), whereas inhibited the expression of MFN2 and the phosphorylation of Drp1 at Ser637 in HCMECs after FCCP or MFI8 treatment (Fig. 1F, and Fig. S1H). USP33 silencing also promoted mitochondrial network disruption and mitochondrial fragmentation in HCMECs after FCCP or MFI8 treatment (Fig. 1G). Similar results were observed in HCAECs (Fig. 1H, I, and Fig. S1I). Collectively, these results suggested that USP33 regulates mitochondrial dynamics in endothelial cells, potentially by suppressing FIS1 protein expression.

### USP33 silencing aggravated mitochondrial dysfunction under HG/FFA conditions

**Fig. 2:**
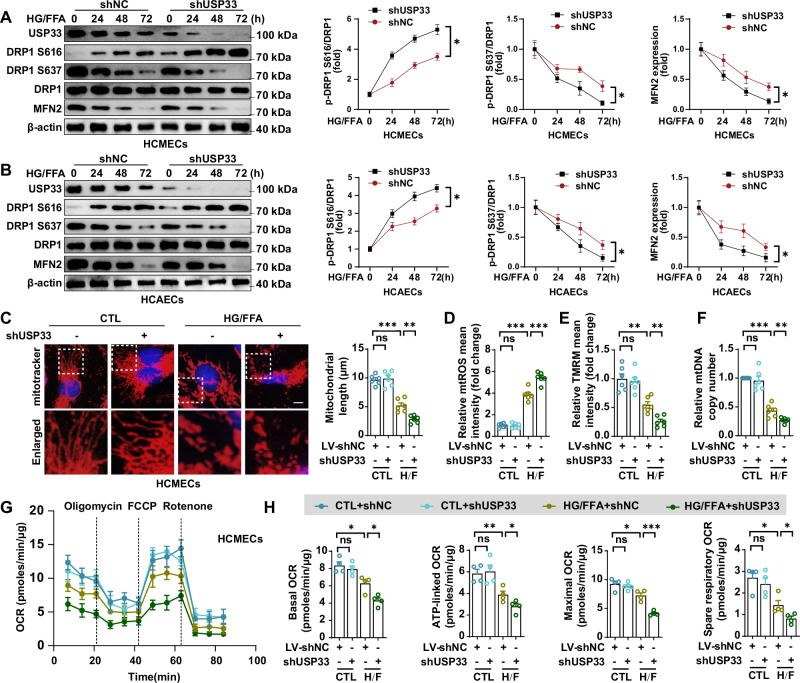
USP33 silencing aggravated mitochondrial dysfunction under HG/FFA conditions. HCMECs and HCAECs were transfected with LV-shUSP33 for 48 h and exposed to HG/FFA injury for 72 h. **A**, **B** The expression of USP33, DRP1 and MFN2, as well as the phosphorylation of DRP1 at Ser 616 and Ser 637 were analyzed by western blot in HCMECs and HCAECs. **C** The mitochondrial morphology of HCMECs was visualized by MitoTracker Red staining, and mitochondrial length was quantified. Scale bar = 5 μm. **D**–**F** Statistic analysis of mtROS level, mitochondrial membrane potential and mtDNA copy number were detected in HCMECs. **G**, **H** The mitochondrial respiratory activity of HCMECs was assessed using a Seahorse analyzer. The baseline OCR, ATP-linked OCR, maximal OCR and spare respiratory OCR were quantitatively analyzed. *p < 0.05, **p < 0.01, ***p < 0.001 indicate significant differences. Four to six biological replicates were performed, and the results are indicated in scatter plots.

To further investigate the role of USP33 in mitochondrial fission under diabetic conditions, HCMECs and HCAECs were exposed to HG/FFA injury. USP33 silencing in HCMECs and HCAECs further enhanced the phosphorylation of Drp1 at Ser616 but suppressed both MFN2 expression and Drp1 phosphorylation at Ser637 in a time-dependent manner under HG/FFA conditions (Fig. 2A, B). Furthermore, USP33 silencing markedly increased mitochondrial fragmentation and disrupted the mitochondrial network in HCMECs (Fig. 2C). HG/FFA injury given rise to mitochondrial oxidative stress and mitochondrial dysfunction as evidenced by increased mtROS accumulation, reduced mitochondrial membrane potential and decreased mitochondrial DNA copy number (Fig. 2D–F). However, these effects were further augmented by USP33 silencing under HG/FFA conditions (Fig. 2D–F). Consistent findings were observed in HCAECs (Fig. S2A–D). Mitochondrial respiration is the major parameter of mitochondrial function that normally reflected by basal OCR, ATP-linked OCR, maximal OCR, and spare respiratory OCR [30]. HG/FFA injury led to reduced level of basal OCR, ATP-linked OCR, maximal OCR, and spare respiratory OCR in HCMECs, the effects of which were further exacerbated by USP33 silencing (Fig. 2G, H). Consistent findings were demonstrated in HCAECs (Fig. S2E, F). Collectively, these results suggest that USP33 silencing promotes endothelial mitochondrial fission and exacerbates mitochondrial dysfunction under HG/FFA conditions.

### USP33 silencing exacerbated endothelial dysfunction under HG/FFA injury in a FIS1-dependent manner

**Fig. 3:**
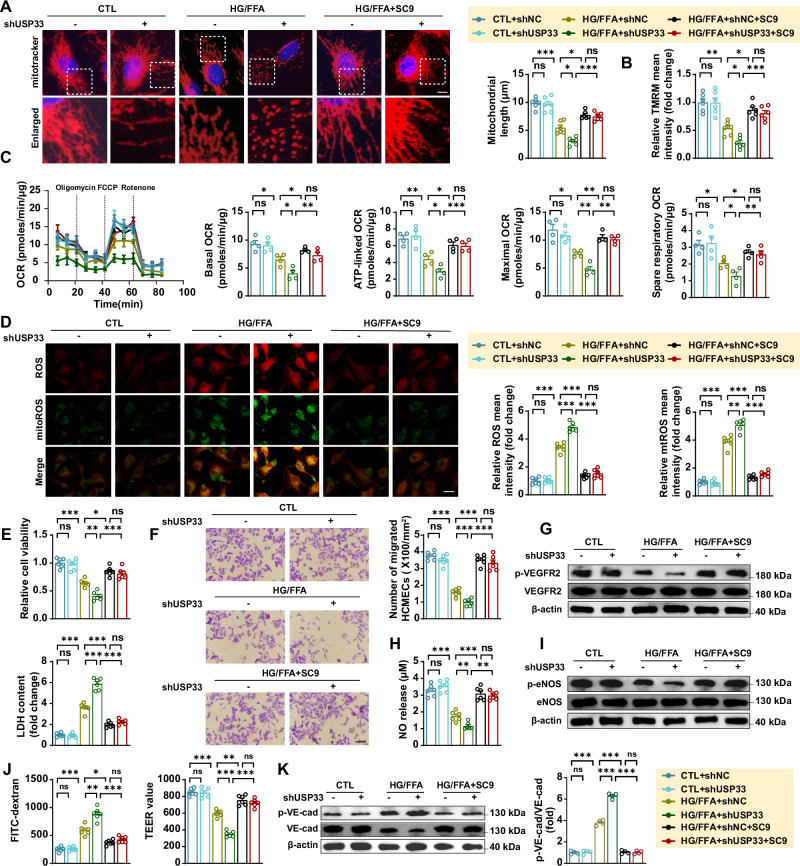
USP33 silencing exacerbated endothelial dysfunction under HG/FFA injury in a FIS1-dependent manner. HCMECs were transfected with LV-shUSP33 for 48 h, with or without SC9 treatment (2 μM) for 72 h. **A** The mitochondrial morphology of HCMECs was visualized by MitoTracker Red staining, and mitochondrial length was quantified. Scale bar = 5 μm. **B** The mitochondrial membrane potential of HCMECs was detected by TMRM staining. **C** The mitochondrial respiratory activity of HCMECs was assessed using a Seahorse analyzer. The baseline OCR, ATP-linked OCR, maximal OCR and spare respiratory OCR were quantitatively analyzed. **D** Intracellular ROS (green) and mitoROS (red) were photographed and quantitatively analyzed. Scale bar = 20 μm. **E** Relative cell viability and LDH release were statistically analyzed in the indicated groups. **F** Representative images of the transwell assay and statistical analysis of migrated cells. Scale bars: 100 μm. **G** Total and phosphorylated VEGFR2 protein expression was detected by western blot. **H** Statistical analysis of NO release in the indicated groups. **I** Total and phosphorylated eNOS protein expression was detected by western blot. **J** Statistical analysis of FITC-dextran permeability and TEER value in the indicated groups. **K** Total and phosphorylated protein expression of VE-cadherin was detected by western blot. *p < 0.05, **p < 0.01, ***p < 0.001 indicate significant differences. Four to six biological replicates were performed, and the results are indicated in scatter plots.

To determine whether USP33 silencing exacerbates endothelial dysfunction via FIS1-dependent mitochondrial fission, we utilized SC9, a known inhibitor for Drp1-FIS1 interaction, to block FIS1-mediated fission[31]. USP33 silencing promoted mitochondrial fragmentation in HCMECs under HG/FFA conditions, whereas SC9 significantly improved the mitochondrial network (Fig. 3A). USP33 silencing also accentuated mitochondrial dysfunction under HG/FFA conditions as reflected by reduced TMRM intensity and mitochondrial respiration, while SC9 significantly improved TMRM intensity and mitochondrial respiration (Fig. 3B, C). Similarly, the increased oxidative stress that caused by USP33 silencing was also alleviated by SC9 treatment (Fig. 3D).

Next, we explored the effects of SC9 on endothelial function under HG/FFA conditions. USP33 silencing decreased cell viability and increased LDH content after HG/FFA injury, whereas these effects were reversed by SC9 (Fig. 3E). VEGF and NO promote endothelial migration and angiogenesis. USP33 silencing further inhibited cell migration, NO synthesis, as well as VEGFR2 and eNOS phosphorylation after HG/FFA injury, the effects of which were largely alleviated by SC9 treatment (Fig. 3F–I). Endothelial cells constitute for the vascular barrier for maintain permeability and exchange of nutrient. USP33 silencing intensified endothelial barrier dysfunction as shown by notably increased FITC-dextran permeation, decreased TEER, and enhanced phosphorylation of VE-cadherin under HG/FFA conditions (Fig. 3J, K). In contrast, SC9 treatment improved endothelial barrier function (Fig. 3J, K). In addition, USP33 silencing promoted endothelial-related inflammation makers and insulin-resistant states as evidenced by increased VCAM-1 and ICAM-1 expression, upregulated phosphorylation of IRS1 at S612, and suppressed phosphorylation of AKT1 at S473 after HG/ FFA injury. However, these effects could be reversed by SC9 (Fig. S3A, B). These results indicated that USP33 maintains endothelial function under diabatic conditions, which were potential depended on the inhibition of FIS1-mediated mitochondrial fission.

### USP33 knock-out impaired microvascular function by regulating FIS1 in DCM

**Fig. 4:**
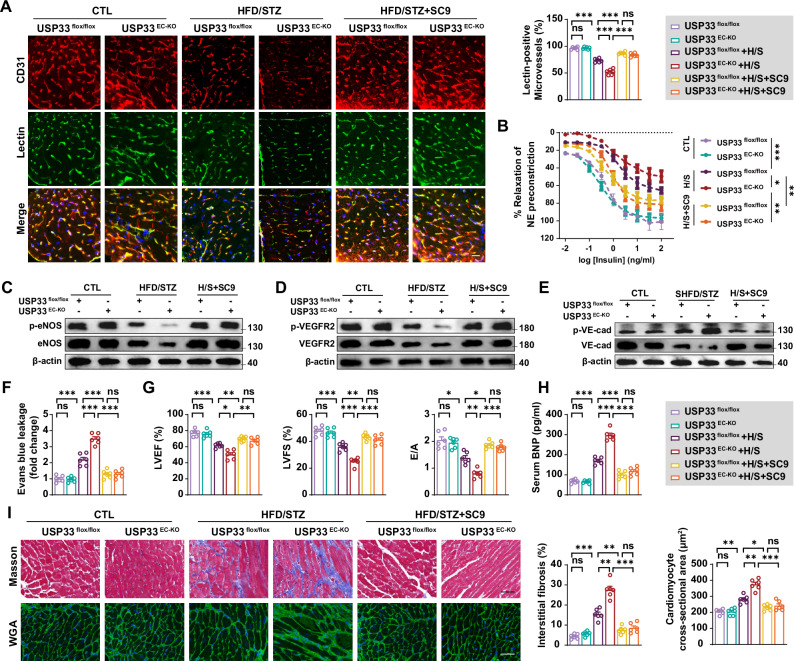
USP33 knock-out impaired microvascular function by regulating FIS1 in DCM. Endothelial-specific deletion of USP33 knockout mice (USP33^EC-KO^) mice and Littermate control (USP33^flox/flox^) mice were subjected to HFD/STZ method to establish diabetic models. SC9 (10 mg/kg) treatment was added to block FIS1-mediated mitochondrial fission. **A** Cardiac microvascular density was assessed by immunofluorescence staining of CD31, and microvascular blood flow was assessed by a lectin-FITC perfusion assay. Scale bar = 25 μm. **B** Endothelium-dependent aortic vasodilation to insulin was assessed using wire myography. **C**–**E** Total and phosphorylated eNOS, VEGFR2 and VE-cadherin expression were detected by western blot. **F** Quantitative analysis of EB leakage assay. **G** Quantitative analysis of the LVEF, LVFS, and E/A ratio by echocardiography. **H** Quantitative analysis of serum BNP levels. **I** Cardiac fibrosis and cardiomyocyte cross-sectional area were detected by Masson trichrome staining and WGA staining, respectively. Scale bar = 70 μm. *p < 0.05, **p < 0.01, ***p < 0.001 indicate significant differences. Four to six biological replicates were performed, and the results are indicated in scatter plots.

To further investigate the mechanisms of USP33 deficiency on microvascular dysfunction in vivo, *USP33*^flox/flox^ mice and *USP33*^EC-KO^ mice were generated and subjected to HFD/STZ method for the establishment of T2DM mice (Fig. S4A, B). After long-term diabetes, endothelial-specific knockout of USP33 markedly enhanced insulin resistance, reduced microvascular perfusion, and impaired insulin-dependent vascular relaxation in T2DM mice. These effects were obviously alleviated by SC9 treatment, which also restored insulin signaling, as evidenced by reduced IRS1 Ser612 phosphorylation and increased AKT1 Ser473 phosphorylation (Fig. 4A, B, and Fig. S4C). In addition, cardiac NO content and phosphorylation of eNOS and VEGFR2 were evidently upregulated by SC9 treatment in both *USP33*^flox/flox^ and *USP33*^EC-KO^ mice after long-term diabetes (Fig. 4C, D, and Fig. S4C). More importantly, vascular leakage, VE-cadherin phosphorylation, and vascular adhesion factors expression were accentuated in *USP33*^EC-KO^ T2DM mice, effects that were relieved by SC9 treatment (Fig. 4E, F, and Fig. S4E).

Attempts were then made to discern whether endothelial USP33 deletion-induced microvascular disorder could exacerbate cardiac dysfunction and pathological remodeling, with a focus on FIS1-mediated mitochondrial fission. The echocardiography results suggested USP33 knockout aggravated cardiac dysfunction, as evidenced by further decreased left LVEF, LVFS and E/A ratio, as well as increased serum BNP level in T2DM mice (Fig. 4G, H). However, SC9 treatment significantly improved cardiac dysfunction in T2DM mice (Fig. 4G, H). Besides, USP33 knockout increased myocardial interstitial fibrosis and contributed to cardiac hypertrophy after long-term diabetes, whereas SC9 treatment alleviated the pathological remodeling (Fig. 4I). These results revealed that endothelial USP33 knockout exerts negative effects on microvascular homeostasis and cardiac function inDCM, with FIS1-mediated mitochondrial fission as one of the potential underly mechanisms.

### USP33 UCH domain interacted with FIS1 at the TPR2 domain

**Fig. 5:**
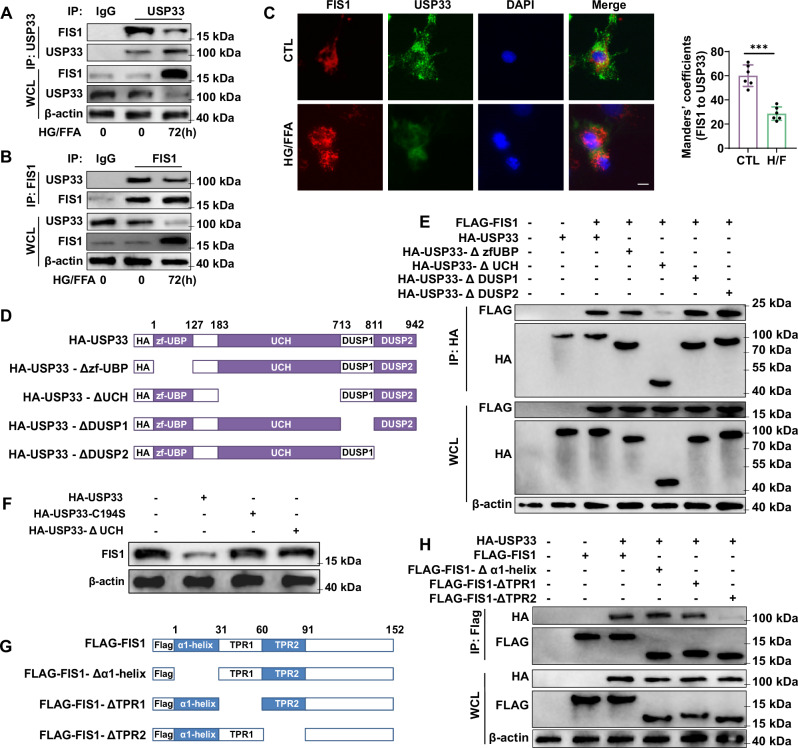
USP33 UCH domain interacted with FIS1 at the TPR2 domain. **A**, **B** HCMECs cell lysates were co-immunoprecipitated with anti-USP33 and anti-FIS1 antibody, respectively. Immunoprecipitates were analyzed by western blot as indicated. **C** The co-localization of FIS1 and USP33 were evaluated by immunofluorescence staining in HCMECs, with or without HG/FFA injury. Scale bar = 10 μm. **D** Schematic illustration of human HA-tagged USP33 WT and mutants. **E** HCMECs were transfected with plasmids expressing FLAG-tagged FIS1, HA-tagged USP33 WT, and HA-tagged USP33 mutants. HCMECs cell lysates were immunoprecipitated with anti-HA antibody, and the immunoprecipitates were analyzed by western blot as indicated. **F** HCMECs were transfected with plasmids expressing HA-USP33 WT, HA-USP33-△UCH mutant, and USP33-C194S mutant for 48 h. The expression of FIS1 were detected by western blot. **G** Schematic illustration of human FLAG-tagged FIS1 WT and mutants. **H** HCMECs were transfected with plasmids expressing HA-tagged USP33, FLAG-tagged FIS1 WT, and FLAG-tagged FIS1 mutants for 48 h. HCMECs cell lysates were immunoprecipitated with anti-FLAG antibody, and the immunoprecipitates were analyzed by western blot as indicated. ***p < 0.001 indicate significant differences. Four to six biological replicates were performed, and the results are indicated in scatter plots.

Co-IP assay in HCMECs showed USP33 and FIS1 were mutually combined (Fig. 5A, B). However, this combination was reduced after HG/FFA injury (Fig. 5A, B). Consistently, immunofluorescence staining showed that FIS1 co-localized with USP33 at the cytoplasm, which was reduced under HG/FFA conditions (Fig. 5C). To identify which domain of USP33 is required for the interaction with FIS1, we constructed HA-tagged USP33 and its truncated mutants (USP33-Δzf-UBP, USP33-ΔUCH, USP33-ΔDUSP1 and USP33-ΔDUSP2), and co-transfected them with FLAG-FIS1 in HCMECs. Co-IP results showed that FLAG-FIS1 failed to be immunoprecipitated by HA-USP33-ΔUCH (loss of residues 183–713) (Fig. 5D, E). In addition, HA-USP33-ΔUCH overexpression failed to downregulate FIS1 expression, the result of which is similar to overexpression of inactive USP33 (HA-USP33-C194S; catalytic inactive USP33 mutant) (Fig. 5F). The above results indicate an importance role of UCH domain in USP33-FIS1 interaction. Next, we constructed FLAG-tagged FIS1 and its truncated mutants (FIS1-Δα1-helix, FIS1-ΔTPR1, and FIS1-ΔTPR2), and co-transfected them with HA-USP33 into HCMECs. Co-IP assay revealed that MYC-USP33 failed to be immunoprecipitated by FLAG-FIS1-ΔTPR2 (loss of residues 60-91) (Fig. 5G, H). Taken together, the above data demonstrated USP33 and FIS1 are mutually interacted via their UCH and TPR2 domains.

### USP33 promoted FIS1 degradation via autophagy-lysosome pathway

**Fig. 6:**
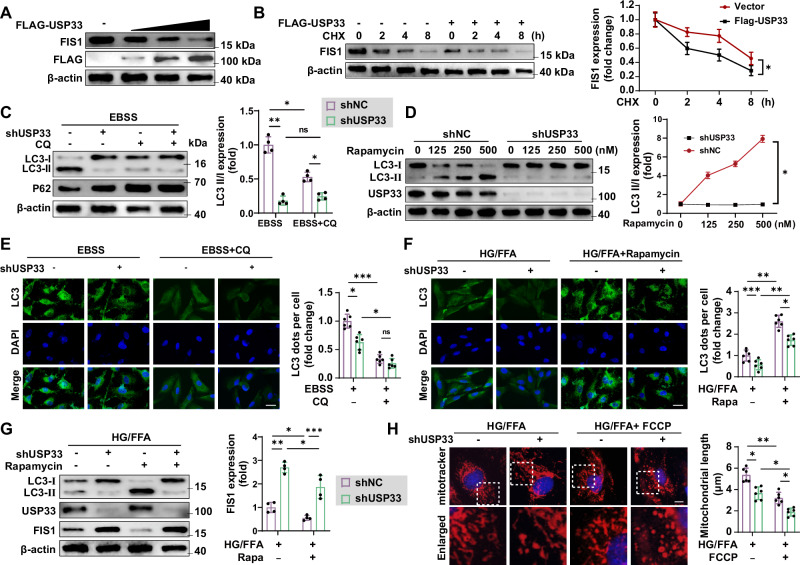
USP33 promoted FIS1 degradation via autophagy-lysosome pathway. **A** HCMECs were transfected with LV-USP33 (10, 30, 50 MOI) for 48 h and exposed to HG/FFA conditions for 72 h. The protein expression of FIS1 was detected by western blot. **B** HCMECs were transfected with FLAG-USP33 and treated with CHX (100 μM) for 8 h to block protein synthesis. The degradation kinetics of FIS1 were monitored over time by western blot. **C** HCMECs were treated with Earles balanced salt solution (EBSS) for 12 h to induce autophagy. The expression of P62 and the ratio of LC3II /LC3I were detected by western blot. **D** HCMECs were treated with rapamycin at indicated concentrations for 12 h, with or without LV-shUSP33 transfection. The ratio of LC3II /LC3I was detected by western blot. **E** HCMECs treated with EBSS in the presence or absence of CQ treatment. Immunofluorescent staining was used to detect LC3 puncta and monitor the autophagic flux. Scale bar = 20 μm. **F** HCMECs were subjected to HG/FFA conditions in the presence or absence of rapamycin (500 nM, 2 h) treatment. Immunofluorescent staining was used to detect LC3 puncta and monitor the autophagic flux. Scale bar = 20 μm. **G** The expression of LC3, USP33 and FIS1 were detected by western blot. **H** HCMECs transfected with LV-shUSP33 cells and subjected to HG/FFA conditions in the presence or absence of FCCP (1 μM, 6 h) treatment. The mitochondrial morphology of HCMECs was visualized by MitoTracker Red staining, and mitochondrial length was quantified. Scale bar = 5 μm. *p < 0.05, **p < 0.01, ***p < 0.001 indicate significant differences. Four to six biological replicates were performed, and the results are indicated in scatter plots.

Based on our findings, we hypothesized that USP33 could interact with and promote FIS1 degradation. For further verification, FLAG-USP33 was transfected to HCMECs, with or without the protein synthesis inhibitor CHX treatment. Immunoblot analysis indicated that FIS1 protein levels were de-escalated in a USP33 dose-dependent manner under HG/FFA treatment (Fig. 6A). In addition, CHX-chase assays showed USP33 overexpression significantly decreased the half-life of FIS1 protein in HCMECs (Fig. 6B). Protein degradation predominantly involves the ubiquitin-proteasome pathway and autophagy-lysosome pathway[31]. Immunoblot analysis showed the USP33-induced FIS1 downregulation only could be reversed by lysosomal inhibitor chloroquine (CQ), rather than proteasome inhibitor MG132 (Fig. S5A). Therefore, the above evidence collectively supported USP33 enhanced FIS1 protein degradation in an autophagy-lysosome pattern.

HG/FFA injury significantly increased the number of autophagosomes (yellow dots) while maintaining a low count of autolysosomes (red dots), indicating impaired autophagic flux at the degradation stage (Fig. S5C). Conversely, USP33 overexpression increased autolysosome formation, reduced autophagosome accumulation, and enhanced p62/SQSTM1 degradation, suggesting that USP33 promotes autophagic flux (Fig. S5B, C). To further determine a role of USP33 in autophagy, Earle’s balanced salt solution (EBSS) and rapamycin were added to induce autophagy in HCMECs. USP33 silencing and CQ markedly suppressed autophagy as evidenced by reduced LC3-II/LC3-I ratio and increased P62 expression (Fig. 6C). Similarly, the dosage-dependent autophagy activation by rapamycin was almost abolished by silencing USP33 (Fig. 6D). These results were further verified by immunofluorescent staining of LC3 puncta to monitor the autophagic flux. USP33 silencing and CQ treatment significantly reduced LC3 puncta formation under EBSS-treated condition (Fig. 6E). In contrast, Rapamycin boosted autophagic flux under HG/FFA injury, but the effect was negated by silencing USP33 (Fig. 6F). Furthermore, the protein level of FIS1 was significantly reduced by rapamycin-induced autophagic flux under HG/FFA injury, whereas USP33 silencing recovered FIS1 expression (Fig. 6G). In addition, USP33 silencing promoted mitochondrial fission under HG/FFA or HG/FFA + FCCP conditions (Fig. 6H).

Mitophagy and CMA represent two distinct pathways of autophagy for selective protein degradation. We observed that FIS1 expression was significantly downregulated by the mitophagy agonist MA5, but barely unaffected by the CMA agonist AR7 (Fig. S6A). Furthermore, HG/FFA injury suppressed mitophagy, as evidenced by reduced mt-Keima signals, downregulated Parkin and PINK1 expression, and inhibited mitochondrial translocation of Parkin (Fig. S6B–E). These pathological effects were effectively alleviated by USP33 overexpression (Fig. S6B–E). Collectively, these results suggested that USP33 enhances autophagy, especially mitophagy, to facilitate FIS1 degradation, thereby inhibiting FIS1-mediated mitochondrial fission under diabetic conditions.

### USP33 interacted with ATG7 to promote autophagy

**Fig. 7:**
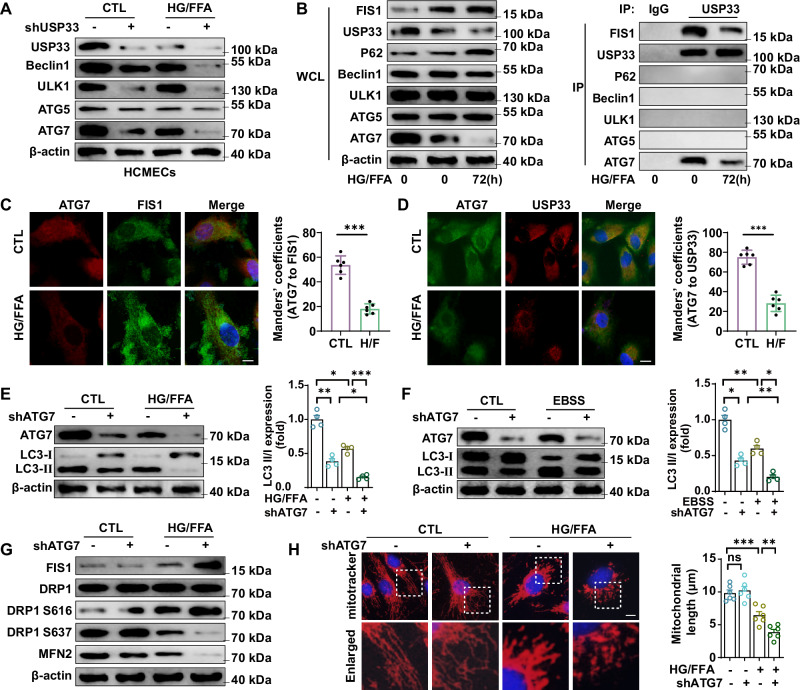
USP33 interacted with ATG7 to promote autophagy. **A** HCMECs were transfected with LV-shUSP33 for 48 h and exposed to HG/FFA conditions for 72 h. The protein expression of Beclin1, ULK1, ATG5 and ATG7 were detected by western blot. **B** HCMECs cell lysates were immunoprecipitated with anti-USP33 antibody. Immunoprecipitates were analyzed via western blot. **C**, **D** HCMECs were exposed to HG/FFA conditions for 72 h followed by immunofluorescent staining for the co-localization among ATG7, FIS1 and USP33. Scale bar = 5 μm. The Manders’ coefficients were statistically analyzed. **E**, **F** HCMECs were transfected with LV-shATG7 for 48 h, and exposed to HG/FFA conditions for 72 h or EBSS treatment for 12 h. The ratio of LC3II /LC3I was detected by western blot. **G** HCMECs were transfected with LV-shATG7 for 48 h and exposed to HG/FFA conditions for 72 h. The expression of FIS1, DRP1 and MFN2, as well as the phosphorylation of DRP1 at Ser 616 and Ser 637 were analyzed by western blot. **H** HCMECs transfected with LV-shATG7 for 48 h and exposed to HG/FFA conditions for 72 h. The mitochondrial morphology of HCMECs was visualized by MitoTracker Red staining, and mitochondrial length was quantified. Scale bar = 5 μm. *p < 0.05, **p < 0.01, ***p < 0.001 indicate significant differences. Four to six biological replicates were performed, and the results are indicated in scatter plots.

Previous studies have reported that autophagy-related proteins participate in the regulation of mitochondrial dynamics [32]. Immunoblot analysis showed that USP33 silencing led to decreased expression of ULK1, Beclin1 and ATG7 in HCMECs, while the expression of ATG5 were unchanged (Fig. 7A). Co-IP analysis revealed that only ATG7 interacted with USP33, but the interaction was suppressed under HG/FFA conditions (Fig. 7B). Immunofluorescent staining further confirmed that ATG7 co-localized with FIS1 and USP33 in HCMECs, while HG/FFA injury reduced their co-localization (Fig. 7C, D). Moreover, ATG7 silencing significantly decreased the ratio of LC3-II/LC3-I under HG/FFA conditions (Fig. 7E). Under autophagy-induced conditions with EBSS, ATG7 silencing decreased the expression of LC3-II, while ATG7 overexpression enhanced the expression of LC3-II in HCMECs (Fig. 7F and Fig. S7A). In addition, ATG7 silencing markedly elevated the expression of FIS1 and phosphorylation of Drp1 at Ser 616 under HG/FFA conditions, suppressed the expression of MFN2 and phosphorylation of Drp1 at Ser 637, and intensified mitochondrial fragmentation under HG/FFA conditions (Fig. 7G, H, and Fig. S7B). As we previous reported, the overexpression of USP33 decreased FIS1 expression, whereas the effect was reversed by ATG7 knockdown (Fig. S7C). In contrast, ATG7 overexpression enhanced mitophagy, reduced FIS1 expression, and inhibited mitochondrial fission (Fig. S7D–F). Taken together, these results revealed that USP33 interacts with ATG7 to negatively regulate FIS1-mediated mitochondrial fission via autophagy.

### USP33 blocked K63-linked ubiquitination of ATG7 at K48

**Fig. 8:**
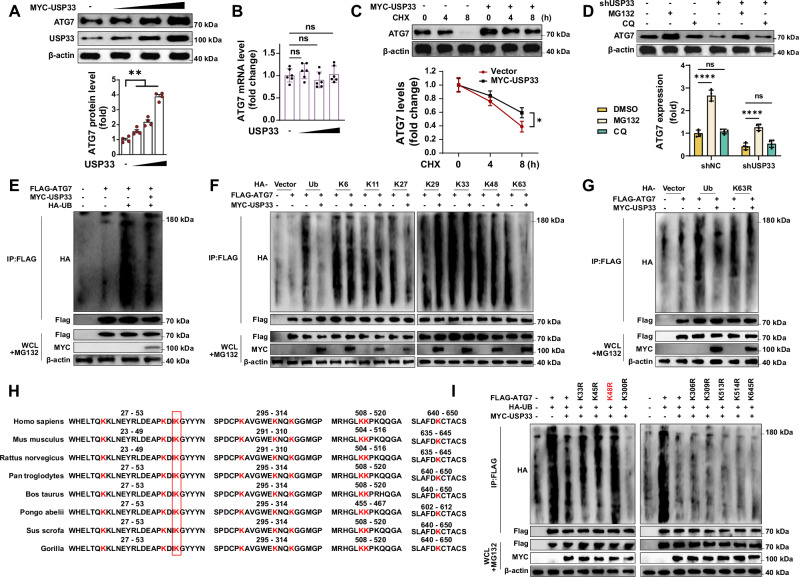
USP33 blocked K63-linked ubiquitination of ATG7 at K48. **A**, **B** HCMECs were transfected with LV-USP33 (10, 30, 50 MOI) for 48 h and exposed to HG/FFA conditions for 72 h. The protein and mRNA expression of ATG7 was detected by western blot and PCR. **C** HCMECs were transfected with LV-USP33 and treated with CHX (100 μM) for 0-8 h to impair protein synthesis. The degradation kinetics of ATG7 was monitored over time by western blot analysis. **D** HCMECs were transfected with LV-shUSP33 and treated with MG132 (10 μM, 4 h) or CQ (10 μM, 4 h). The protein expression of ATG7 was detected by western blot. **E** HCMECs were transfected with plasmids expressing FLAG-tagged ATG7, MYC-tagged USP33 and HA-tagged UB before the cell lysates were immunoprecipitated with anti-FLAG antibodies. Immunoprecipitates were analyzed by western blot. **F** HCMECs were transfected with plasmids expressing FLAG-tagged ATG7, MYC-tagged USP33 and HA-tagged UB (WT, K6-only, K11-only, K27-only, K29-only, K33-only, K48-only, K63-only) before the cell lysates were immunoprecipitated with anti-FLAG antibodies. Immunoprecipitates and input were analyzed by western blot. **G** HCMECs were transfected with plasmids expressing FLAG-tagged ATG7, MYC-tagged USP33 and HA-tagged UB (WT or K63R) before the cell lysates were immunoprecipitated with anti-FLAG antibodies. Immunoprecipitates and input were analyzed by western blot. **H** Conserved lysine residues of ATG7 in the indicated species. **I** Plasmids expressing HA-Ub, MYC-USP33, FLAG-ATG7 and FLAG-ATG7 mutants were co-transfected into HCMECs followed by Co-IP. Immunoprecipitates were analyzed by western blot. *p < 0.05, **p < 0.01, ***p < 0.001 indicate significant differences. Four to six biological replicates were performed, and the results are indicated in scatter plots.

As we previous demonstrated USP33 silencing caused further decreased ATG7 expression under both control and HG/FFA conditions, further attempts were made to investigate how USP33 upregulates ATG7. We found that USP33 increased protein stability of ATG7 in a dose-dependent manner under HG/FFA treatment, but didn’t affect the its mRNA level (Fig. 8A, B). CHX-chase assays showed USP33 overexpression significantly inhibited ATG7 protein degradation (Fig. 8C). Furthermore, proteasome inhibitor MG132 promoted ATG7 stability, even USP33 was knocked-out (Fig. 8D). While autolysosome inhibitor CQ failed to stabilize ATG7 (Fig. 8D). Hence, the above results indicate USP33 stabilized ATG7 by inhibiting ubiquitin-proteasome pathway.

Considering that USP33 functions as a DUB and we therefore attempt to demonstrate USP33 may participate in the ubiquitination of ATG7. We co-transfected HA-tagged ubiquitin (HA-Ub), FLAG-ATG7 and MYC-USP33 into HCMECs followed by Co-IP assay. Immunoblot analysis showed that ATG7 ubiquitination was significantly inhibited by USP33 overexpression (Fig. 8E). It has been well-established that diverse polyubiquitin linkages regulate different biological activities, thus a series of ubiquitin mutants (K6, K11, K27, K29, K33, K48 and K63) were then constructed[33], and transfected to HCMECs. Notably, USP33 overexpression predominantly blocked K63-linked ubiquitination of ATG7 (Fig. 8F). Furthermore, we replaced the lysine K63 of ubiquitin with arginine (K63R), which obviously reduced the ubiquitination of ATG7, even without USP33 overexpression (Fig. 8G). The above data indicated K63 ubiquitination might be the major ubiquitination form for USP33-mediated ATG7 stabilization. Next, we searched the potential lysine residues of ATG7 for K63 ubiquitination, and identified 9 conserved lysine residues (K33, K45, K48, K300, K306, K309, K513, K514, K645) across multiple species (Fig. 8H). Then, we replaced the above lysine residues of ATG7 with arginine, and found only the K48R blocked USP33-induced de-ubiquitination of ATG7 (Fig. 8I). Collectively, USP33 stabilized ATG7 at protein degradation level by blocking K63-linked ubiquitination of ATG7 at K48 site.

### K48 mutant abolished the beneficial effects of ATG7 on mitochondrial and endothelial function

**Fig. 9:**
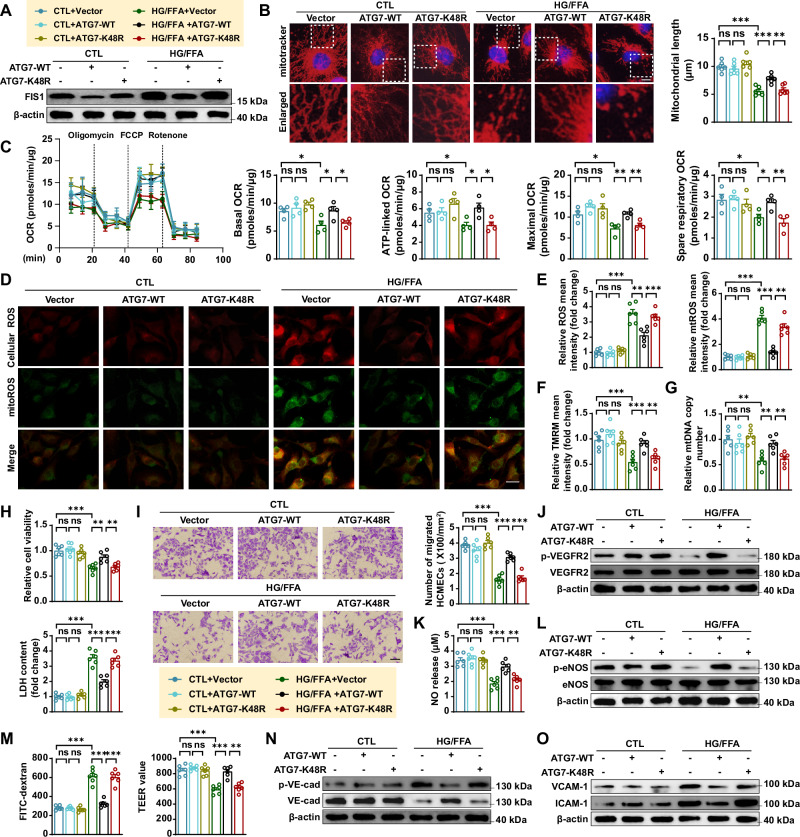
K48 mutant abolished the beneficial effects of ATG7 on mitochondrial and endothelial function. HCMECs were transfected with ATG7-wild-type (WT) or ATG7-K48R mutant for 48 h and subjected to HG/FFA injury for 72 h. **A** The FIS1 expression was verified by western blot. **B** The mitochondrial morphology of HCMECs was visualized by MitoTracker Red staining, and mitochondrial length was quantified. Scale bar = 5 μm. **C** The mitochondrial respiratory activity of HCMECs was assessed using a Seahorse analyzer. The baseline OCR, ATP-linked OCR, maximal OCR and spare respiratory OCR were quantitatively analyzed. **D**, **E** Intracellular ROS (green) and mitoROS (red) were photographed and quantitatively analyzed. Scale bar = 20 μm. **F** The mitochondrial membrane potential of HCMECs was detected by TMRM staining. **G** Statistical analysis of the mtDNA copy number of HCMECs. **H** Relative cell viability and LDH content were statistically analyzed. **I** Representative images of the transwell assay and statistical analysis of migrated cells. Scale bars = 100 μm. **J** Total and phosphorylated VEGFR2 expression was detected by western blot. **K** Statistical analysis of NO release in supernatant. **L** Total and phosphorylated eNOS expression was detected by western blot. **M** Statistical analysis of FITC-dextran permeability and TEER value in indicated groups. **N** Total and phosphorylated VE-cadherin expression was detected by western blot. **O** Protein expression of VCAM-1 and ICAM-1 was detected by western blot. *p < 0.05, **p < 0.01, ***p < 0.001 indicate significant differences. Four to six biological replicates were performed, and the results are indicated in scatter plots.

To further verify the important role of K48 lysine site of ATG7 on mitochondrial protection, ATG7-wild type (WT) or ATG7-K48R mutant were transfected into HCMECs. The overexpression of ATG7-WT significantly inhibited FIS1 expression and mitochondrial fragmentation, whereas K48R mutant abolished the effect of ATG7 (Fig. 9A, B). Seahorse analysis revealed that ATG7-WT overexpression enhanced basal OCR, ATP-linked OCR, maximal OCR, and spare respiratory capacity under HG/FFA, but ATG7-K48R showed no effects on OCRs (Fig. 9C). Immunofluorescence staining showed that ATG7-WT overexpression markedly decreased the levels of cellular ROS and mtROS, as well as improved TMRM mean intensity and mtDNA copy number under HG/FFA conditions, whereas K48R mutation abolished the benefits of ATG7 (Fig. 9D-G). These results indicated that K48 lysine residue of ATG is essential for mitochondrial protection under diabetic injury.

Further work was made to explore the effect of K48 of ATG7 on endothelial function under HG/FFA conditions. ATG7-WT overexpression enhanced cell viability and decreased LDH content after HG/FFA injury, whereas ATG7-K48R mutation abrogated this effect (Fig. 9H). ATG7-WT overexpression enhanced HCMECs migration, NO release and the phosphorylation of VEGFR2 and eNOS, but these effects were not regained after K48R mutation of ATG7 (Fig. 9I–L). In addition, ATG7-WT overexpression improved endothelial permeability by maintaining VE-cadherin phosphorylation, whereas ATG7-K48R mutation lose these benefits (Fig. 9M, N). Moreover, the expression of vascular adhesion factors (VCAM-1 and ICAM-1) was inhibited by ATG7-WT overexpression, but not via ATG7-K48R overexpression (Fig. 9O). In conclusion, these data suggested that ATG7 improves mitochondrial network and recovers endothelial function under HG/FFA conditions, and identified K48 lysine residue as a functional site for ATG7.

### Mouse K44 mutant impaired the protective effects of ATG7 on microvascular function in vivo

**Fig. 10:**
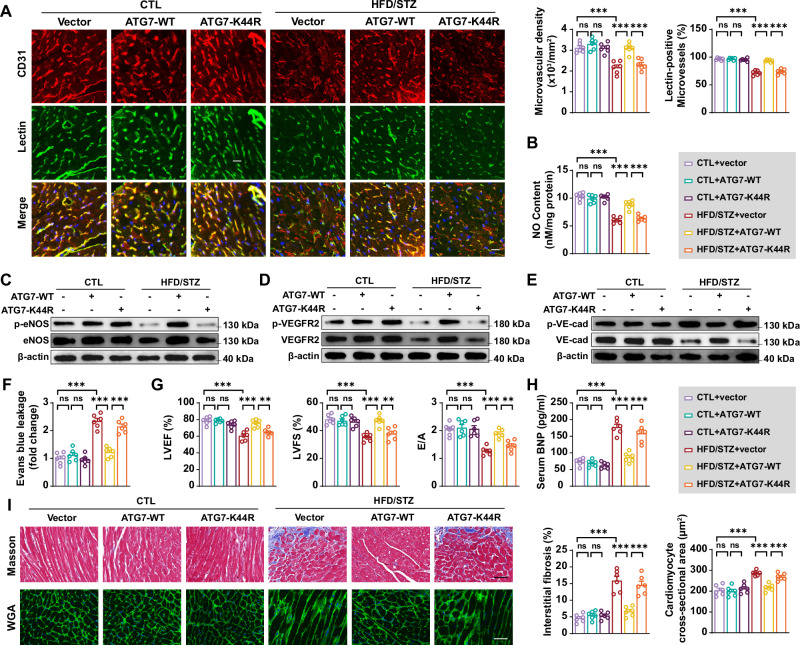
Mouse K44 mutant impaired the protective effects of ATG7 on microvascular function *in vivo.* Endothelial-specific overexpression of mouse ATG7-WT and mouse ATG7-K44R were achieved by AAV9 transfection. **A** Cardiac microvascular density was detected by immunofluorescence staining of CD31, and microvascular blood flow was detected by a lectin-FITC perfusion assay. Scale bar = 25 μm. **B** Quantitative analysis of NO content in myocardial tissues. **C**–**E** Total and phosphorylated eNOS, VEGFR2 and VE-cadherin expression were detected by western blot. **F** Quantitative analysis of EB leakage assay. **G** Quantitative analysis of the LVEF, LVFS, and E/A ratio by Echocardiography. **H** Quantitative analysis of serum BNP levels. **I** Cardiac fibrosis and cardiomyocyte cross-sectional area were detected by Masson trichrome staining and WGA staining, respectively. Scale bar = 70 μm. *p < 0.05, **p < 0.01, ***p < 0.001 indicate significant differences. Four to six biological replicates were performed, and the results are indicated in scatter plots.

Mouse ATG7 K44 is identical to human ATG7 K48 (Fig. 8H). To confirm the benefits of ATG7 on cardiac microvascular protection, AAV9 packaging mouse ATG7-WT and mouse ATG7-K44R were transfected to T2DM. ATG7-WT overexpression increased microvascular density and perfusion, elevated cardiac NO content, and upregulated the phosphorylation of eNOS and VEGFR2 in T2DM mice, whereas ATG7-K44R overexpression failed to improve cardiac microvascular function (Fig. 10A–D). Additionally, ATG7-WT overexpression inhibited vascular leakage and suppressed VE-cadherin phosphorylation, whereas ATG-K44R was not effective on vascular integrity (Fig. 10E, F).

Next, we investigated whether the microvascular protection from ATG7 overexpression improves cardiac dysfunction and pathological remodeling in DCM. The echocardiography results revealed that ATG7-WT overexpression alleviated cardiac systolic and diastolic function in T2DM mice, as evidenced by increased left LVEF, LVFS and E/A ratio, as well as decreased serum BNP level (Fig. 10G, H). Importantly, ATG7-WT overexpression prevented myocardial interstitial fibrosis and reduced cardiac hypertrophy in T2DM mice (Fig. 10I). However, the above benefits of ATG7 on cardiac dysfunction and pathological remodeling was abolished by K44R mutation (Fig. 10G–I). These results demonstrated that mouse K44R mutant impairs the protective effects of ATG7 on cardiac microvascular homeostasis and cardiac function in DCM.

## Discussion

Dysregulated mitochondrial dynamics is a key contributor to cardiovascular diseases, mainly by affecting energetic production, redox homeostasis, calcium signaling, ferroptosis and other mechanisms [34, 35]. Our previous study proposed the pivotal role of mitochondrial fission on cardiac microvascular disturbance of DCM[2]. Considering the essential role of FIS1 in mitochondrial fission, we screened 40 DUBs and identified USP33 as the most powerful candidate for regulating FIS1 expression. USP33 silencing or deficiency significantly upregulated the expression of FIS1, leading to increased endothelial mitochondrial fragmentation and cardiac microvascular dysfunction in DCM. Mechanistically, we found that USP33 promotes FIS1 degradation via deubiquitinating and stabilizing ATG7.

USP33 is closely correlated with tumor development and inflammation-related disorders[36, 37]; however, little is known about the role of USP33 on microvascular disturbance. Emerging evidence suggests that the USP33 plays a pivotal role in the regulation of various biological processes [38–41]. In glioma stem cells, USP33 deubiquitinates and stabilizes HIF-2α to promote hypoxia response [39]. USP33 also restrains docetaxel-induced apoptosis via deubiquitination and stabilization of the phosphatase DUSP1 in prostate cancer [41]. In our study, USP33 regulated the protein stability of FIS1 rather than the mRNA transcription in diabetes. Moreover, pharmaceutical inhibition of FIS1-Drp1 interaction reversed increased mitochondrial fragmentation driven by USP33 silencing, suggesting FIS1-Drp1 acted as downstream targets of USP33 in regulating mitochondrial fission. We found knockdown of USP33 has a minimal effect on mitochondrial fragment under normal conditions where fission is not prominent. In contrast, within the diabetic model, where mitochondrial fission is already elevated, USP33 knockdown suppresses autophagy, impairs the repair and clearance of damaged mitochondria, thereby intensifying mitochondrial fragment and subsequent cardiac injury. This finding is consistent with similar roles reported for other DUBs like USP28 [42].

Autophagy is an intracellular degradation process that dynamically transports proteins and organelles to the lysosome for degradation [43]. At present, mitophagy and CMA are recognized as two selective autophagy process for protein degradation. The present study demonstrated USP33 effectively enhanced autophagy and promoted FIS1 degradation via mitophagy pathway. It has been well-established that autophagy is orchestrated by ubiquitination-like conjugation systems, including the ATG12 conjugation system and the LC3/ATG8 lipidation system, both of which are activated by the same E1-like enzyme ATG7 [44]. ATG7 deficiency severely dampens cellular autophagy, resulting in mitochondrial dysfunction, oxidative stress response, and endoplasmic reticulum stress [45–47]. However, the regulatory role of ATG7 in endothelial function is still controversial. Under ischemic conditions, EC-specific deletion of *Atg7* significantly reduced post-ischemic angiogenesis via blocking HIF1A-mediated STAT1 upregulation in an autophagy-independent manner [22]. In contrast, endothelial *Atg7* knockout ameliorated acute cerebral ischemia/reperfusion injury [48]. In the present study, ATG7 overexpression enhanced endothelial autophagy in diabetes, and therefore promoted FIS1 degradation and balanced mitochondrial dynamic. In addition, endothelial-specific overexpression of ATG7 improved cardiac microvascular function and cardiac function in DCM.

Given the critical role of ATG7 in canonical autophagic pathways, post-translational modifications of ATG7 may play a key regulatory role in autophagic response and EC function. The ubiquitination modification of ATG7 has been reported to regulate its function. Notably, several E3 ubiquitin ligase have been identified to ubiquitinate ATG7 at specific lysine residues to regulate the function of ATG7. Under oxidative stress, E3 ubiquitin ligase TRIM32 mediates K63-linked ubiquitination of human ATG7 at the K45 site to initiate autophagic response [46]. During infection, TRIM7 promoted the K63-linked ubiquitination of human ATG7 at K413, and ubiquitination at this site was required for the function of ATG7 in autophagy [49]. However, de-ubiquitination has not been reported to regulate autophagic responses targeting ATG7. In this study, we identified USP33 as a novel ATG7 deubiquitinase. USP33 blocked K63-linked ubiquitination of human ATG7 at the K48 (mouse K44) site, making K48 site as a determinant site for the stability of ATG7. Furthermore, mouse ATG7-K44 mutant impairs the beneficial effects of ATG7 on cardiac microvascular function in DCM. Herein, we identified USP33 as a pivotal deubiquitinase involved in ubiquitination degradation and autophagic function of ATG7.

Importantly, several potential biases or imprecisions should be considered. The present work relies heavily on pharmacological inhibition of FIS1-Drp1 interaction using SC9. However, a Fis1 knockdown or conditional knockout mouse model may provide more credible evidence for our findings, since the potential off-target effects of SC9 remain unknown. Moreover, mitochondrial morphology analysis should ideally be performed on 3D-rendered z-stacks rather than single optical planes. Besides, the type of ubiquitination must be clearly defined and mechanistically interpreted. K63-linked chains are often linked to signaling and autophagy-related processes. However, in some contexts, K63-linked chains have also been demonstrated to be involved in protein degradation pathways [50]. Finally, the expression of major targets, such as USP33, were not tested in human cardiac or endothelial samples or in larger animals.

To summarize, our findings reveal that USP33 de-ubiquitinates and stabilizes ATG7 to enhance the protein degradation of FIS1 via the enhanced autophagy, which ultimately improves endothelial mitochondrial dynamics and alleviated cardiac microvascular dysfunction. Therefore, we suppose that USP33 may be a promising therapeutic target for the treatment of cardiac microvascular injury of DCM.

## Supplementary information

Supplementary Figure legend

Original Data

## Data Availability

The data that support the findings of this study are available from the corresponding author upon reasonable request.
