# Supplementary material for: USP33 alleviates FIS1-dependent mitochondrial fission and cardiac microvascular injury in diabetic cardiomyopathy via deubiquitinating and stabilizing ATG7
Source: Cell Death Dis. 2026 May 30;17(1):674. doi: 10.1038/s41419-026-08930-8 (PMC13434780; doi:10.1038/s41419-026-08930-8)
Supplement: Supplementary file 1 [file 41419_2026_8930_MOESM1_ESM.pdf]

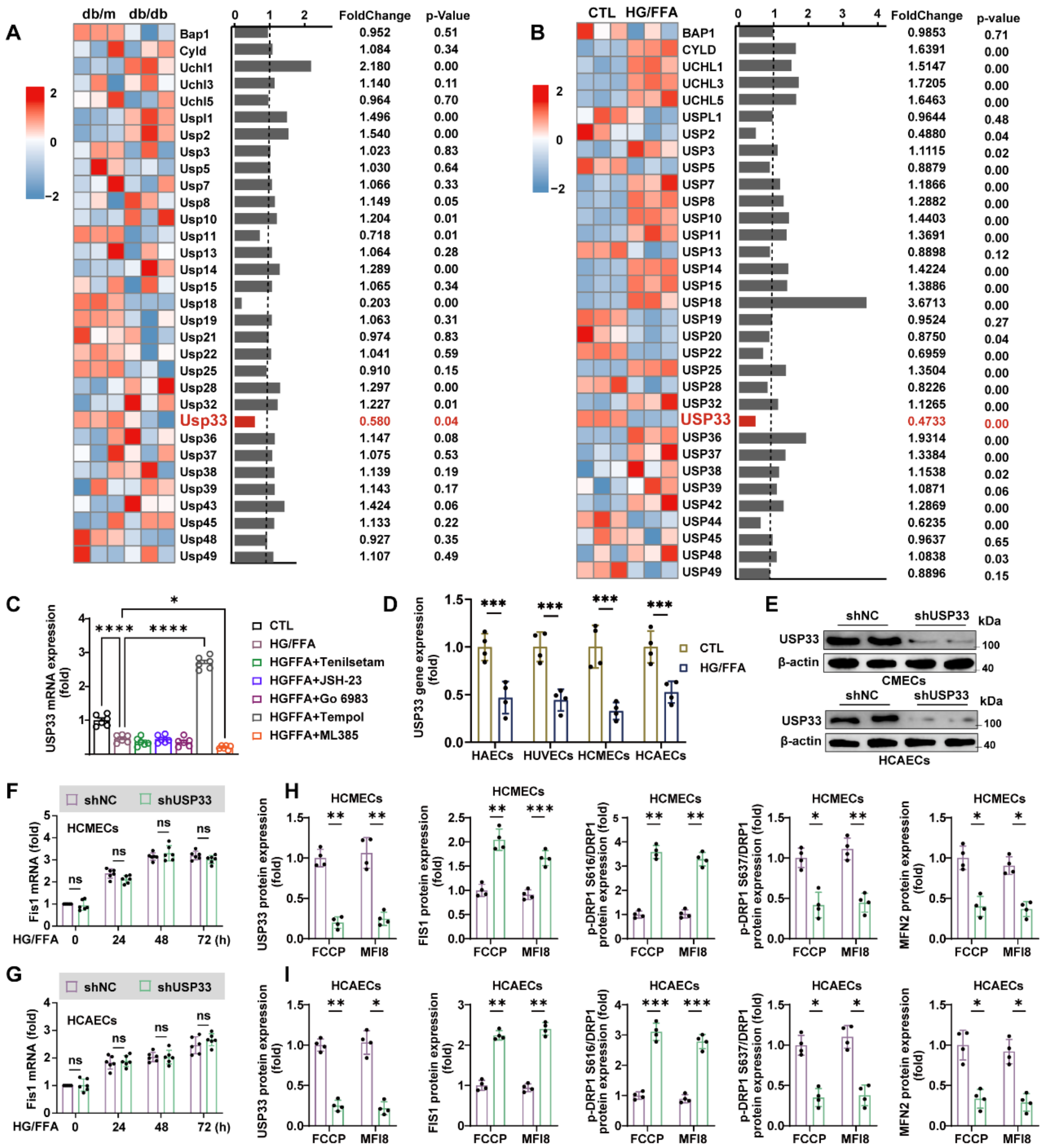

Figure S1. (A-B) The heartmap of DUBs form RNA-Seq datasets of db/db mice and HG/FFA-treated HCMECs. **(C)** The mRNA expression of USP33 in indicated groups in HCMECs. (D) HAECs, HUVECs, HCMECs and HCAECs were subjected to HG/FFA injury for 72h, the mRNA expression of USP33 were analyzed by PCR. **(E)** HCMECs and HCAECs were transfected with LV-shUSP33 for 48h. The transfection efficiency was analyzed by western blot. **(F-G)** HCMECs and HCAECs were transfected with LV-shUSP33 for 48 h, followed by HG/FFA exposure for the indicated

durations. The mRNA expression of FIS1 were assessed by PCR. **(H-I)** The expression of FIS1, USP33, DRP1 and MFN2, as well as the phosphorylation of DRP1 at Ser 616 and Ser 637 were statistically analyzed. *p < 0.05, **p < 0.01, ***p < 0.001 indicate significant differences. Four to six biological replicates were performed, and the results are indicated in scatter plots.

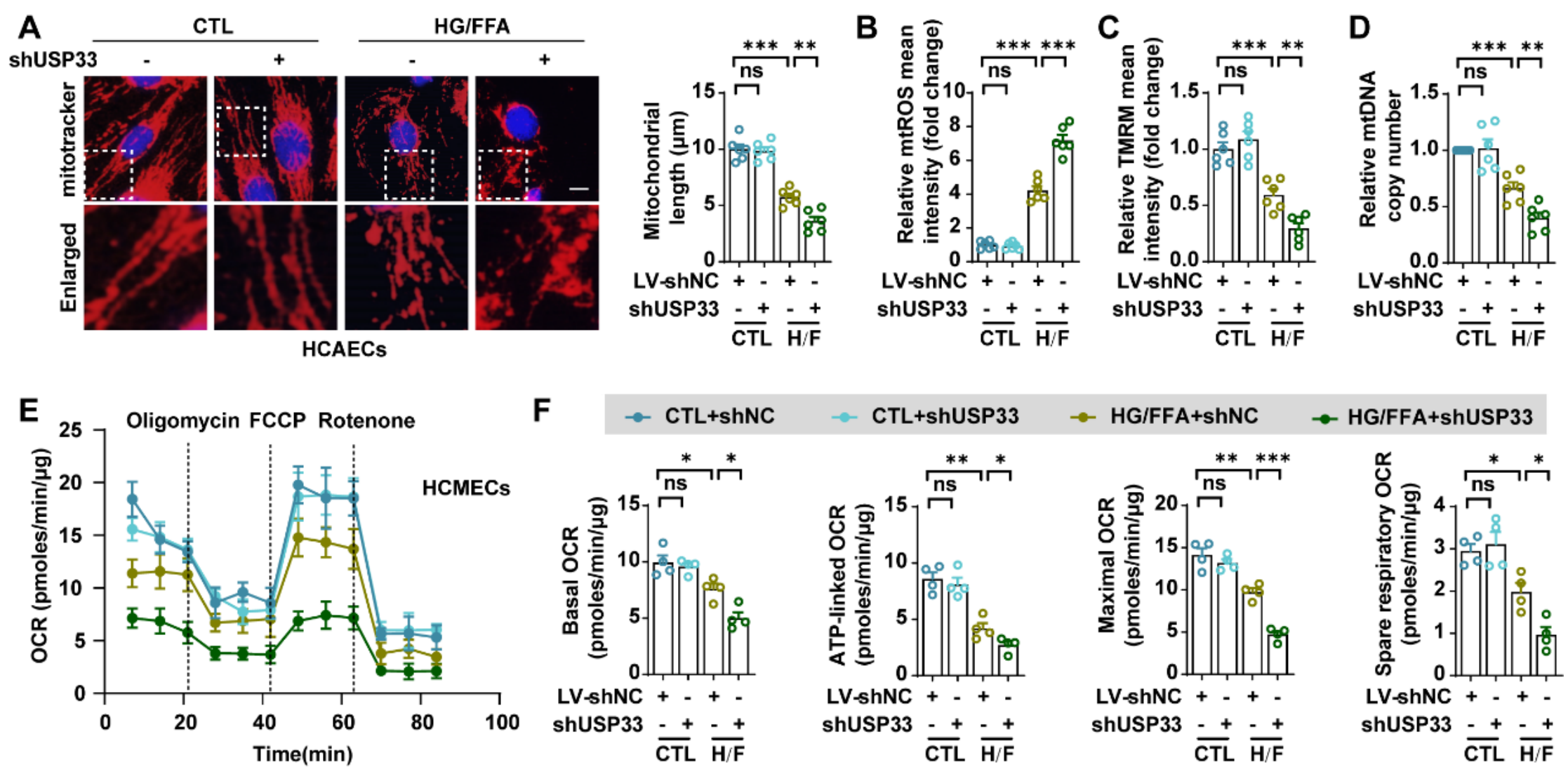

Figure S2. HCAECs were transfected with LV-shUSP33 for 48 h and exposed to HG/FFA injury for 72h. **(A)** The mitochondrial morphology of HCAECs was visualized by MitoTracker Red staining, and mitochondrial length was quantified. Scale bar = 5 μm. **(B-D)** Statistic analysis of mtROS level, mitochondrial membrane potential and mtDNA copy number were detected in HCAECs. **(E-F)** The mitochondrial respiratory activity of HCAECs was assessed using a Seahorse analyzer. The baseline OCR, ATP-linked OCR, maximal OCR and spare respiratory OCR were quantitatively analyzed. *p < 0.05, **p < 0.01, ***p < 0.001 indicate significant differences. Four to six biological replicates were performed, and the results are indicated in scatter plots.

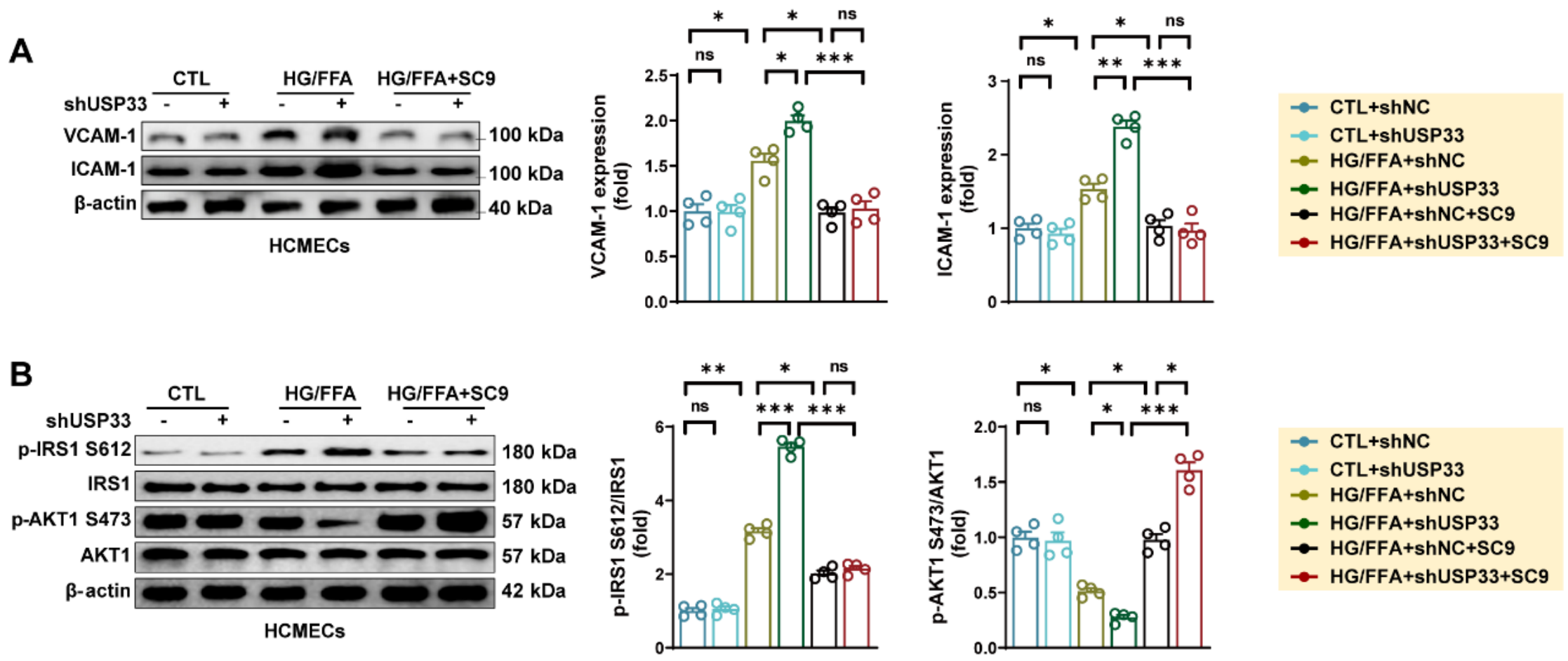

Figure S3. HCMECs were transfected with LV-shUSP33 for 48h, with or without SC9 treatment (2 μM) for 72h. **(A)** The protein expression of VCAM-1 and ICAM-1 was detected by western blot and statistically analyzed. **(B)** The protein expression and phosphorylation of IRS1 and AKT1 was detected by western blot and statistically analyzed. *p < 0.05, **p < 0.01, ***p < 0.001 indicate significant differences. Four biological replicates were performed, and the results are indicated in scatter plots.

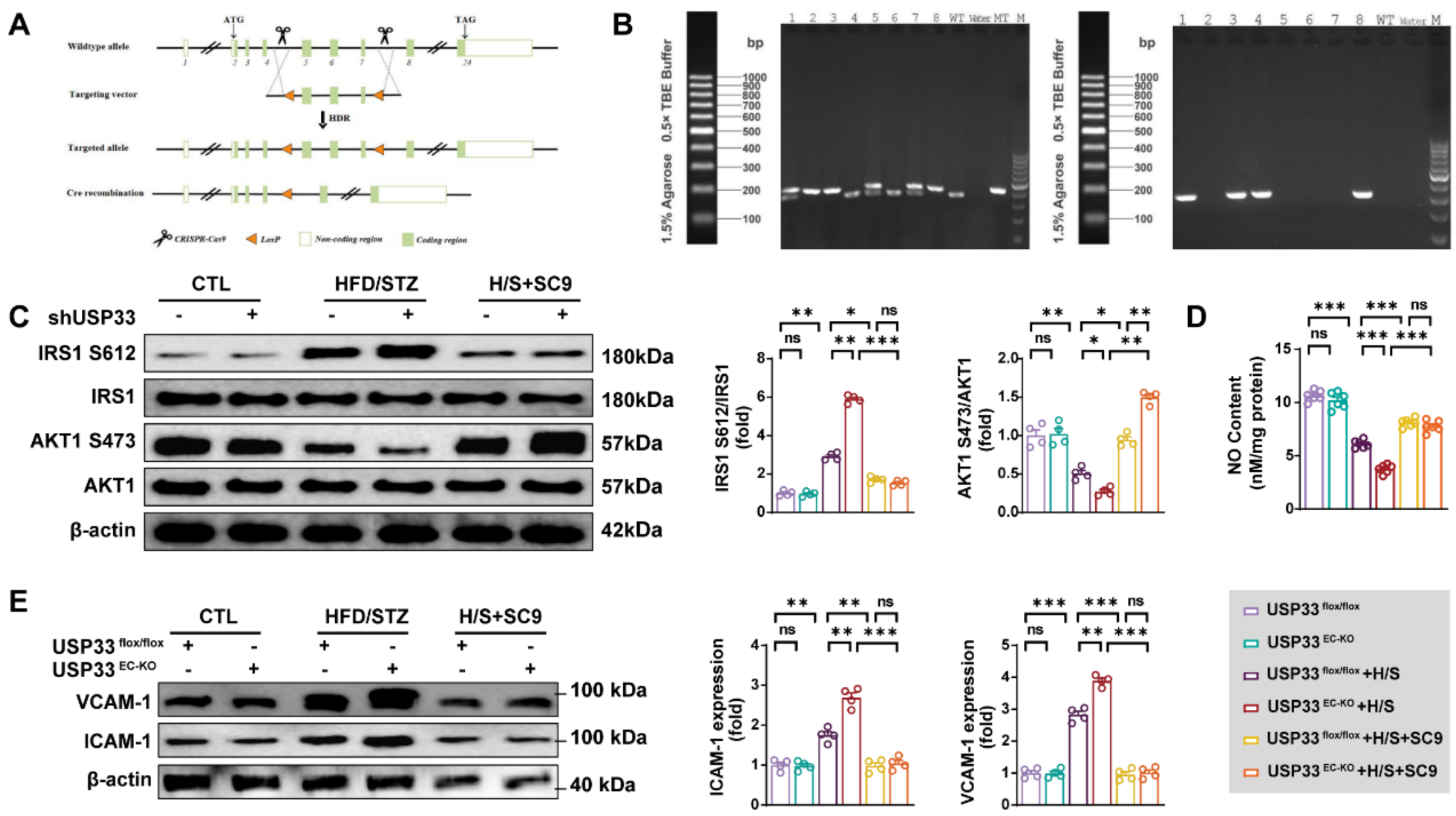

Figure S4. (A) The strategy for constructing endothelial-specific USP33 knockout ($USP33^{EC-KO}$) mice. **(B)** Representative PCR images of USP33-flox (left) and *Cdh5*-Cre^{ERT2} (right) using DNA isolated from $USP33^{flox/flox}$ and $USP33^{EC-KO}$ mice. **(C)** The

protein expression and phosphorylation of IRS1 and AKT1 was detected by western blot and statistically analyzed. **(D)** Quantitative analysis of NO content in myocardial tissues. **(E)** The protein expression of VCAM-1 and ICAM-1 was detected by western blot and statistically analyzed. *p < 0.05, **p < 0.01, ***p < 0.001 indicate significant differences. Four biological replicates were performed, and the results are indicated in scatter plots.

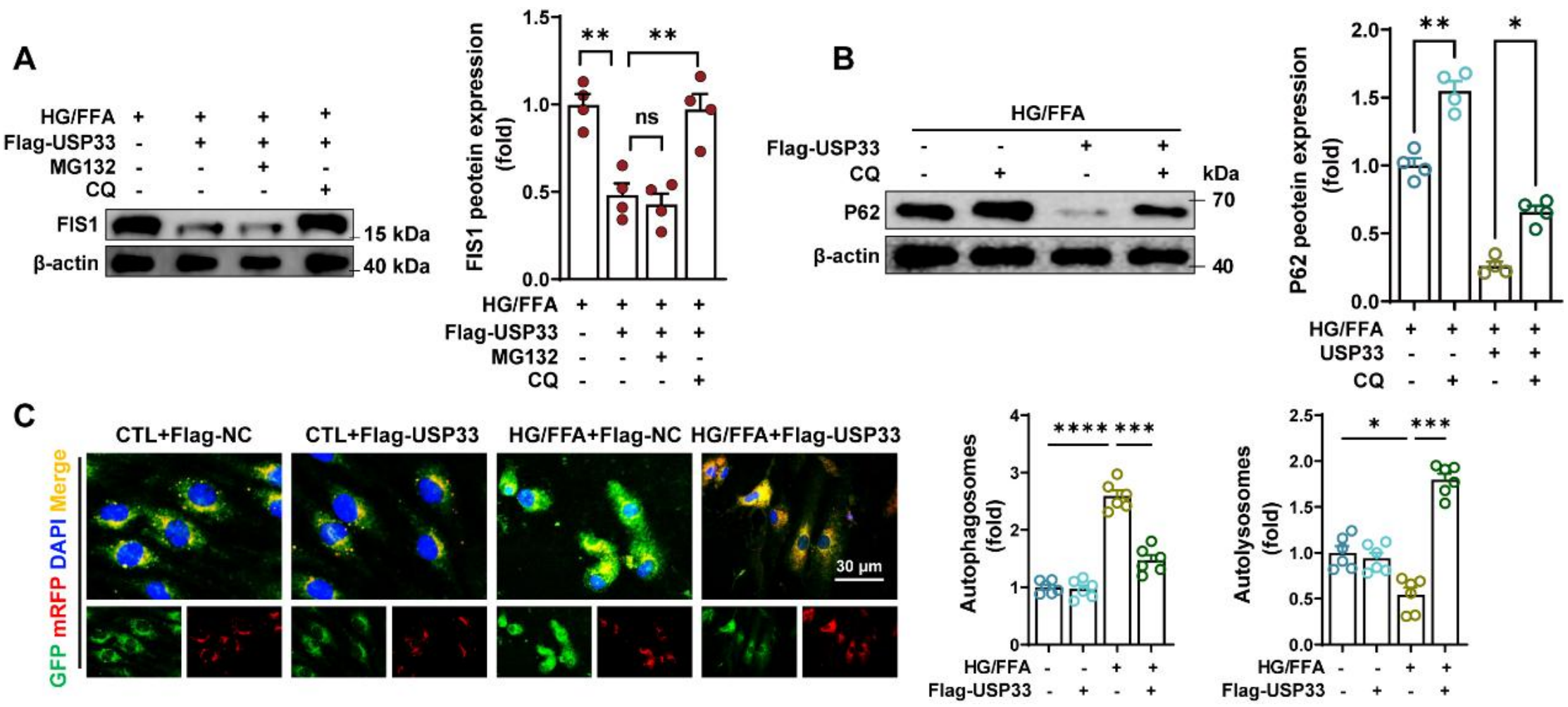

Figure S5. HCMECs transfected with FLAG-USP33 for 24 hours, with or without MG132 (10 μM, 4h) or CQ (10 μM, 4h) treatment. **(A)** The expression of FIS1 was detected by western blot. **(B)** The expression of P62 was detected by western blot. **(C)** Representative images of LV-mRFP-GFP-LC3 transfection. Autophagosomes (yellow puncta) and autolysosomes (red puncta) were counted. Bar = 25 μm. *p < 0.05, **p < 0.01, ***p < 0.001 indicate significant differences. Four biological replicates were performed, and the results are indicated in scatter plots.

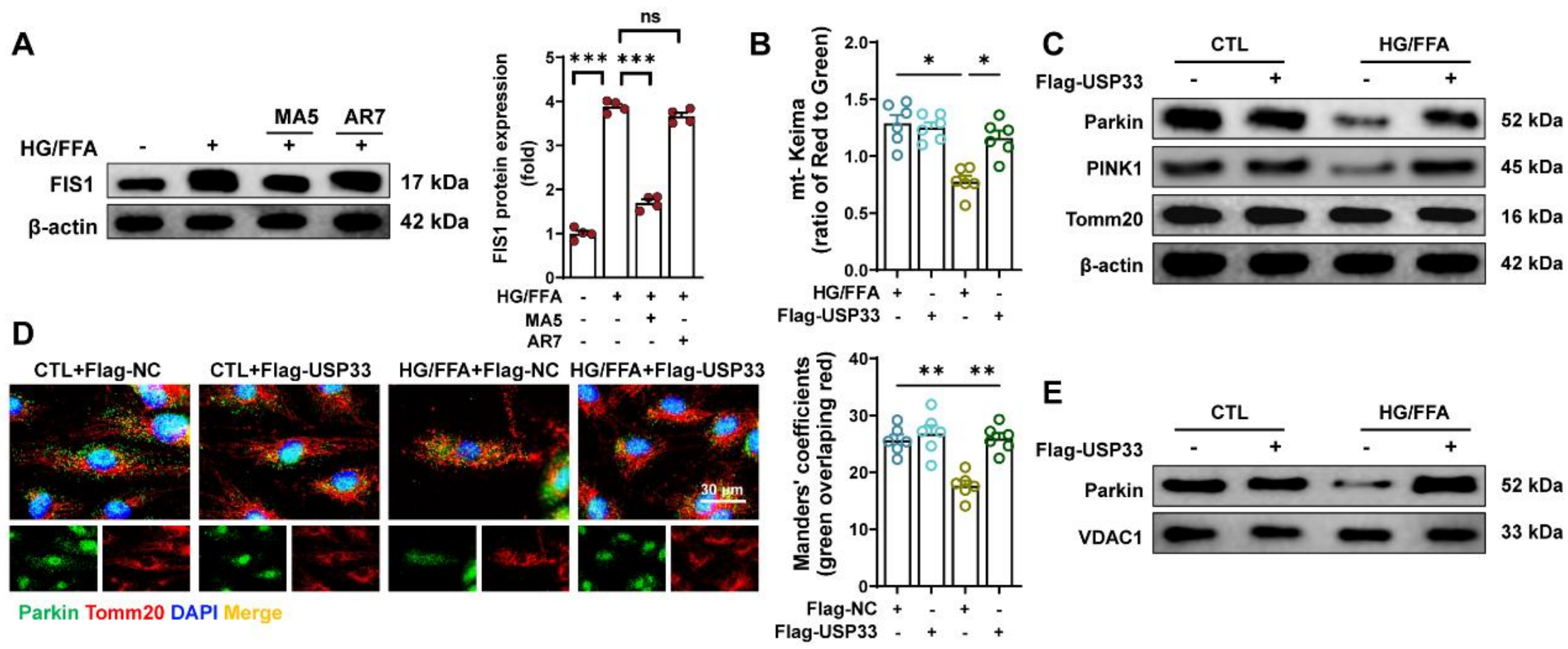

Figure S6. (A) HCMECs were treated with HG/FFA injury for 72h, with or without MA5 (5 μM) or AR7 (5 μM) treatment. The expression of FIS1 was detected by western blot. **(B)** HCMECs were transfected with FLAG-USP33 for 48 h and exposed to HG/FFA injury for 72h. Mitophagy level was detected by mt-Keima transfection and statistically analyzed. **(C)** Protein expression of Parkin, PINK1, and Tomm20 was detected by western blot. **(D)** Colocalization of Parkin and Tomm20 was detected by immunofluorescence staining and quantified by Manders' coefficients. **(E)** Protein expression of Parkin was detected by western blot in isolated mitochondria. *p < 0.05, **p < 0.01, ***p < 0.001 indicate significant differences. Four to six biological replicates were performed, and the results are indicated in scatter plots.

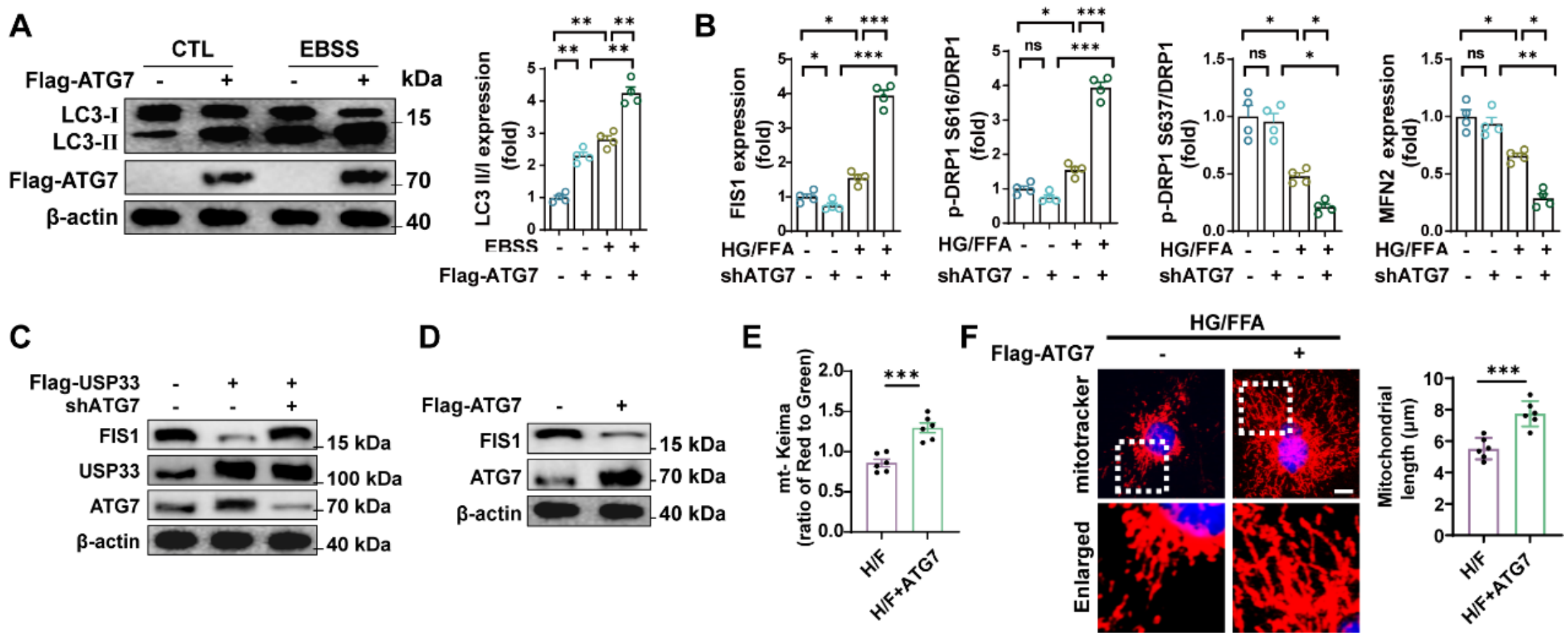

Figure S7. (A) HCMECs were transfected with ATG7 for 24 hours and treated with EBSS for 12h. The ratio of LC3II /LC3I was detected by western blot. **(B)** HCMECs were transfected with LV-shATG7 for 48h, and exposed to HG/FFA conditions for 72h. The expression of FIS1 and MFN2, as well as the phosphorylation of DRP1 at Ser 616 and Ser 637 were statistically analyzed. **(C)** HCMECs were transfected with FLAG-USP33 and shATG7 followed by western blot of FIS1. **(D)** HCMECs were transfected with FLAG-ATG7 for 72h followed by western blot of FIS1. **(E)** Mitophagy level was detected by mt-Keima transfection and statistically analyzed. **(F)** The mitochondrial morphology of HCMECs was visualized by MitoTracker Red staining, and mitochondrial length was quantified. Scale bar = 5 μm. *$p < 0.05$, **$p < 0.01$, ***$p < 0.001$ indicate significant differences. Four to six biological replicates were performed, and the results are indicated in scatter plots.
