# Supplementary material for: USP33 alleviates FIS1-dependent mitochondrial fission and cardiac microvascular injury in diabetic cardiomyopathy via deubiquitinating and stabilizing ATG7
Source: Cell Death Dis. 2026 May 30;17(1):674. doi: 10.1038/s41419-026-08930-8 (PMC13434780; doi:10.1038/s41419-026-08930-8)

Fig1A

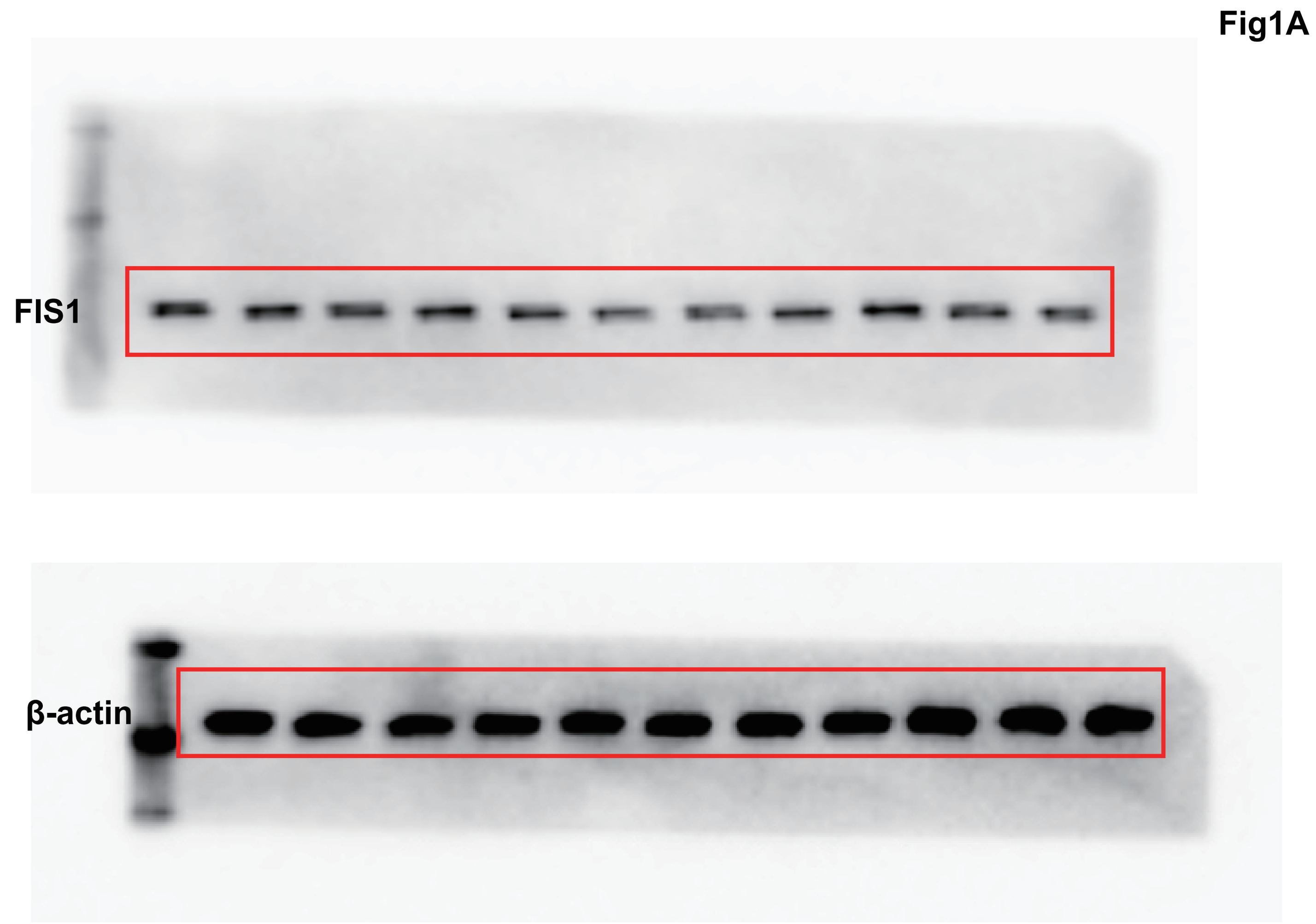

Fig1A

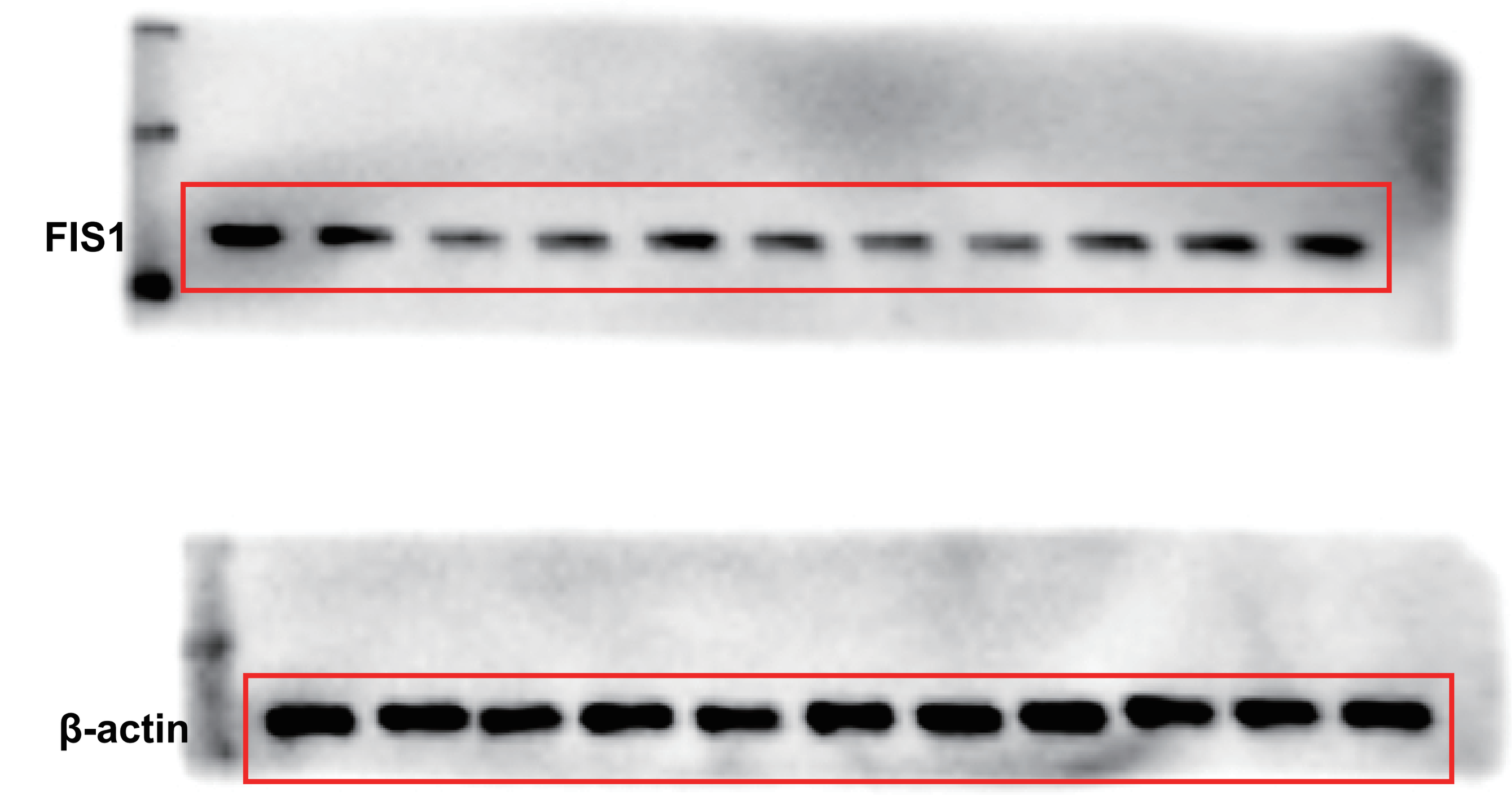

Fig1A

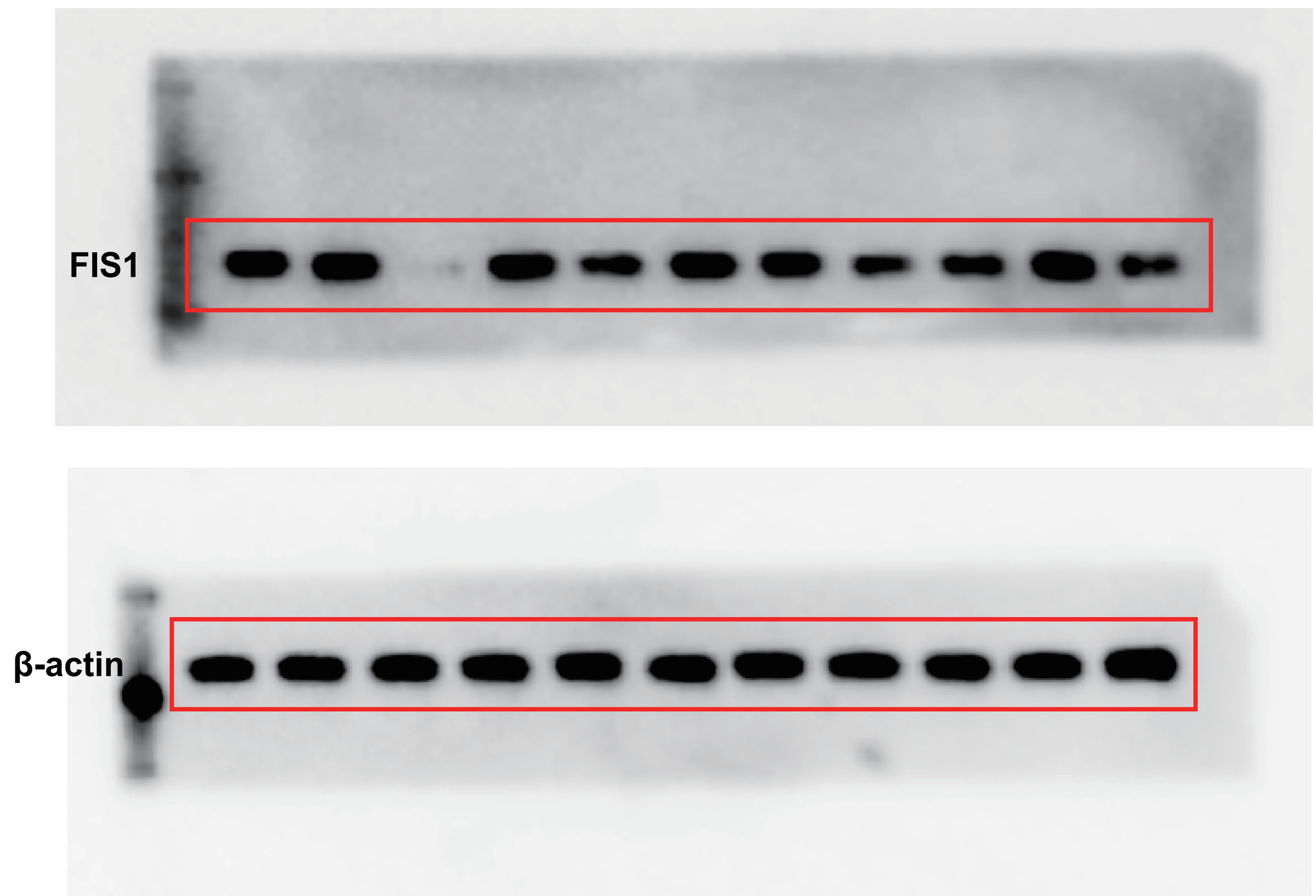

Fig1A

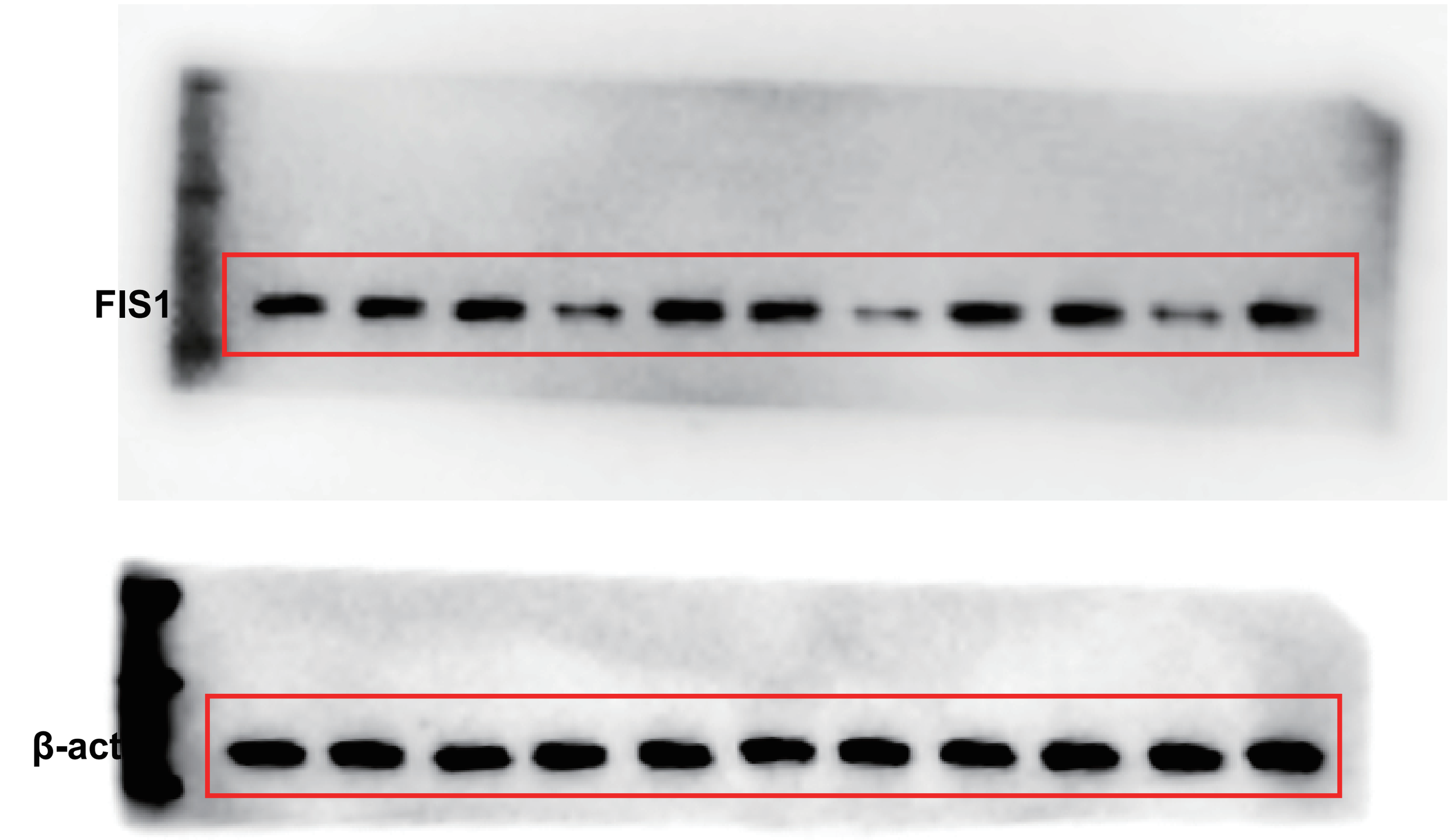

Fig1B

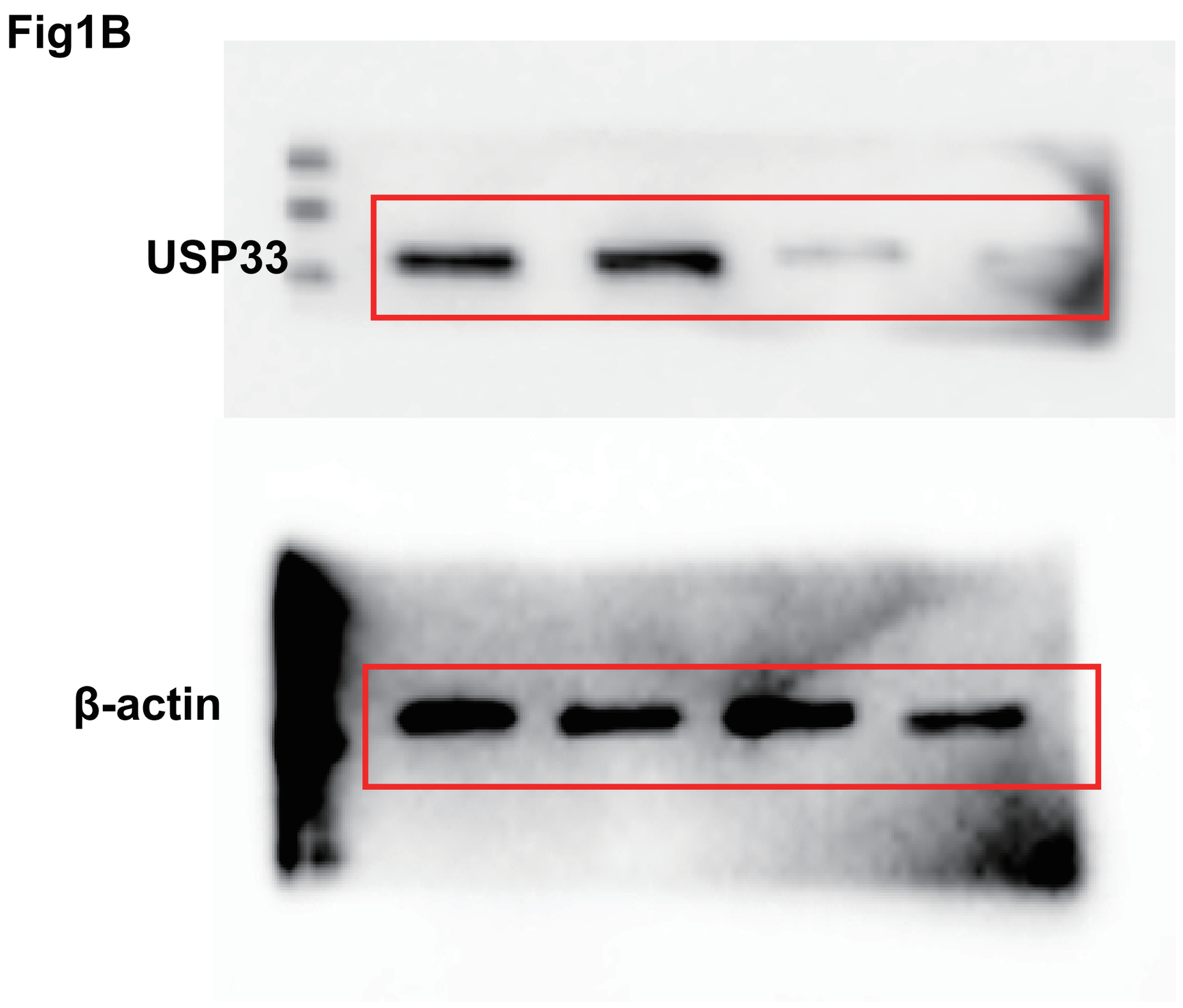

Fig1B

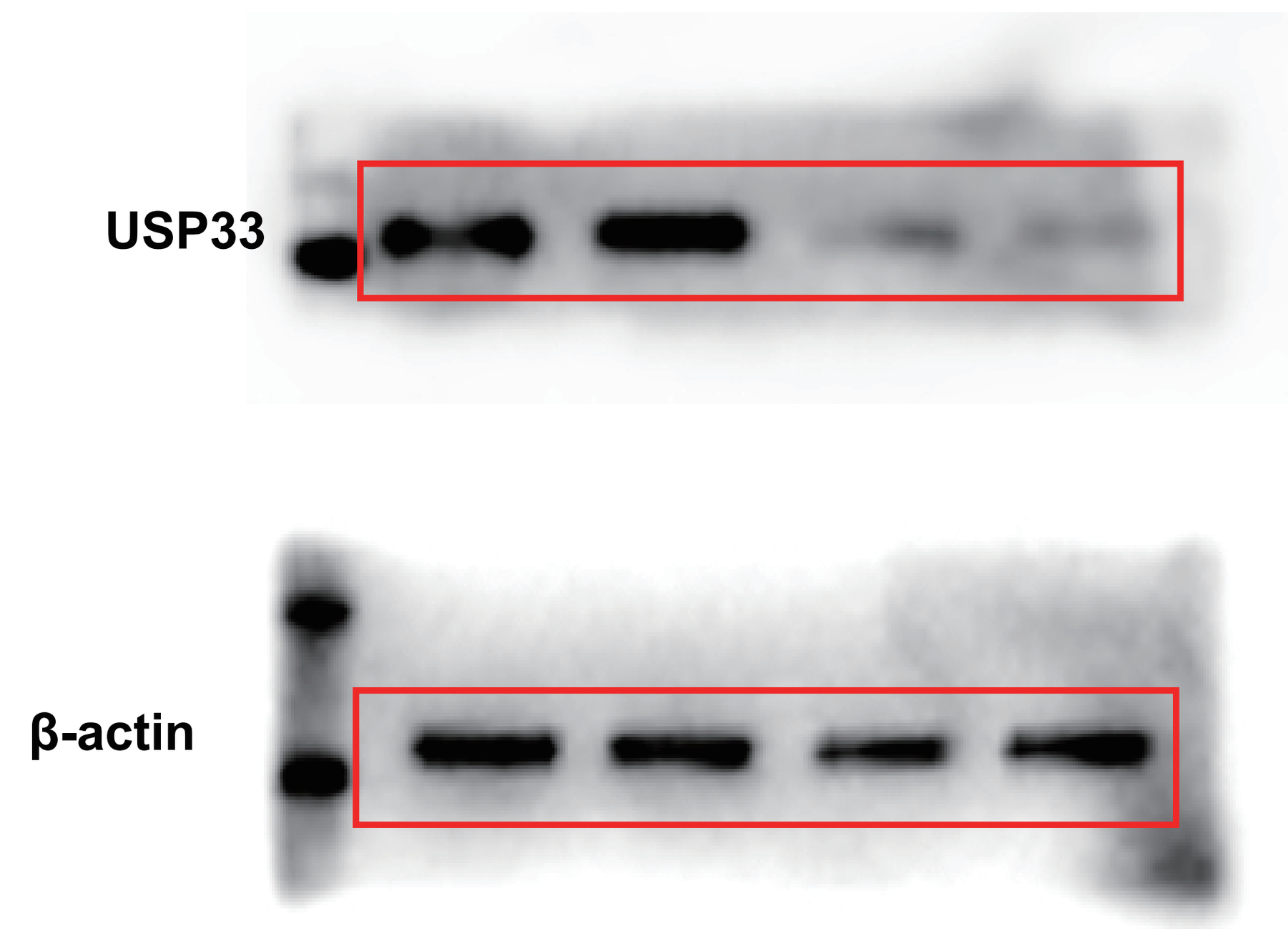

Fig1B

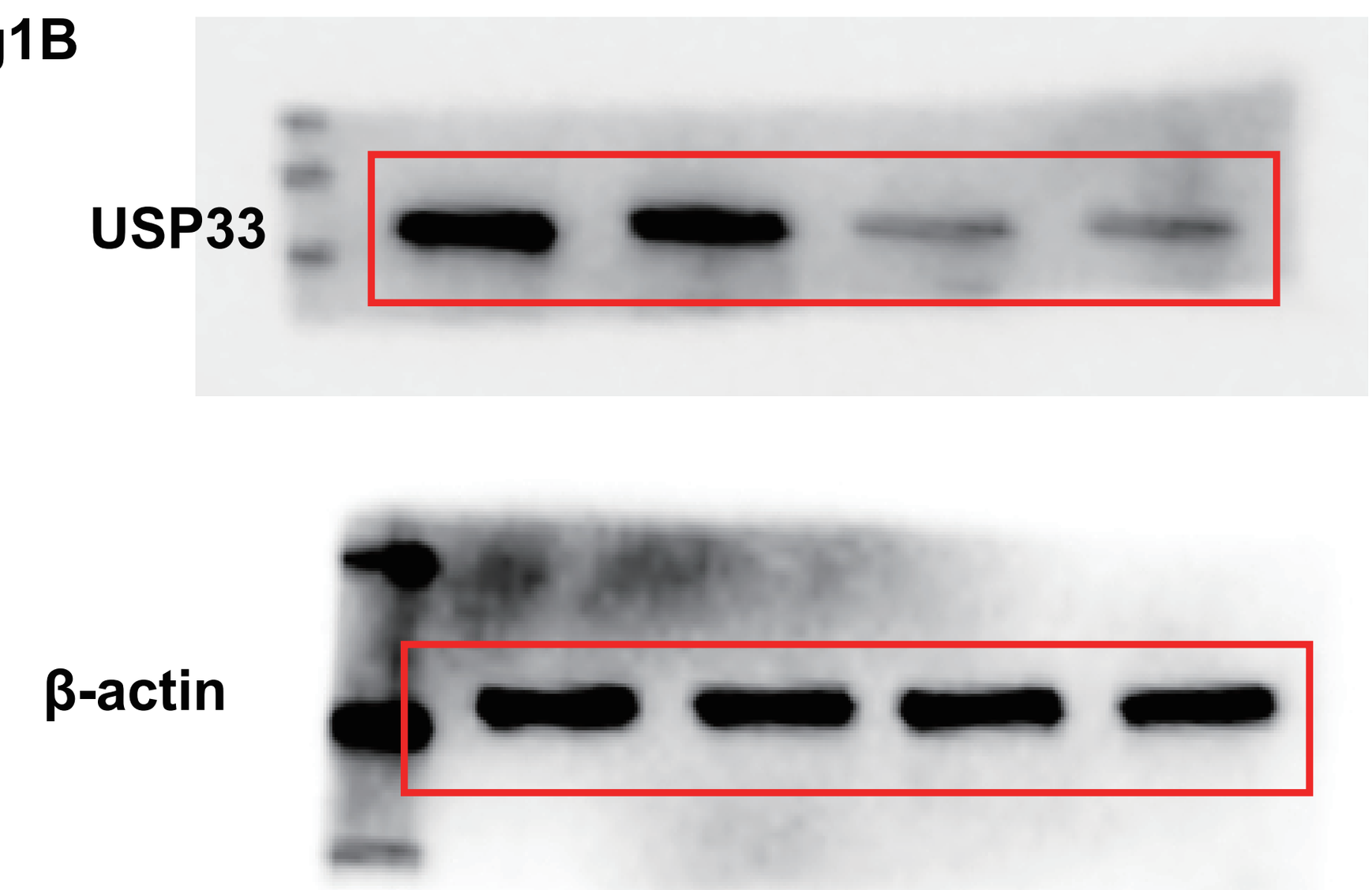

Fig1B

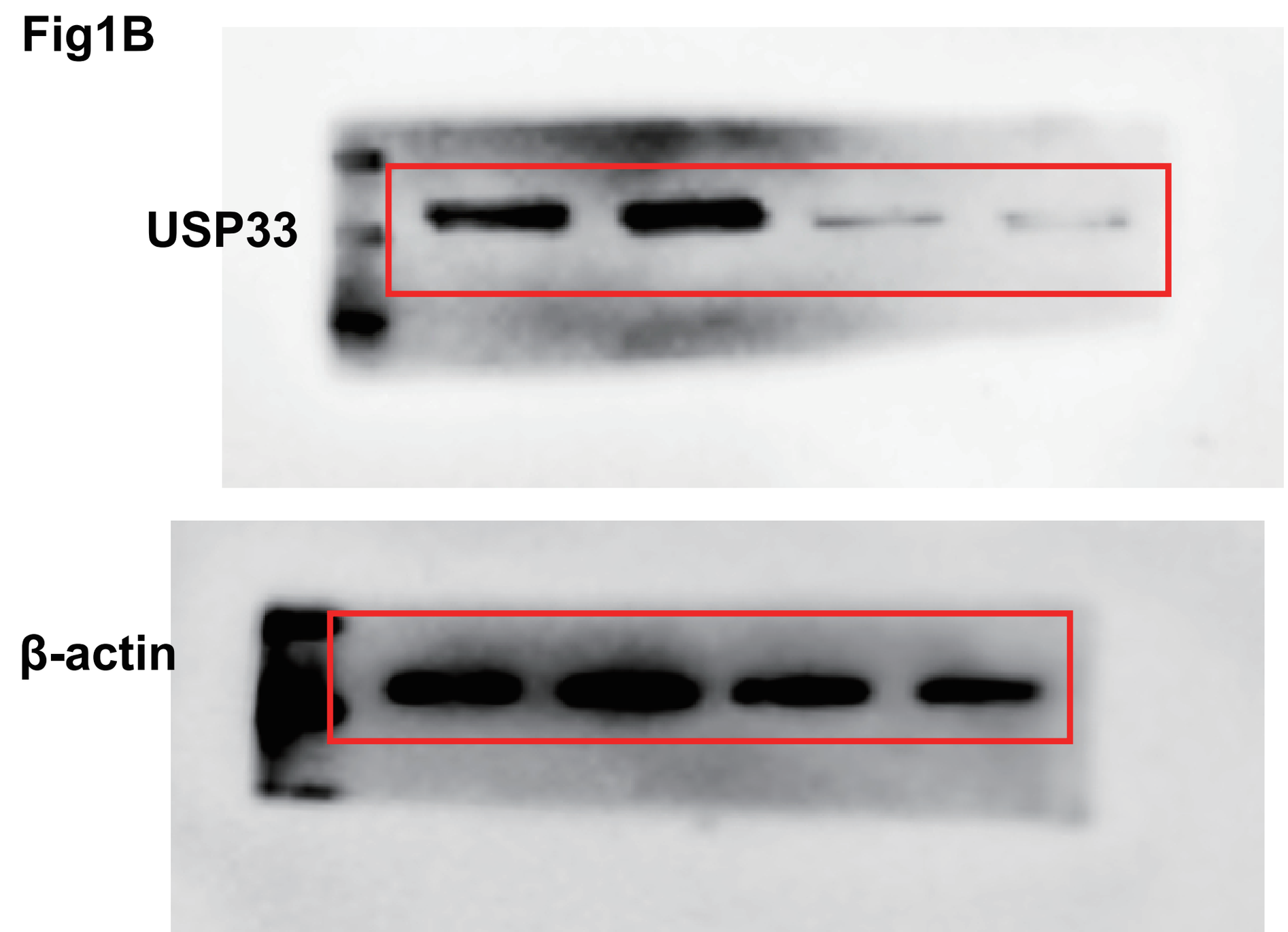

Fig1D

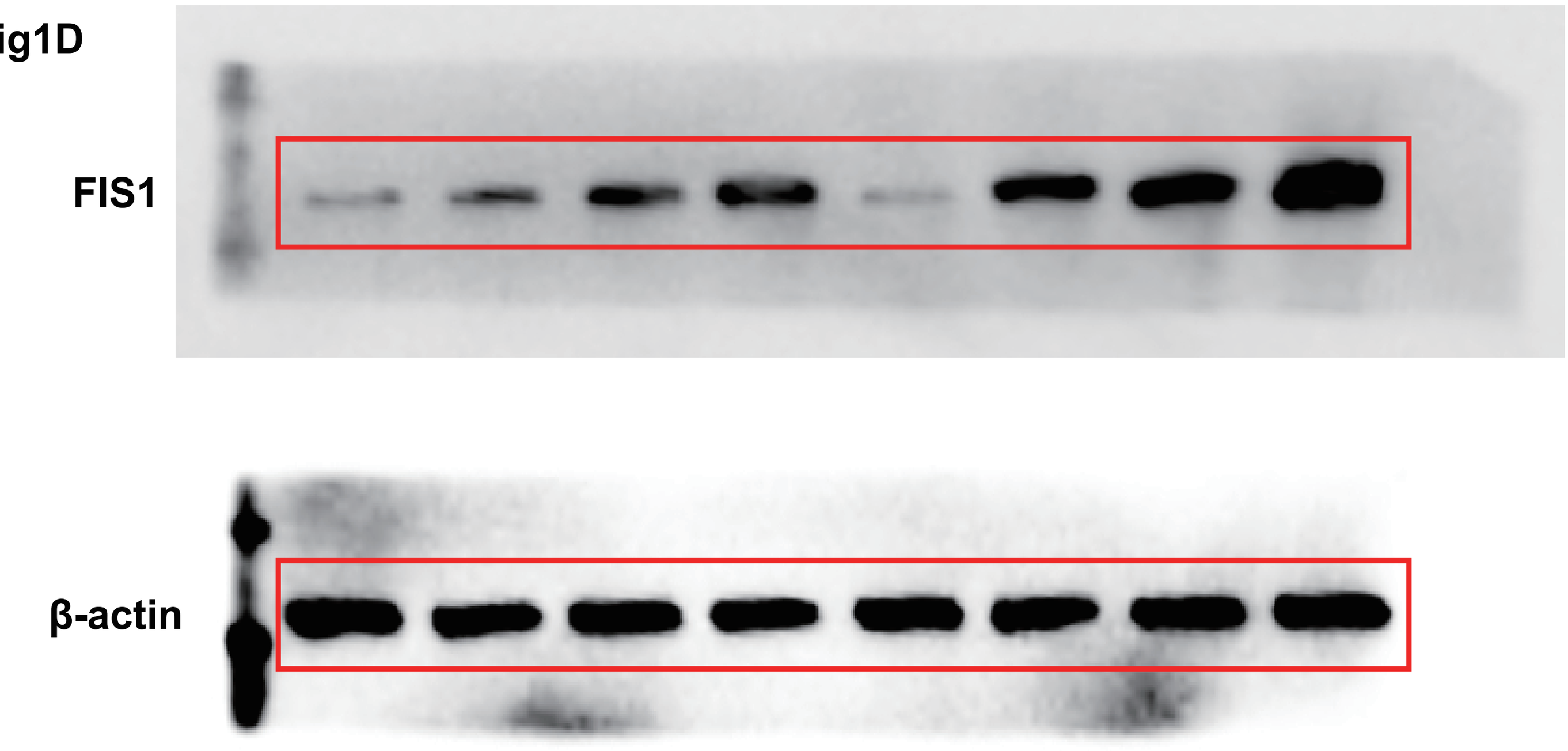

Fig1D

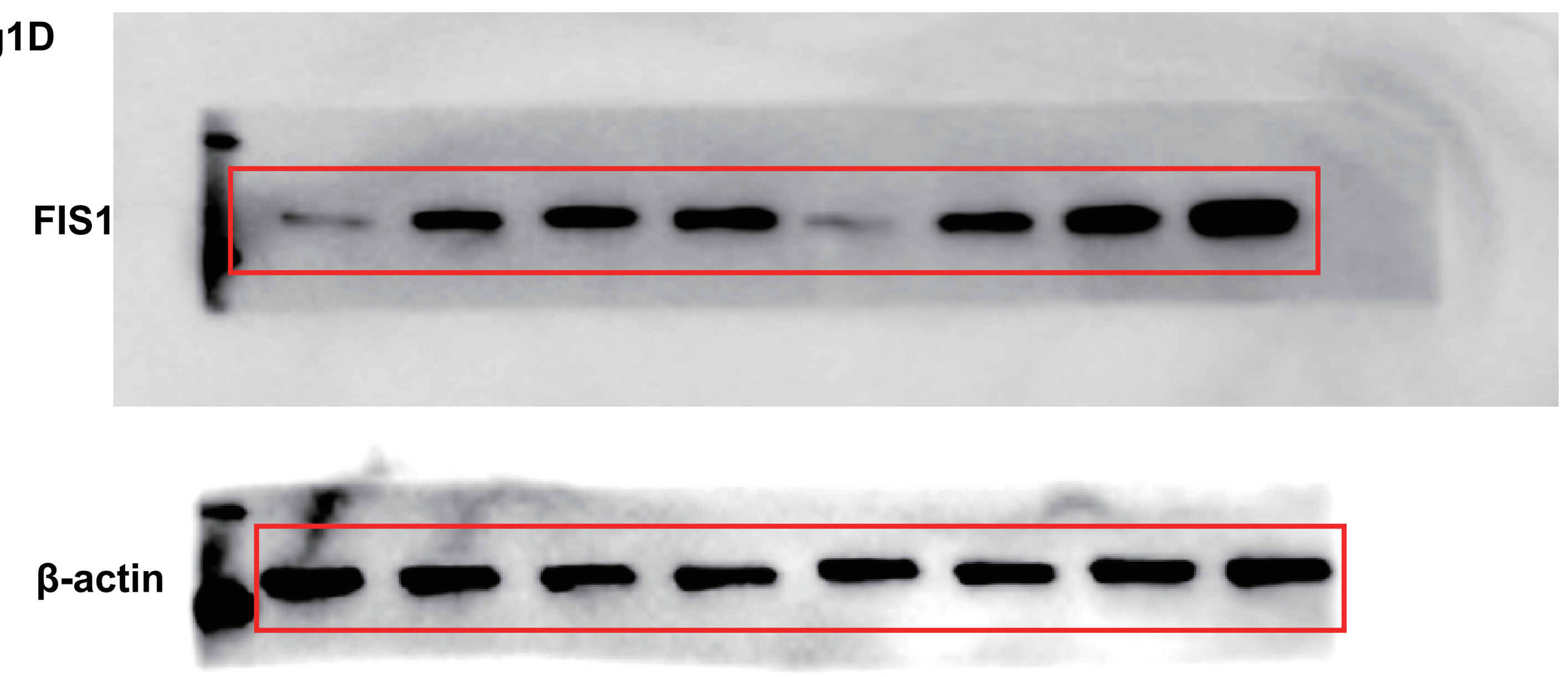

Fig1F

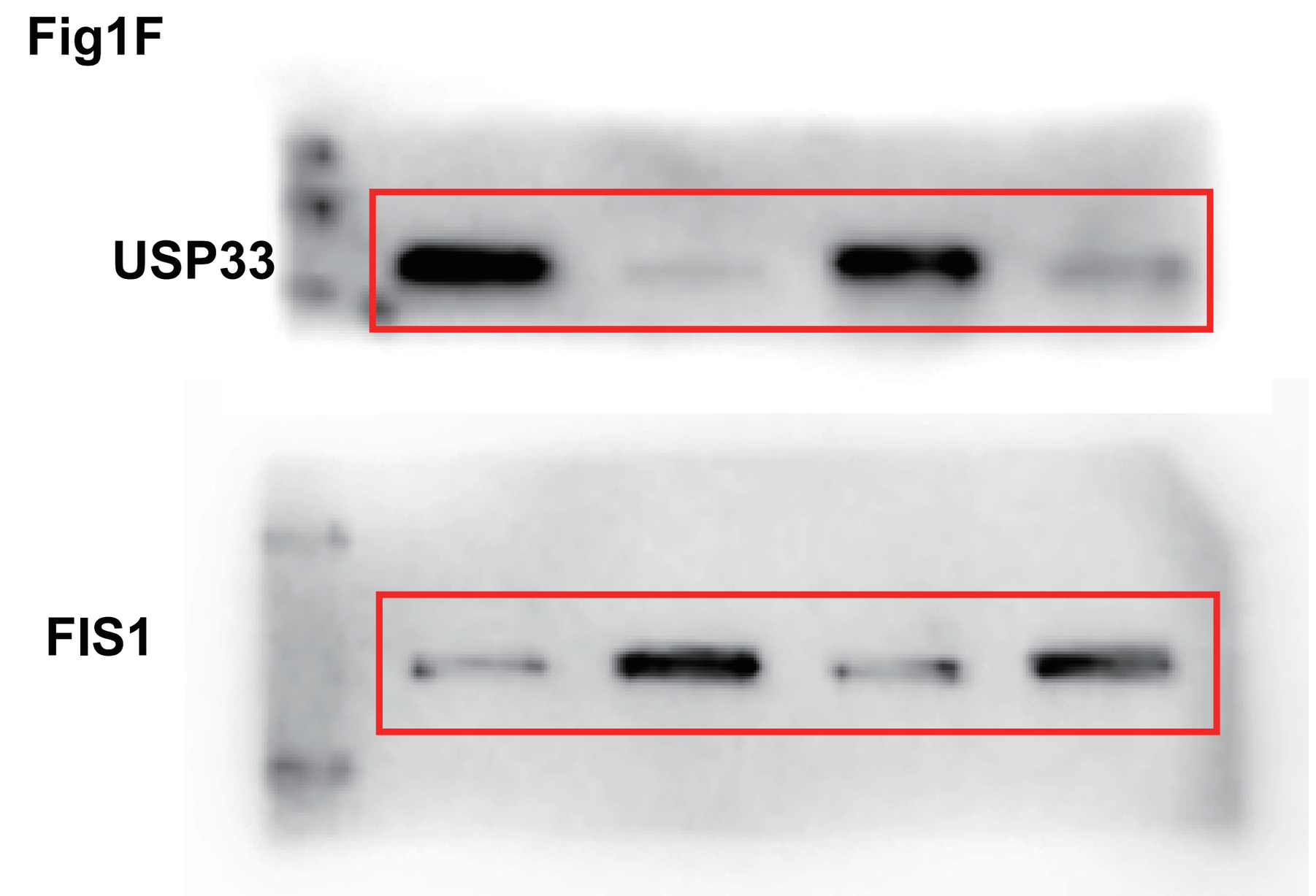

Fig1F

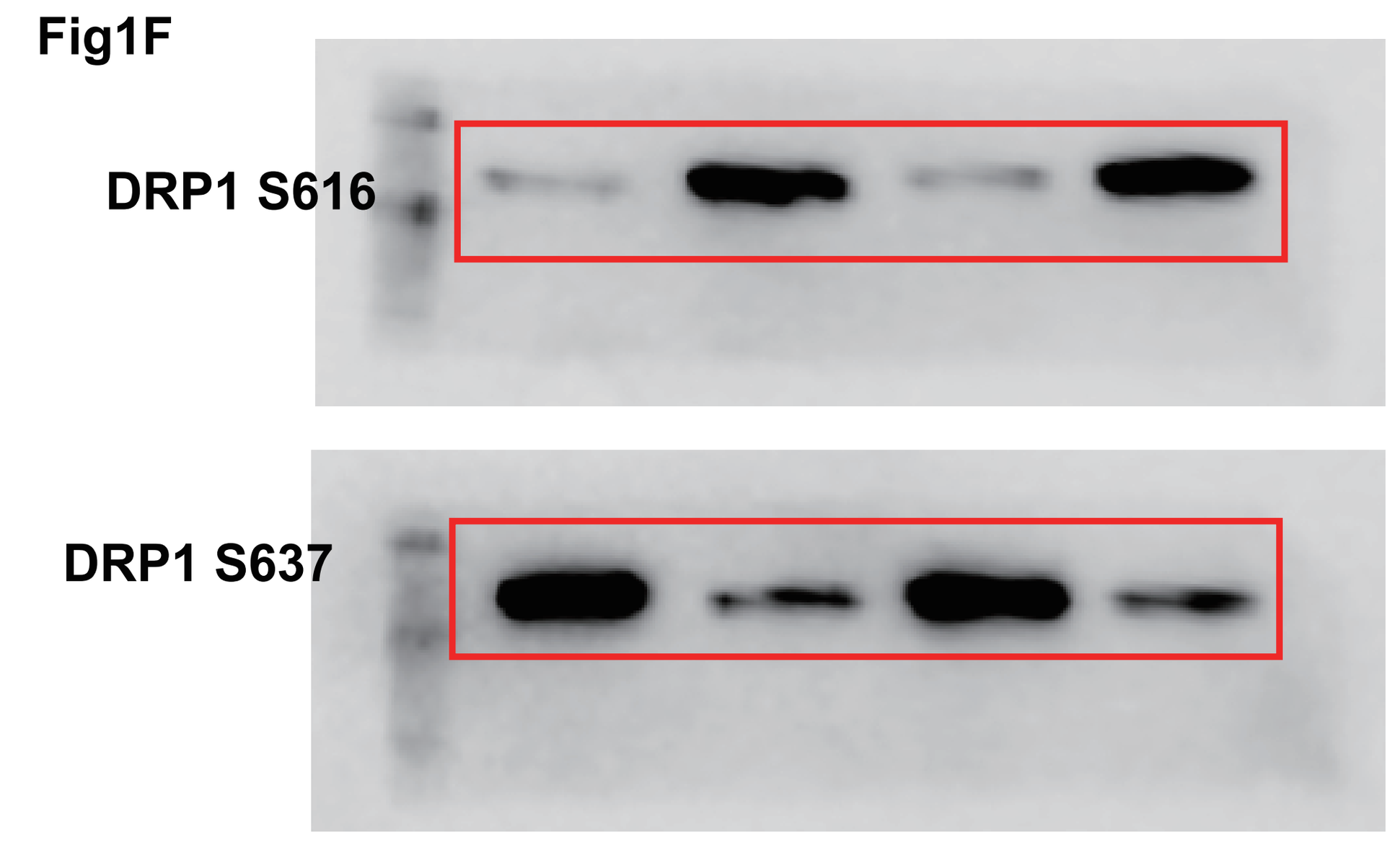

Fig1F

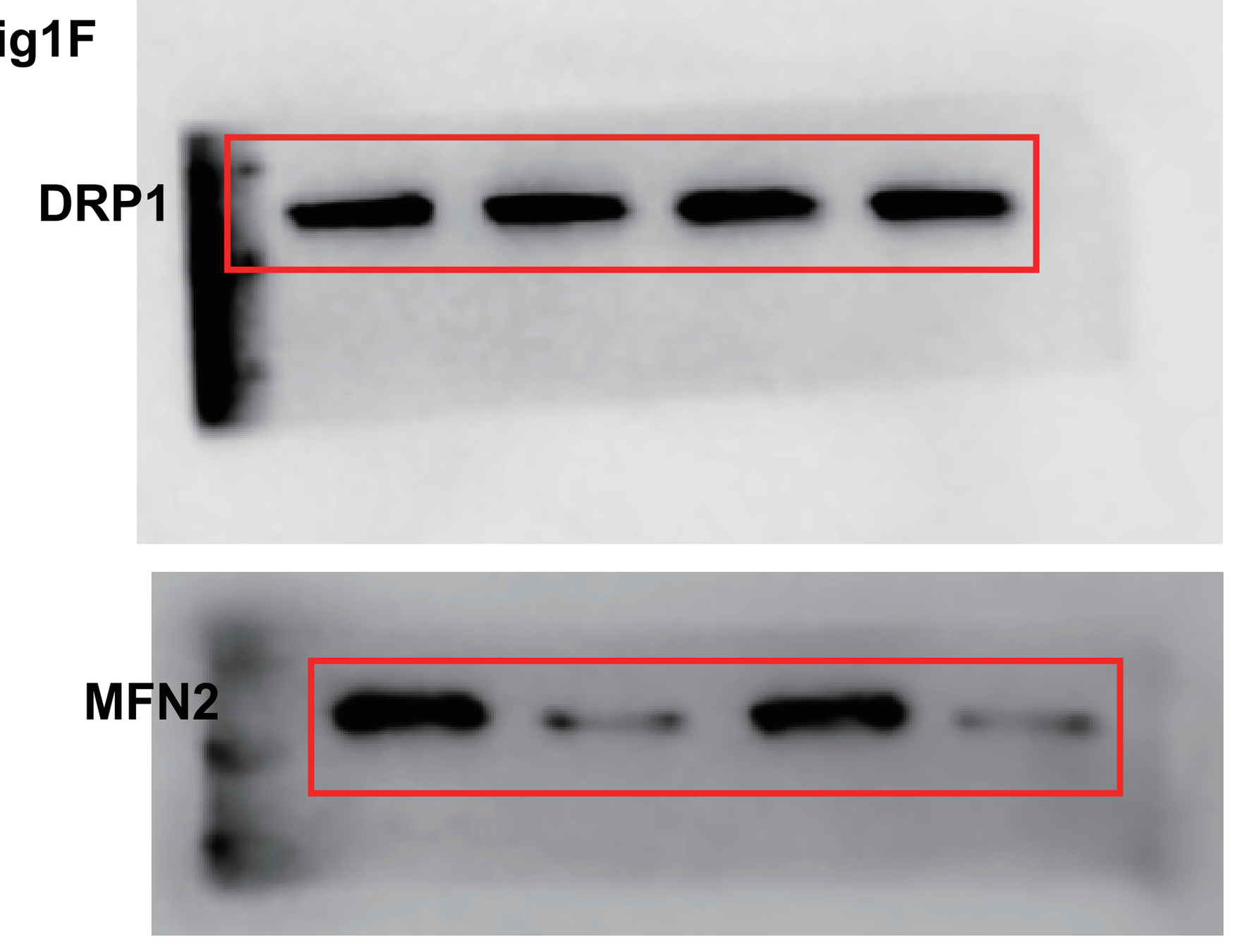

Fig1F

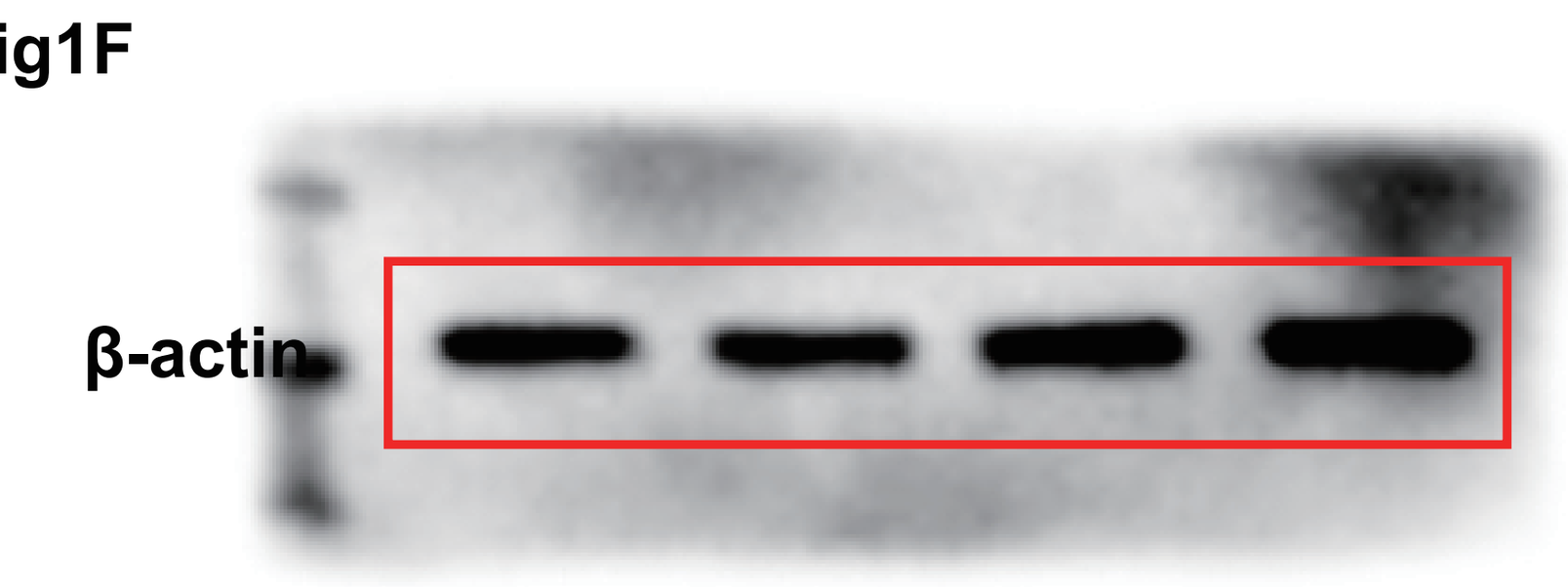

Fig1H

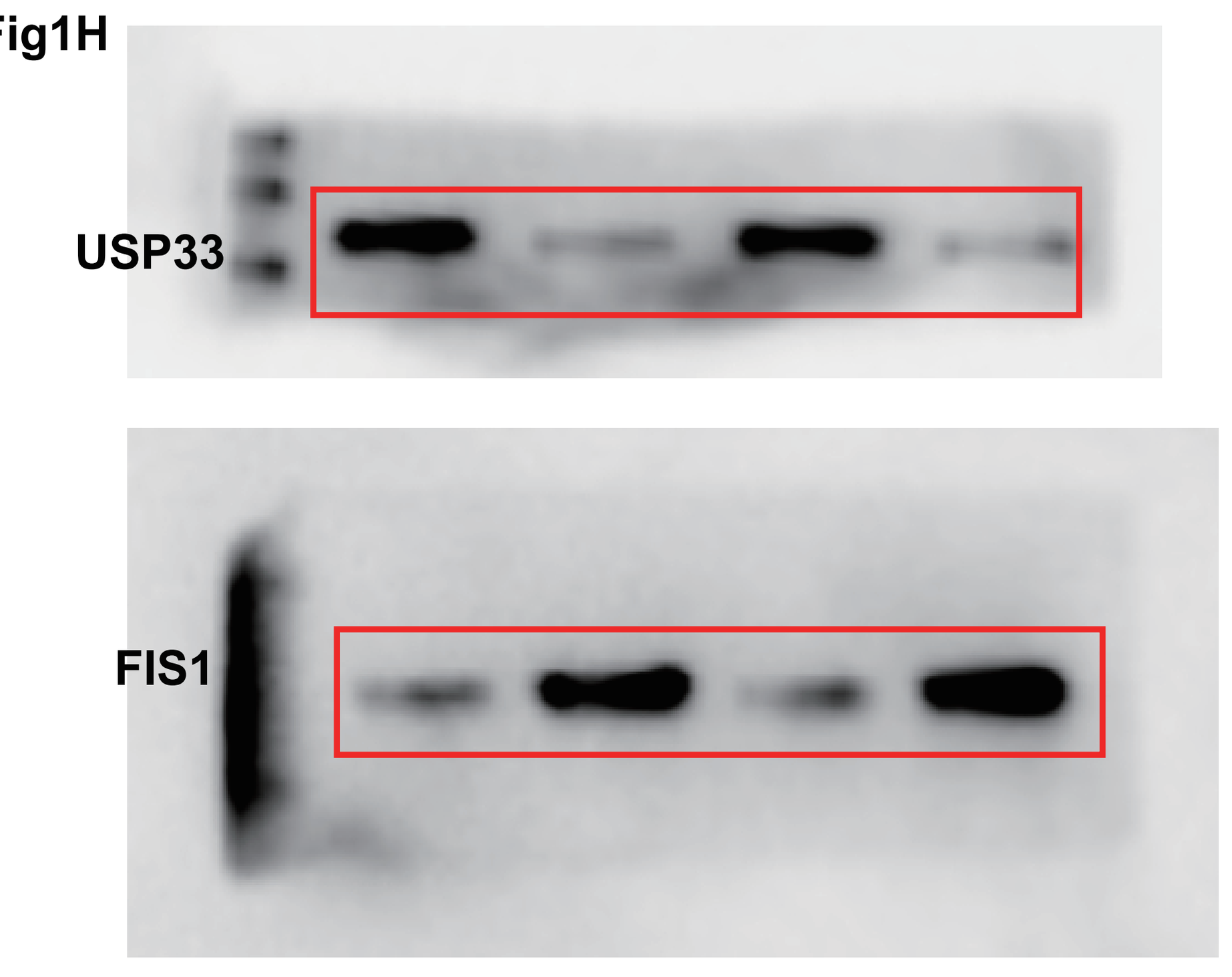

Fig1H

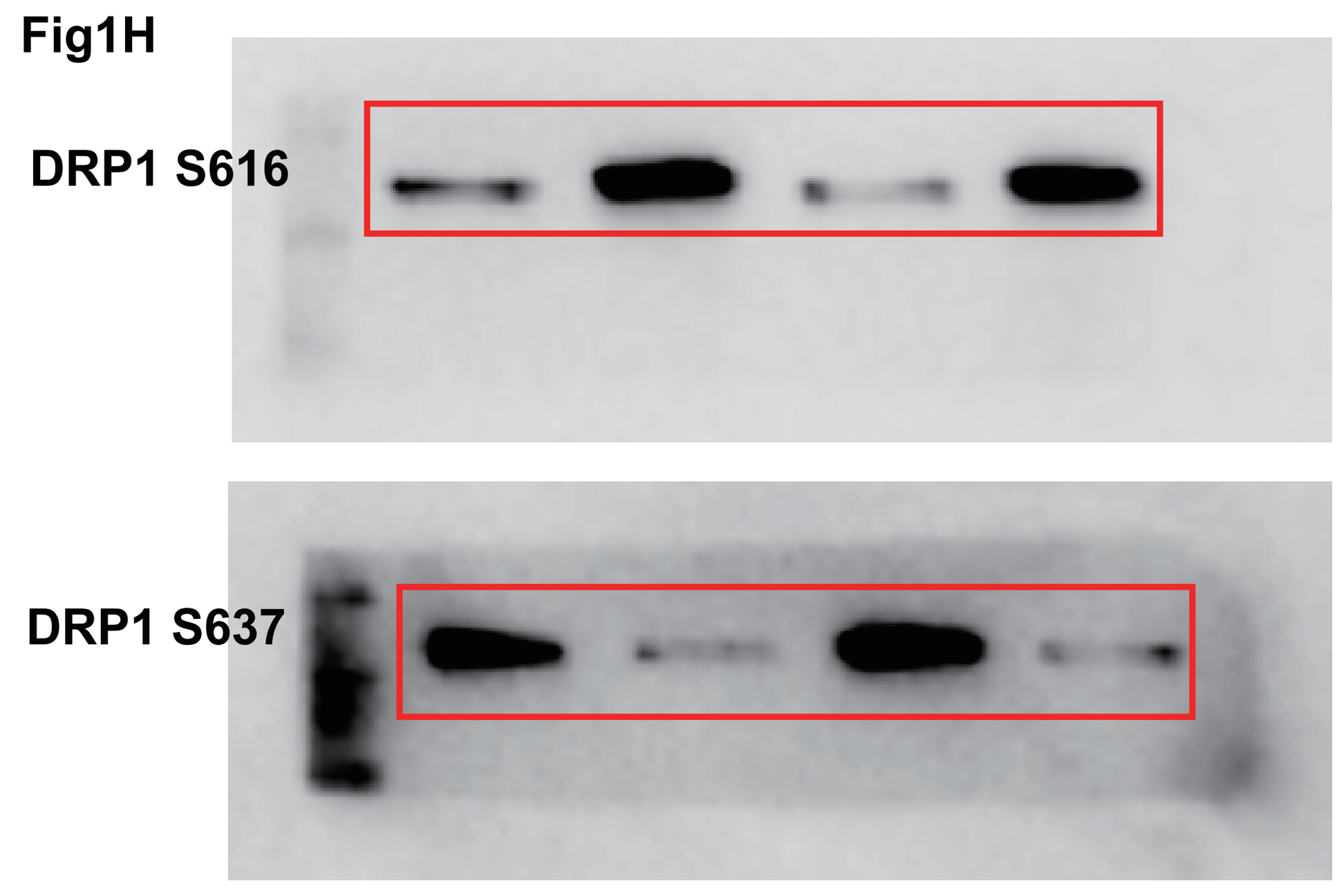

Fig1H

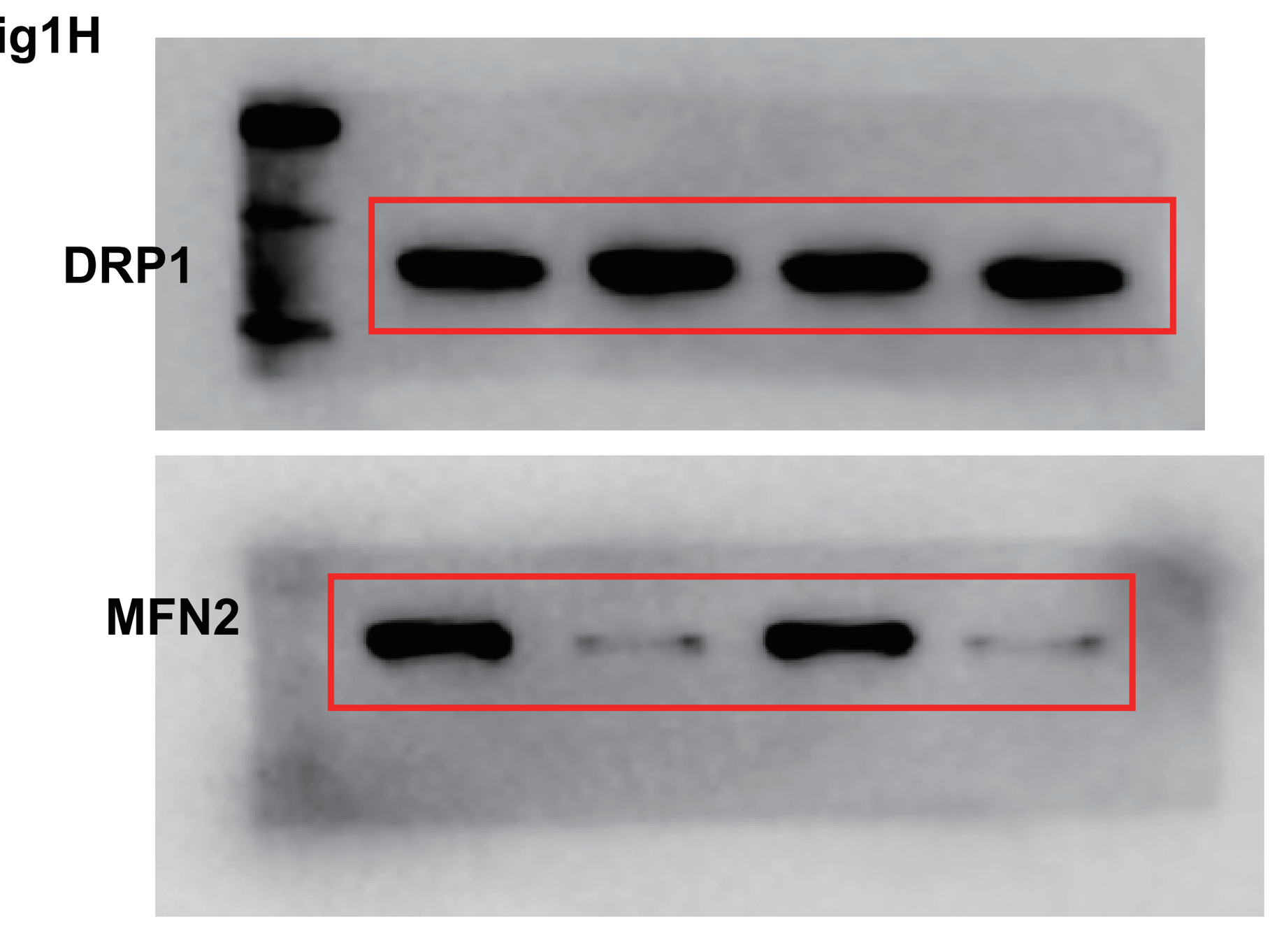

Fig1H

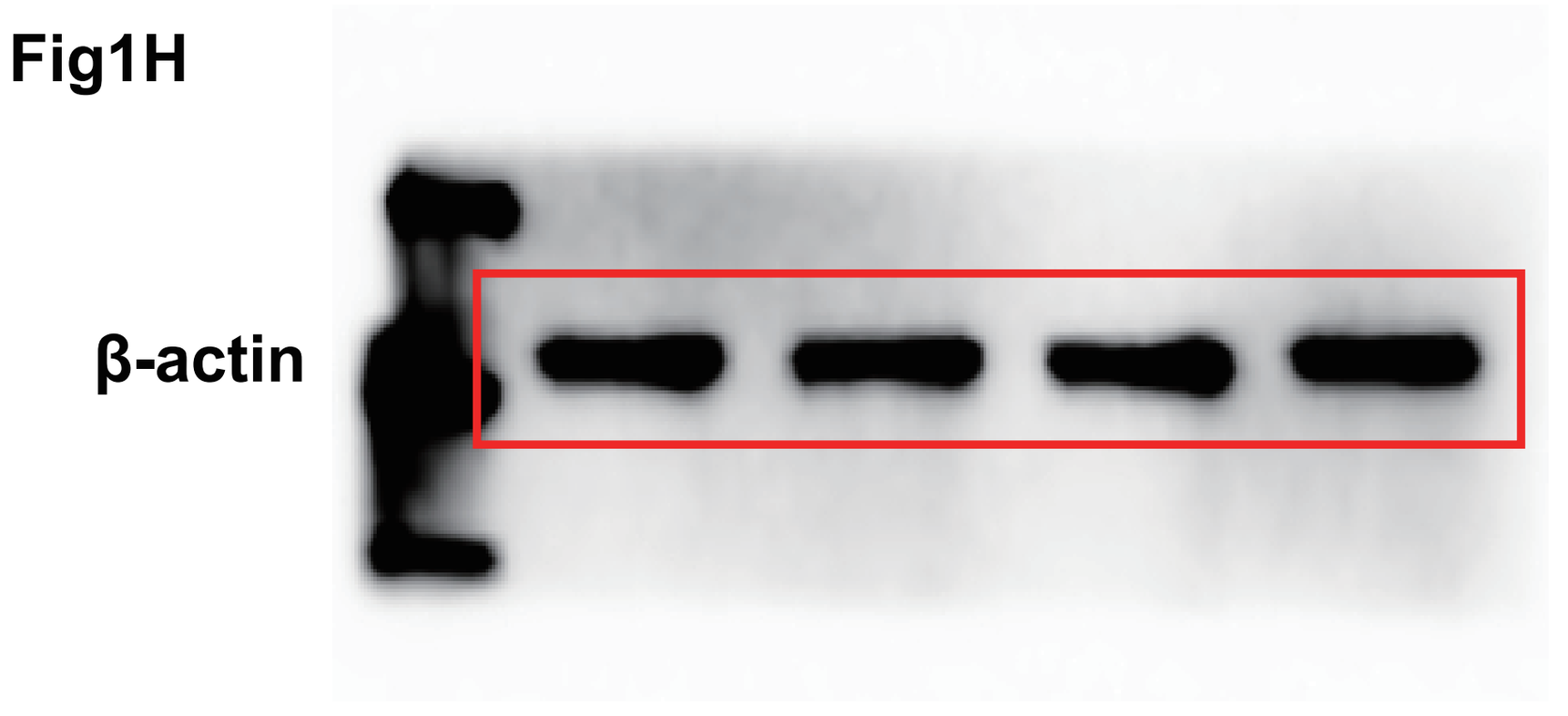

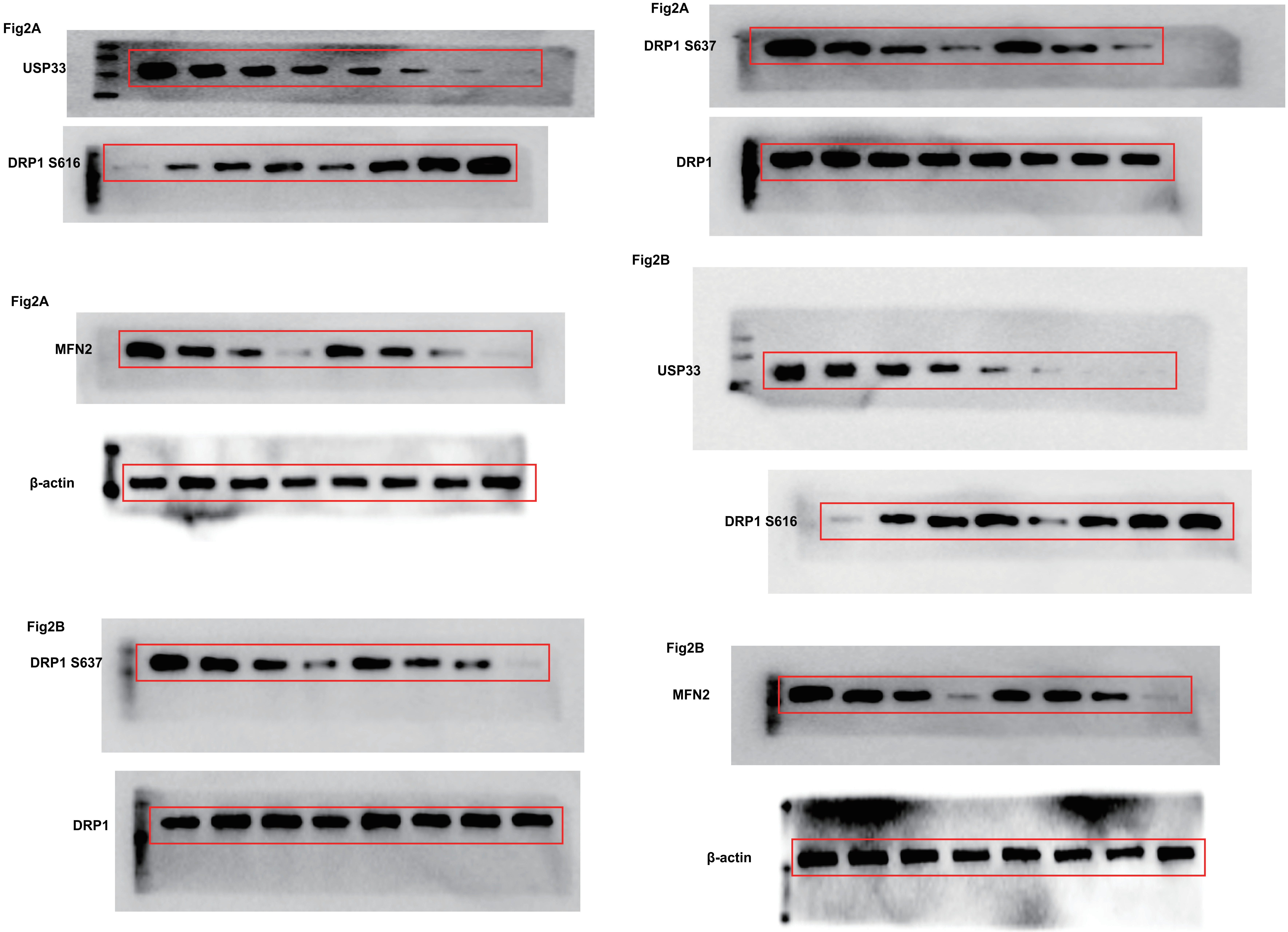

Fig2A
USP33
DRP1 S616
Fig2A
MFN2
β-actin
Fig2B
DRP1 S637
DRP1
Fig2A
DRP1 S637
DRP1
Fig2B
USP33
DRP1 S616
Fig2B
MFN2
β-actin

Fig3G

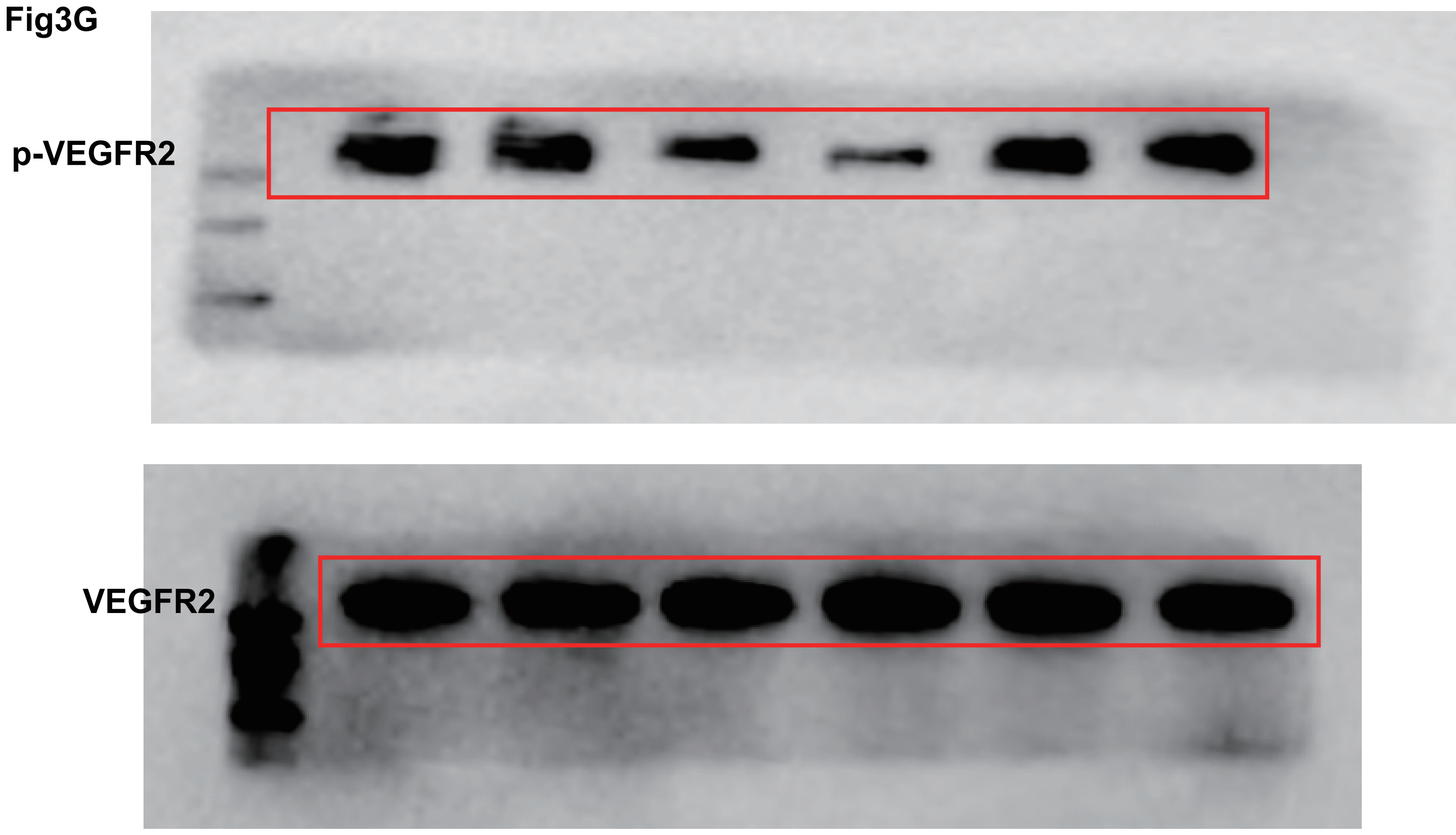

Fig3G

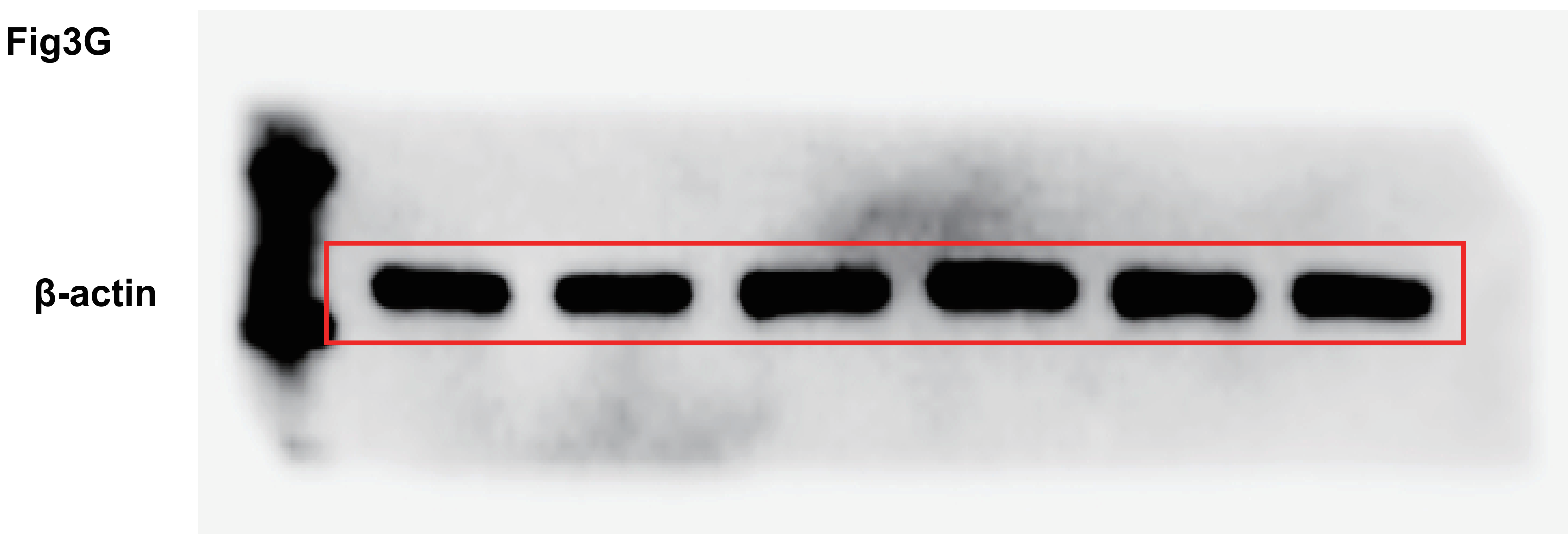

Fig3I

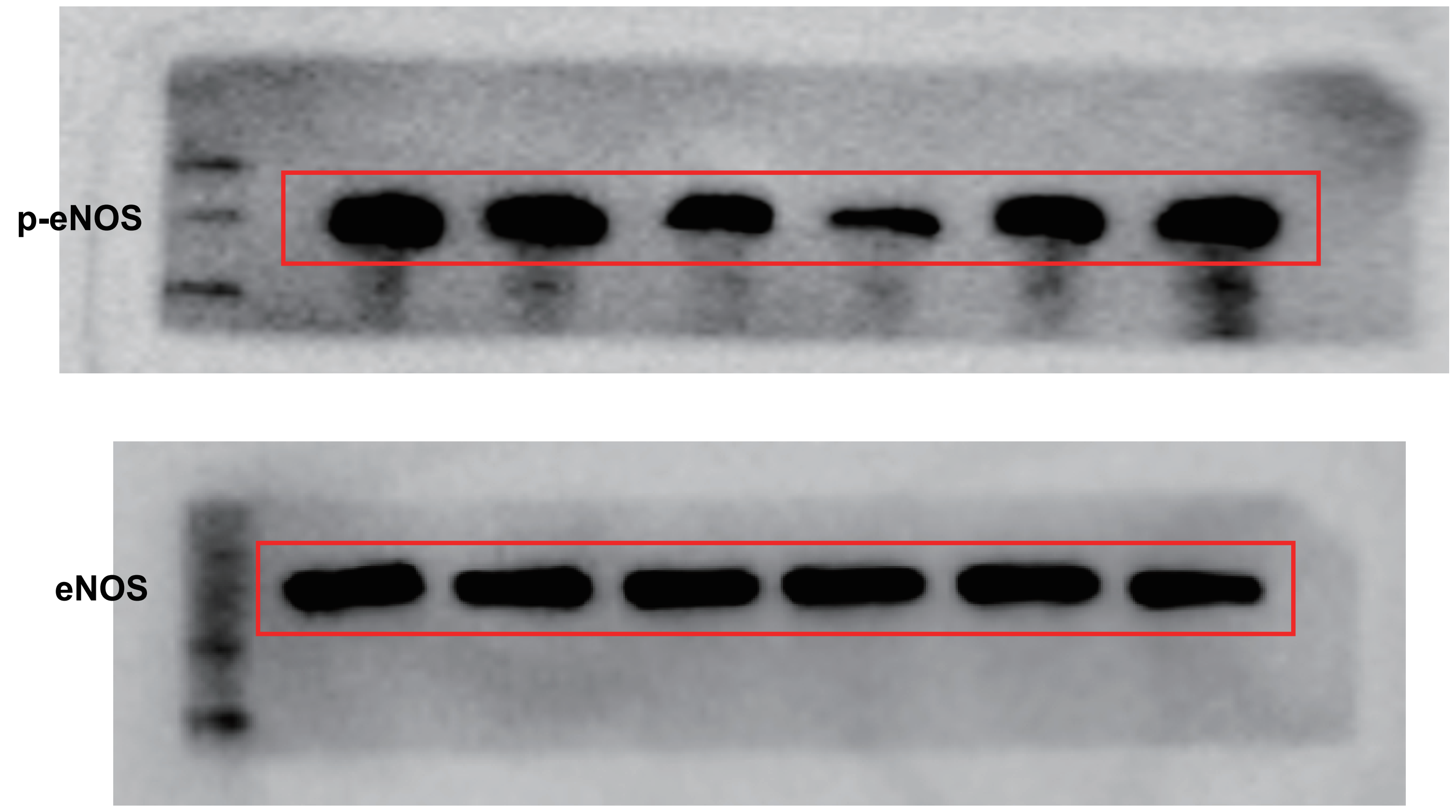

Fig3I

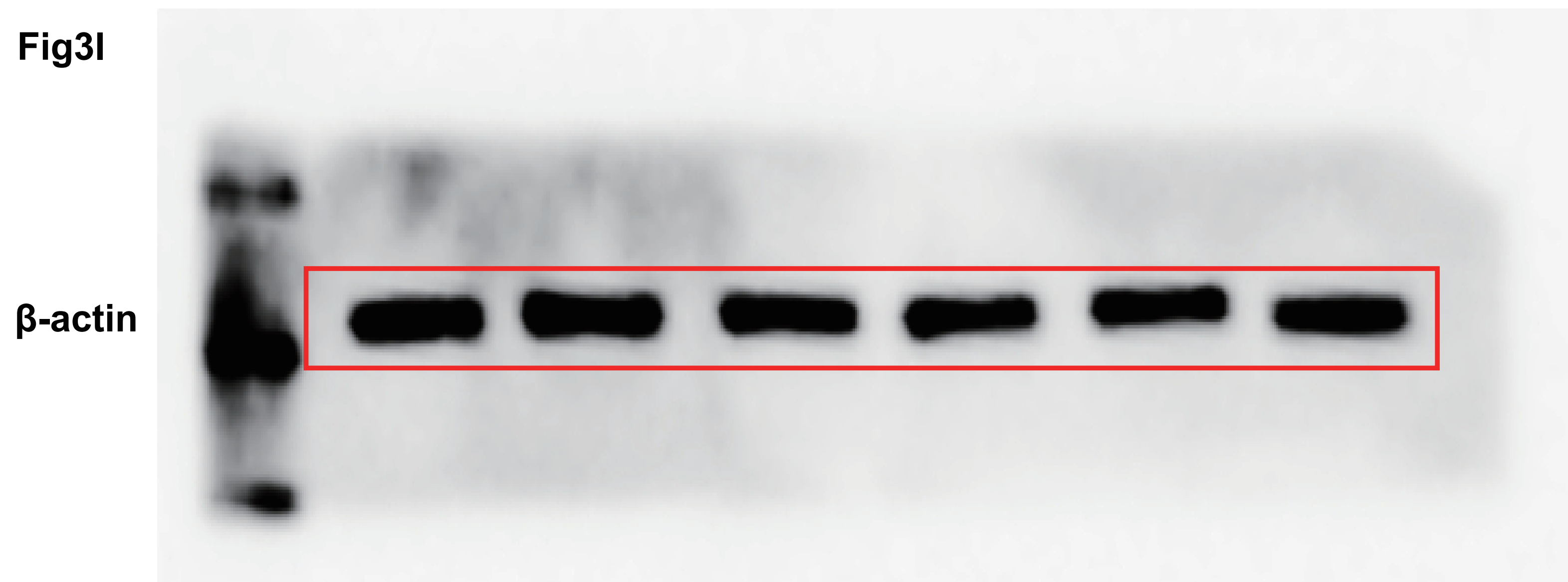

Fig3K

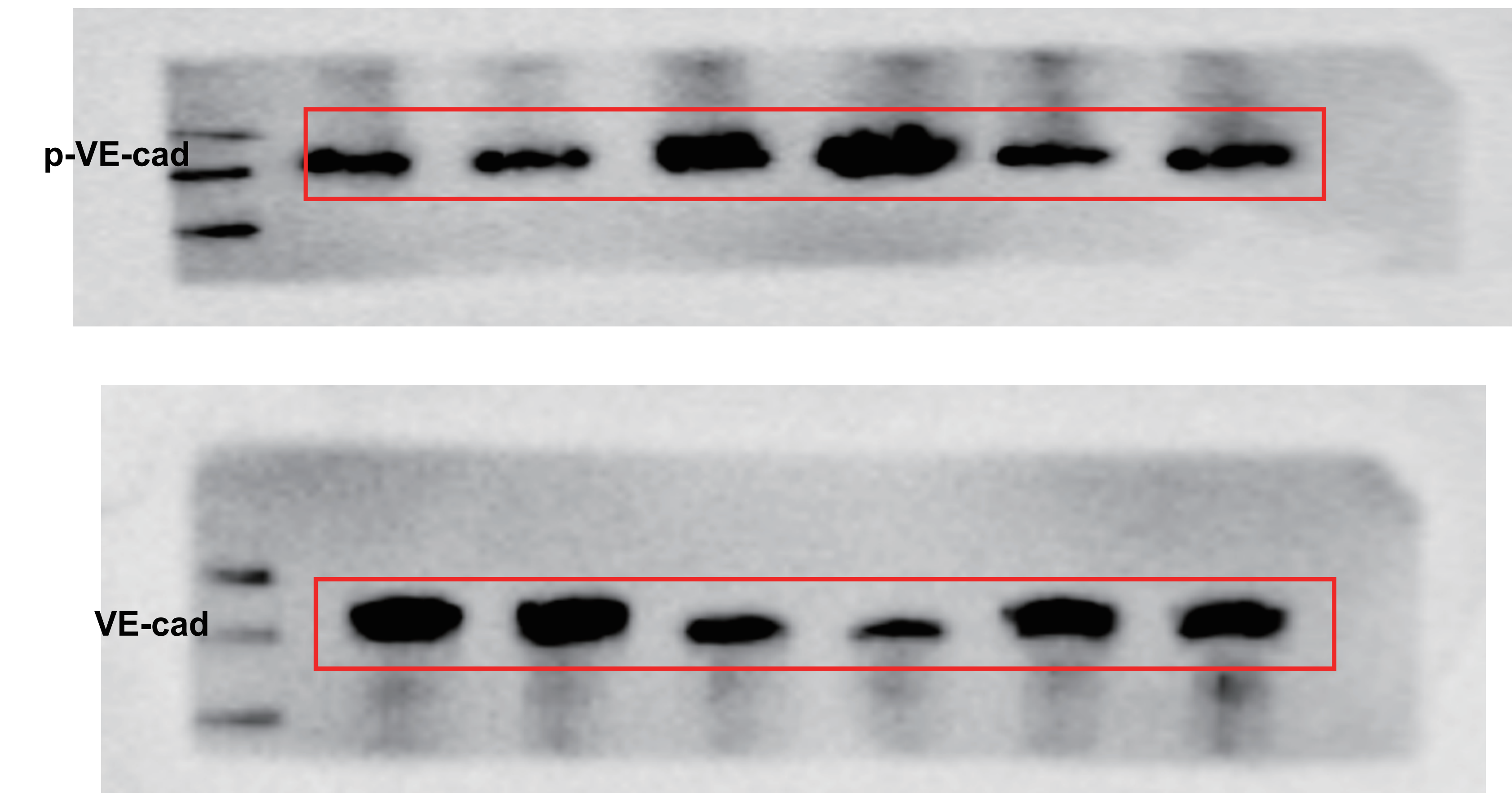

Fig3K

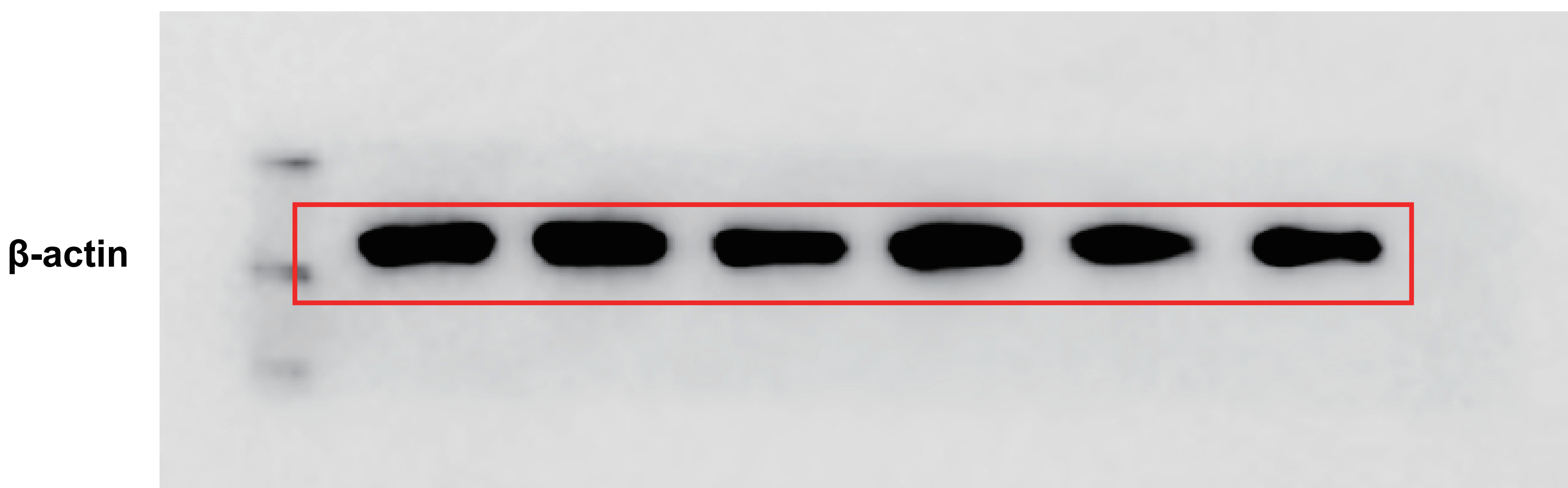

Fig4C

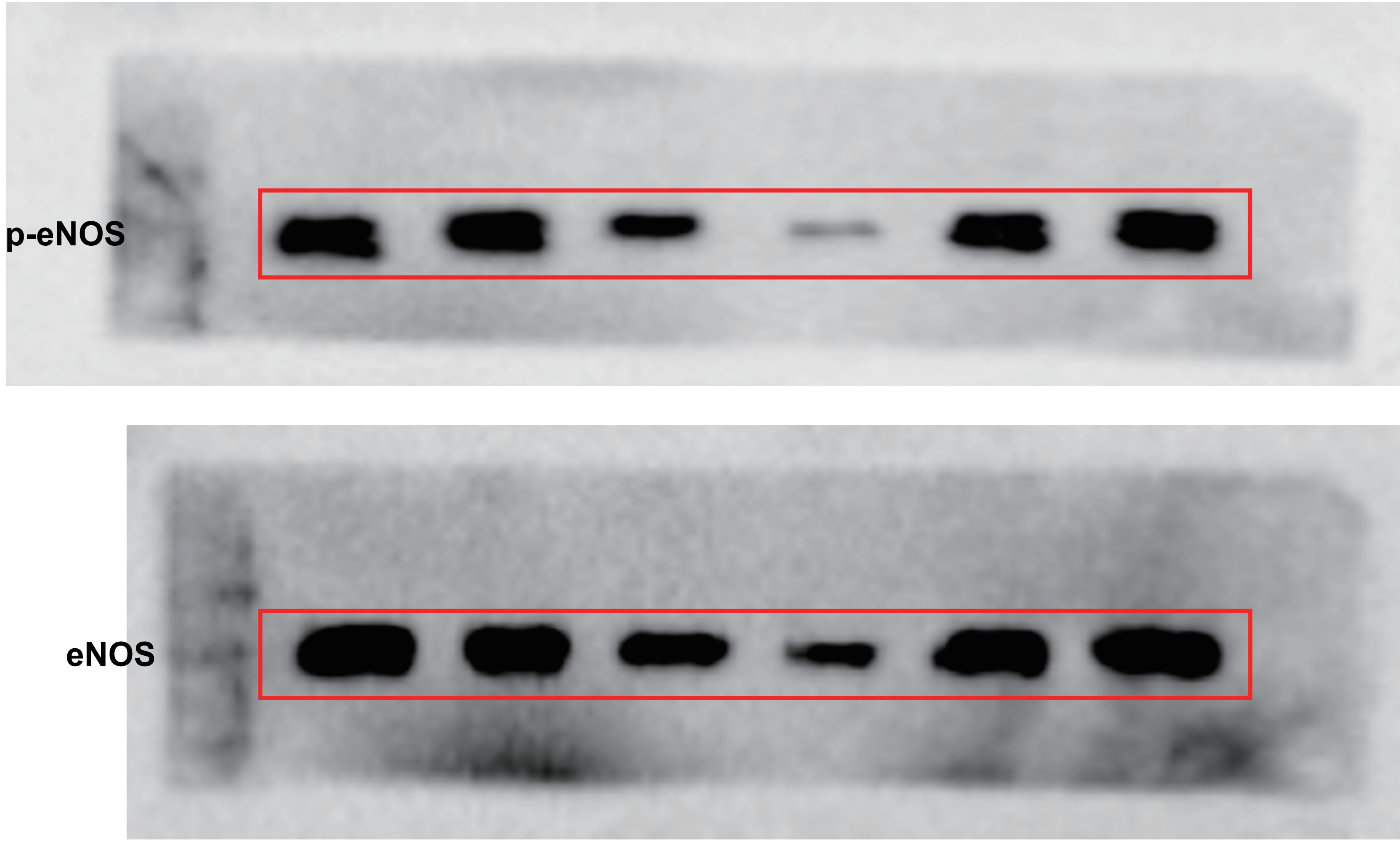

Fig4C

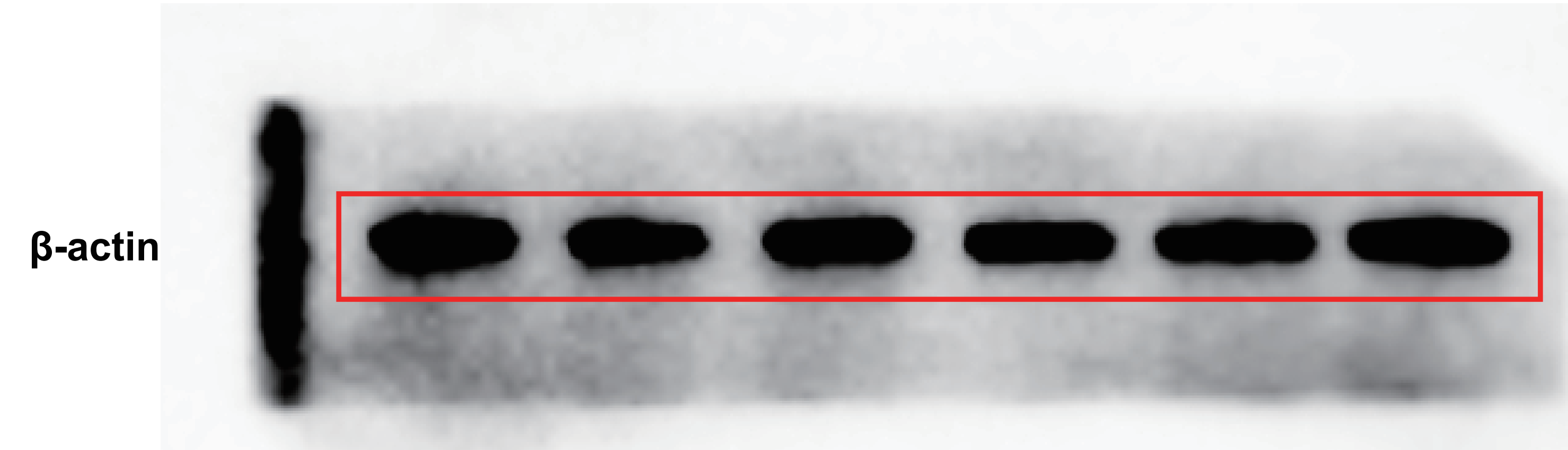

Fig4D

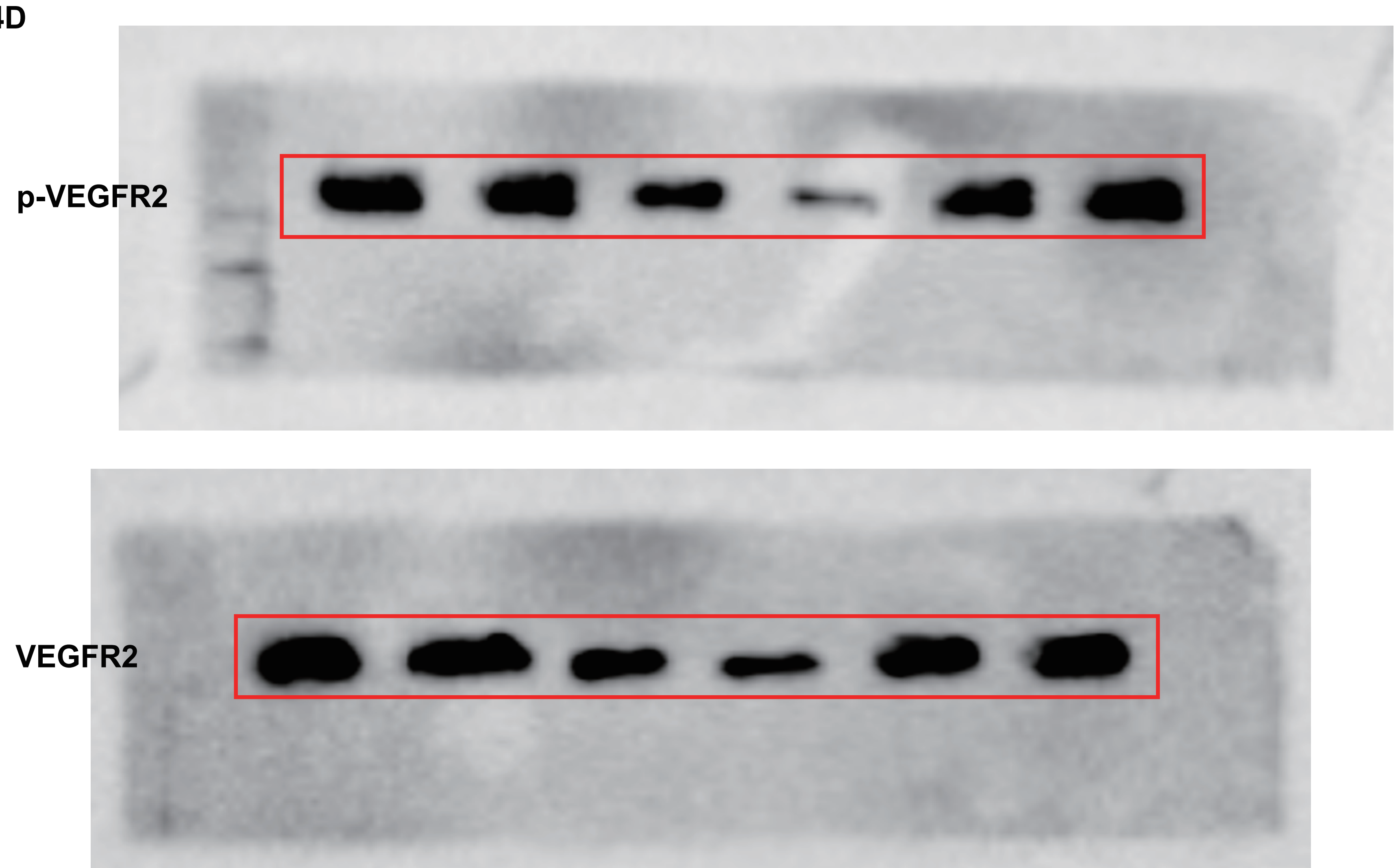

Fig4D

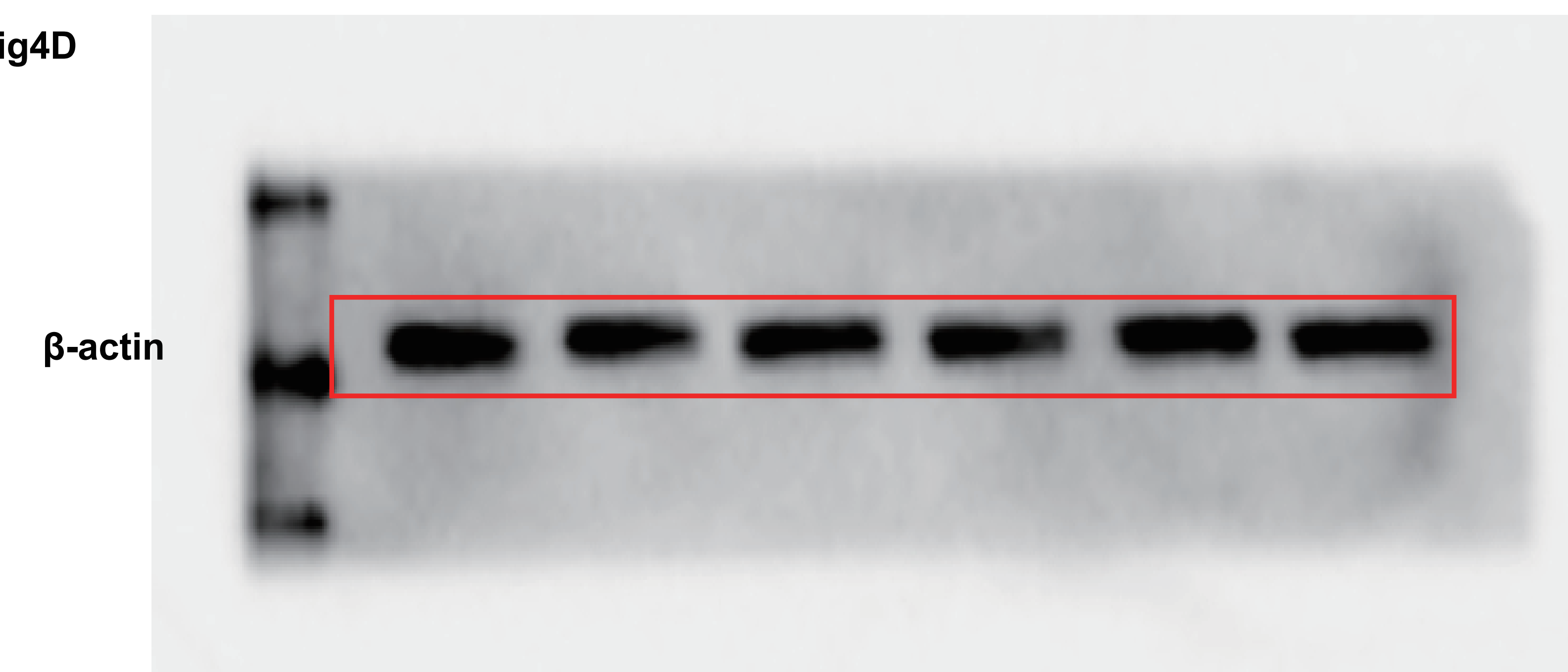

Fig4E

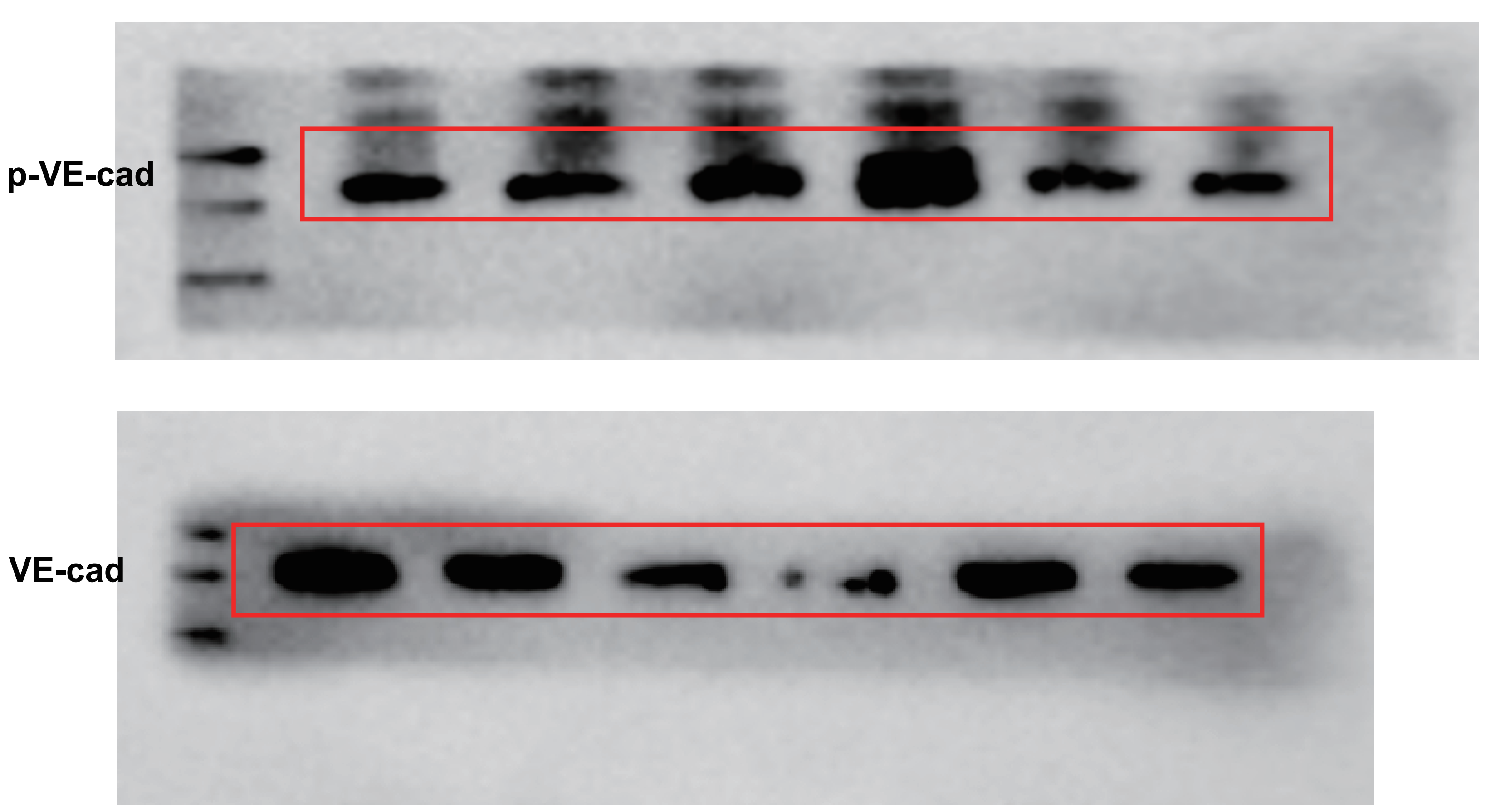

Fig4E

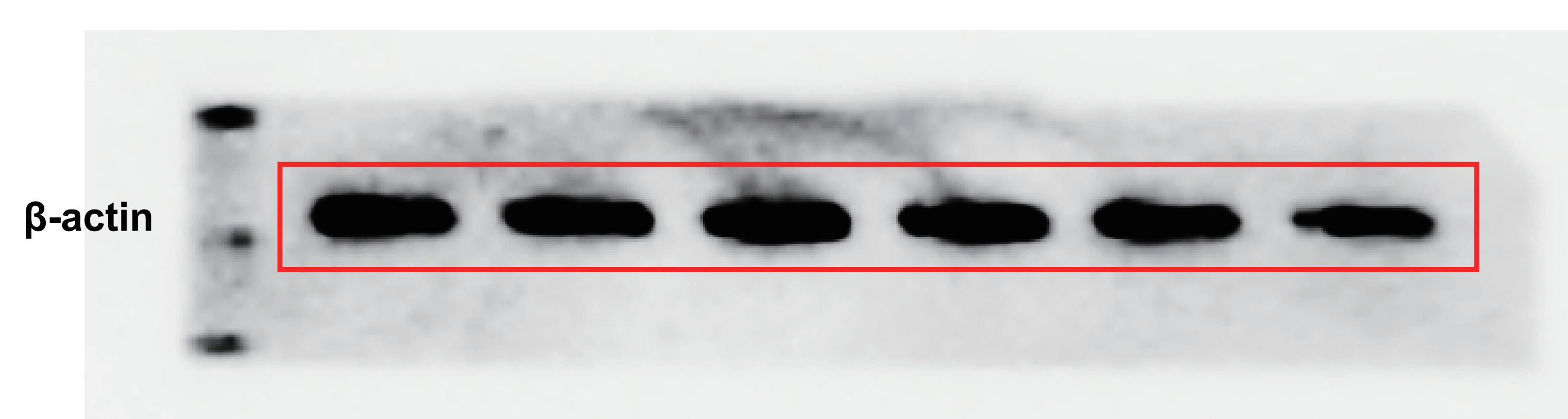

Fig5A

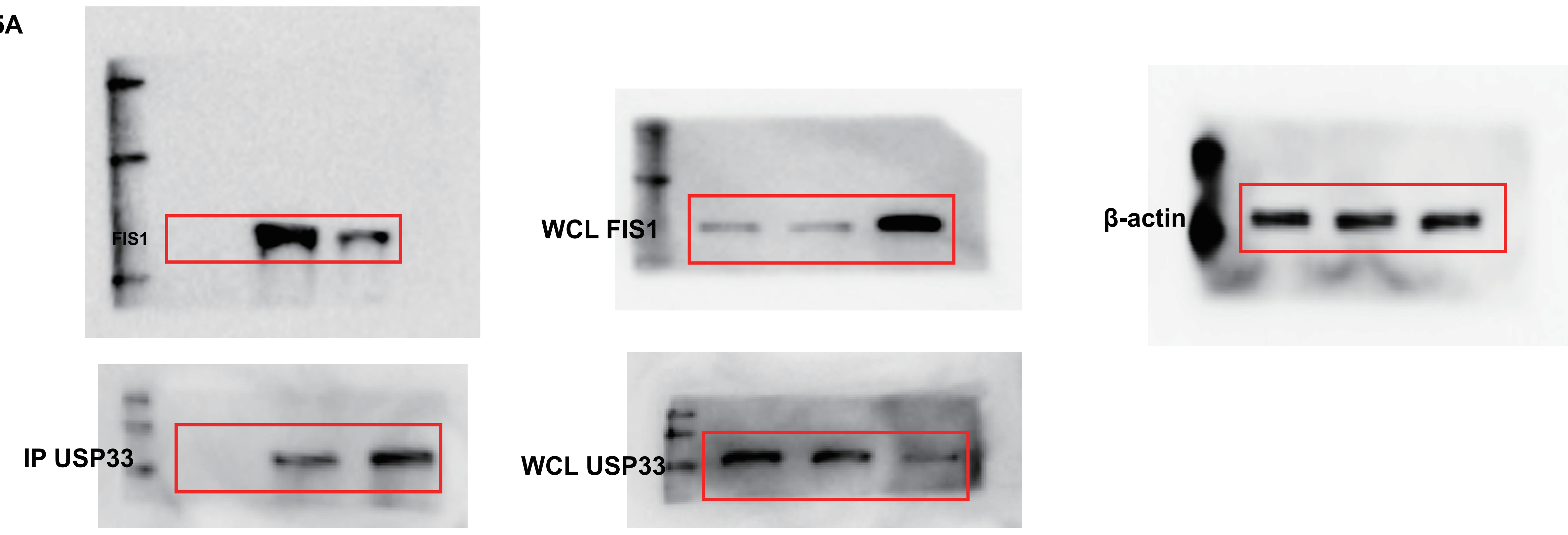

Fig5B

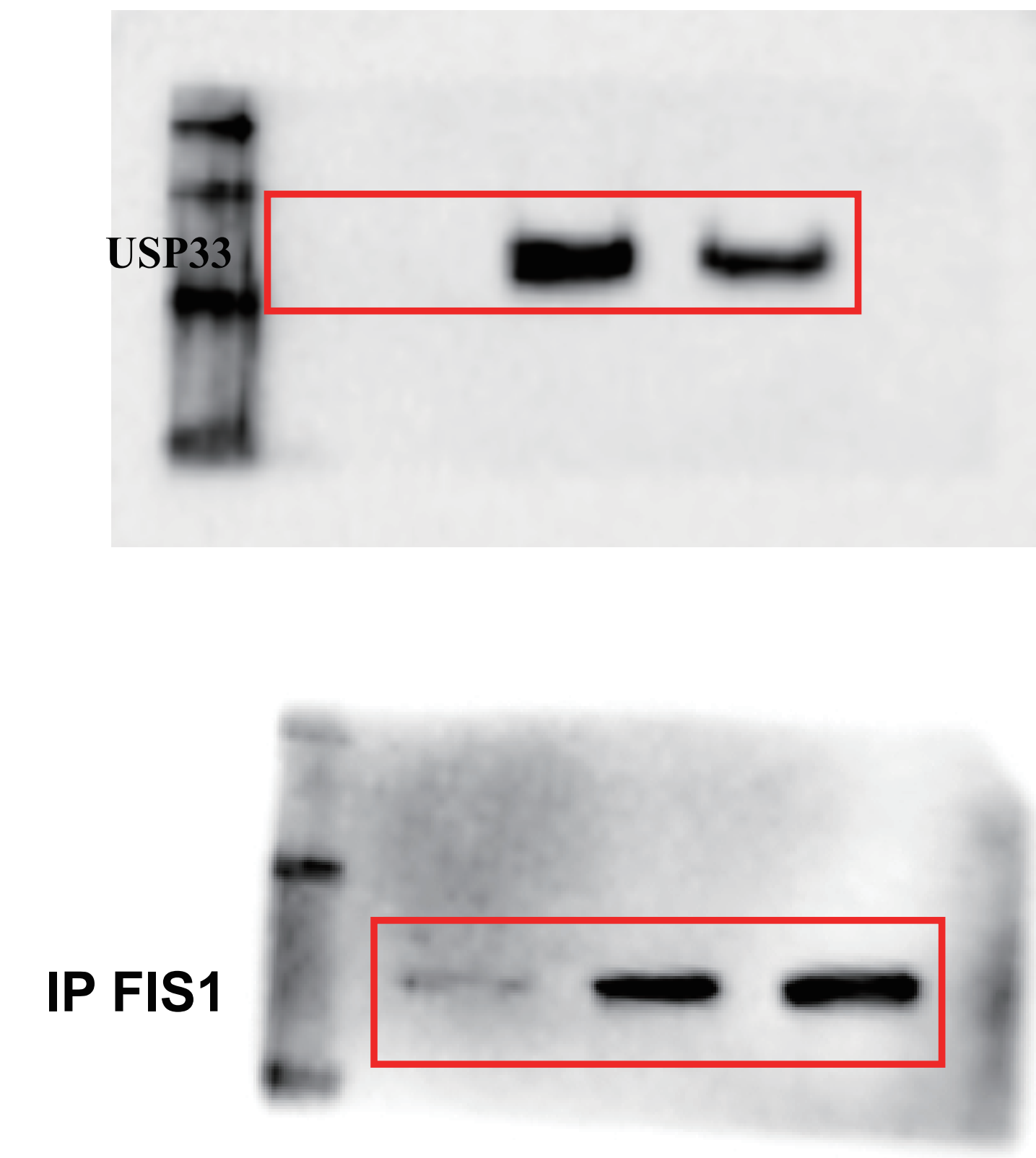

Fig5B

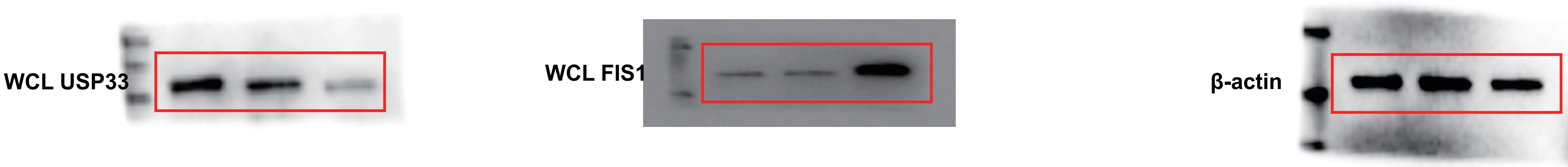

Fig5E

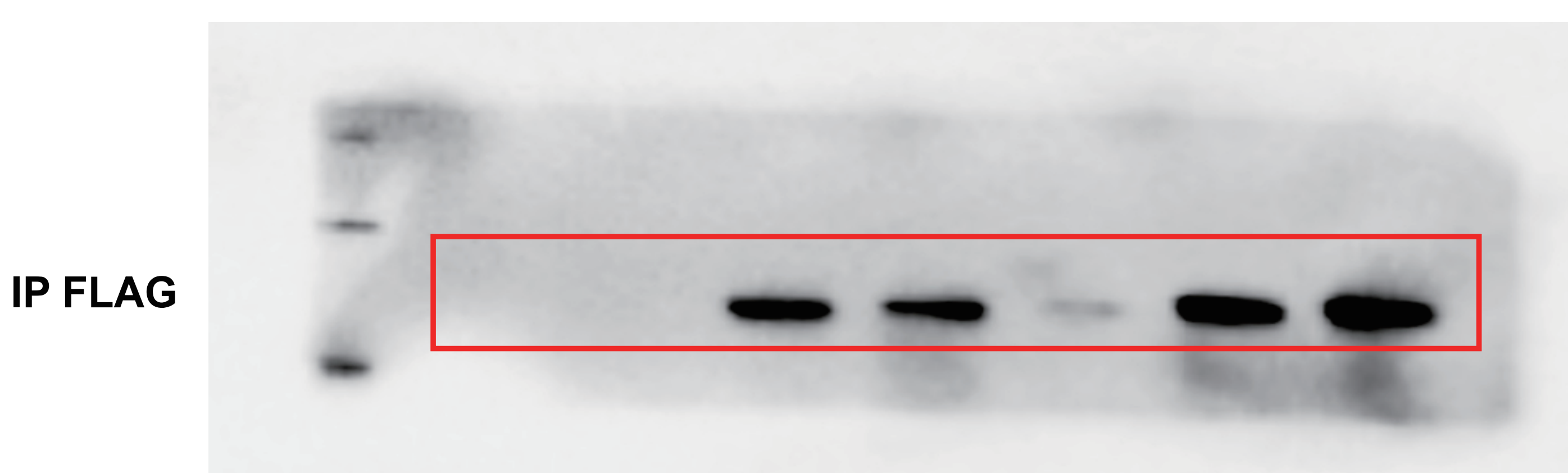

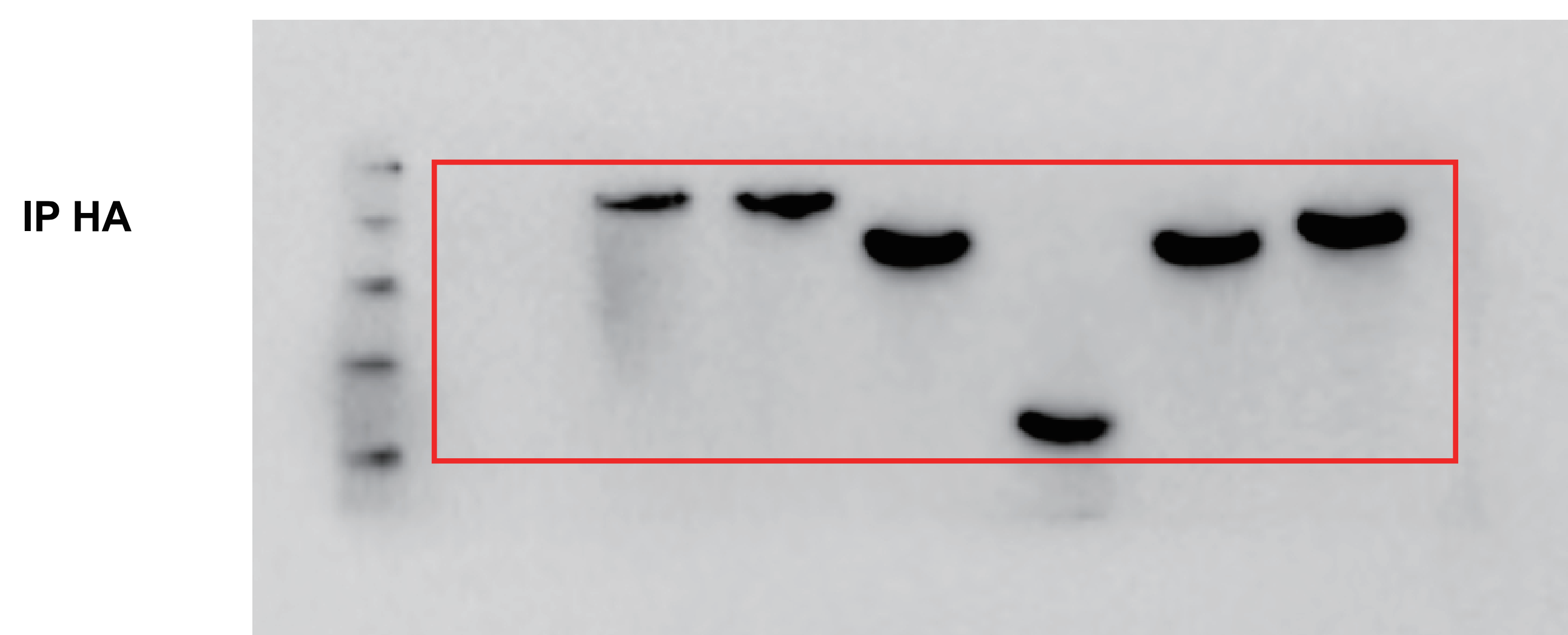

Fig5E

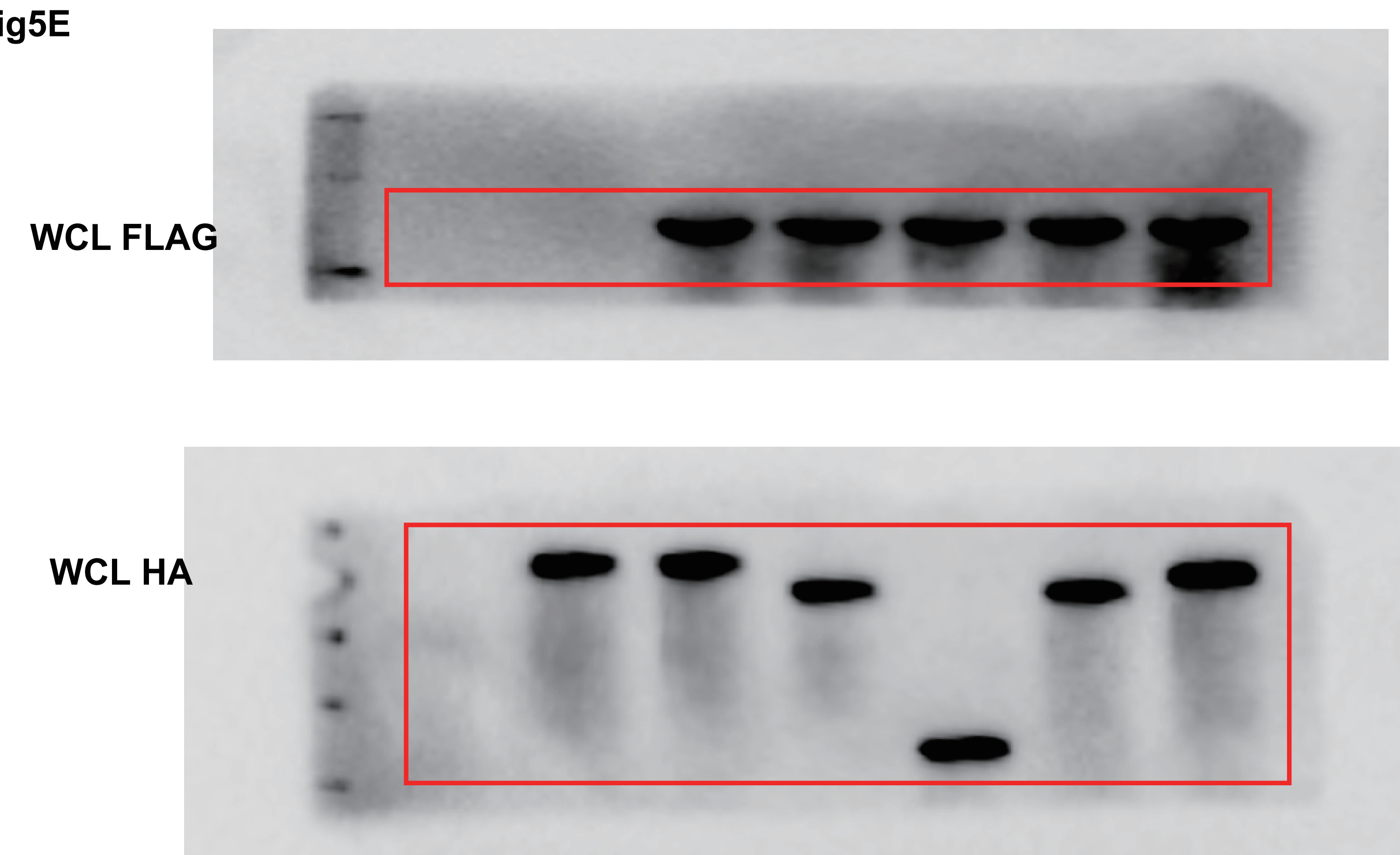

Fig5E

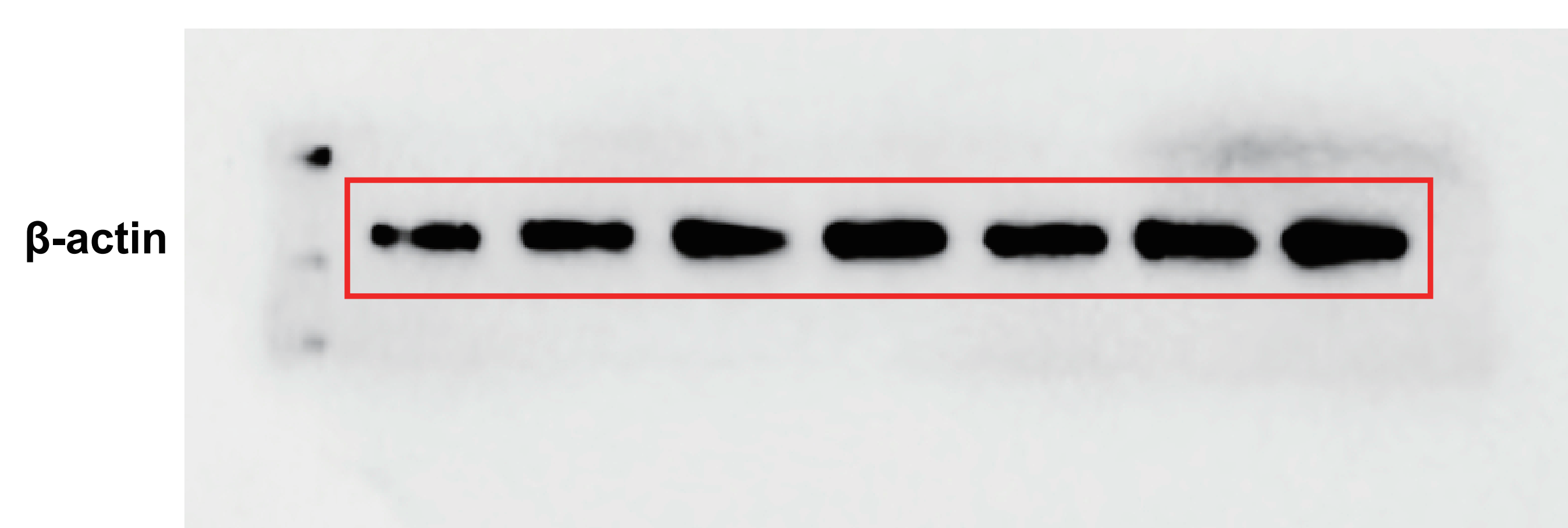

Fig5F

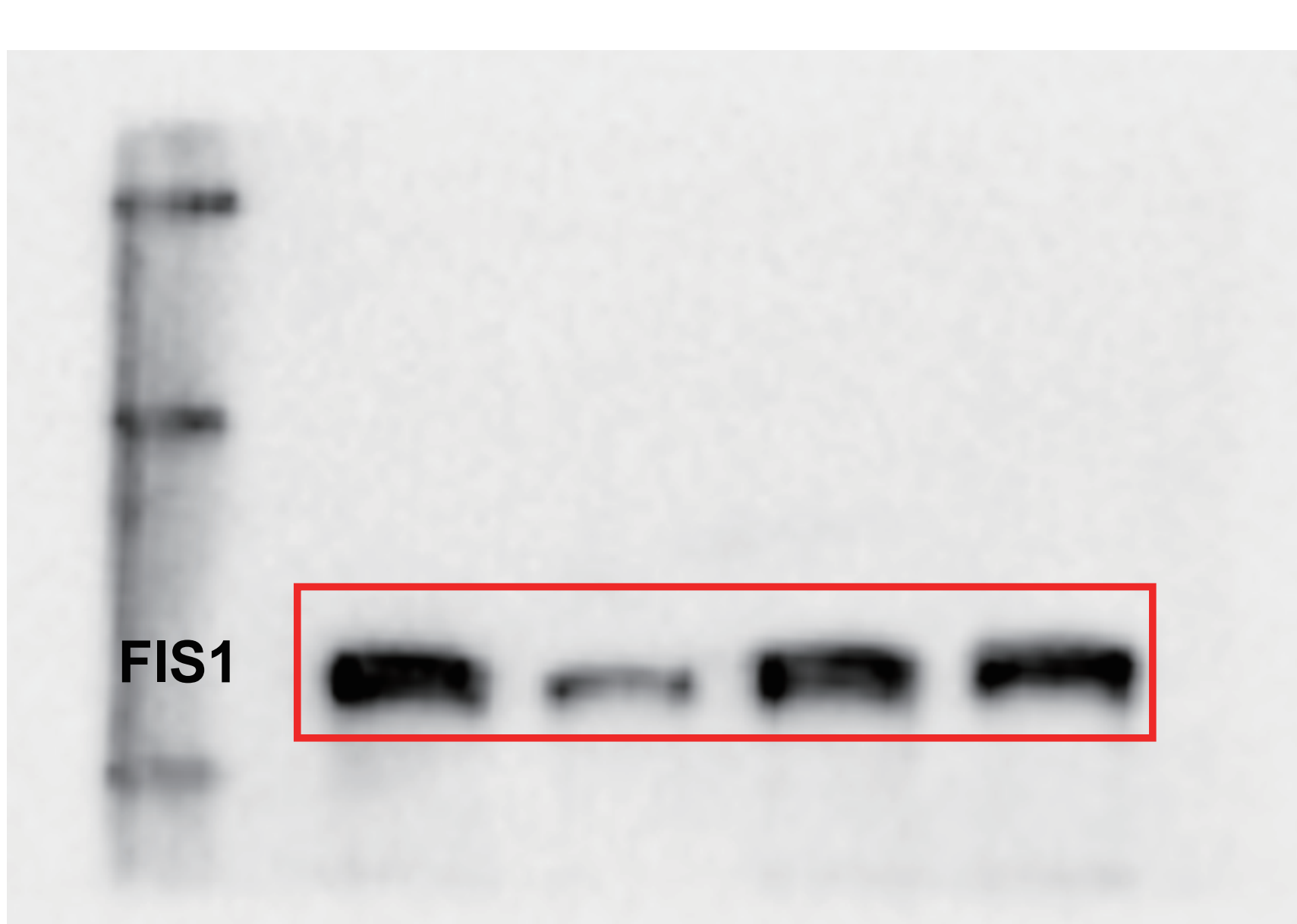

Fig5F

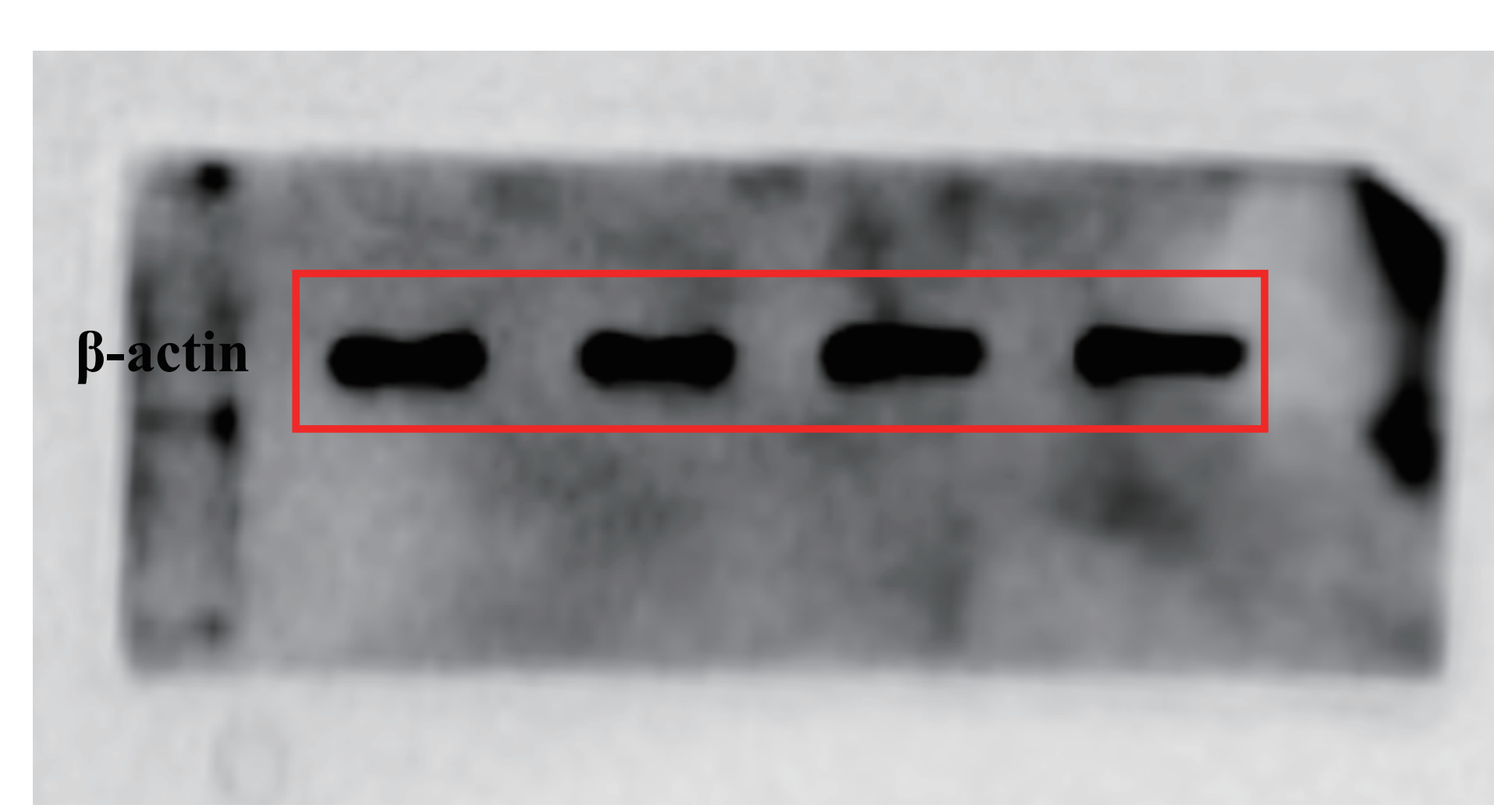

Fig5H

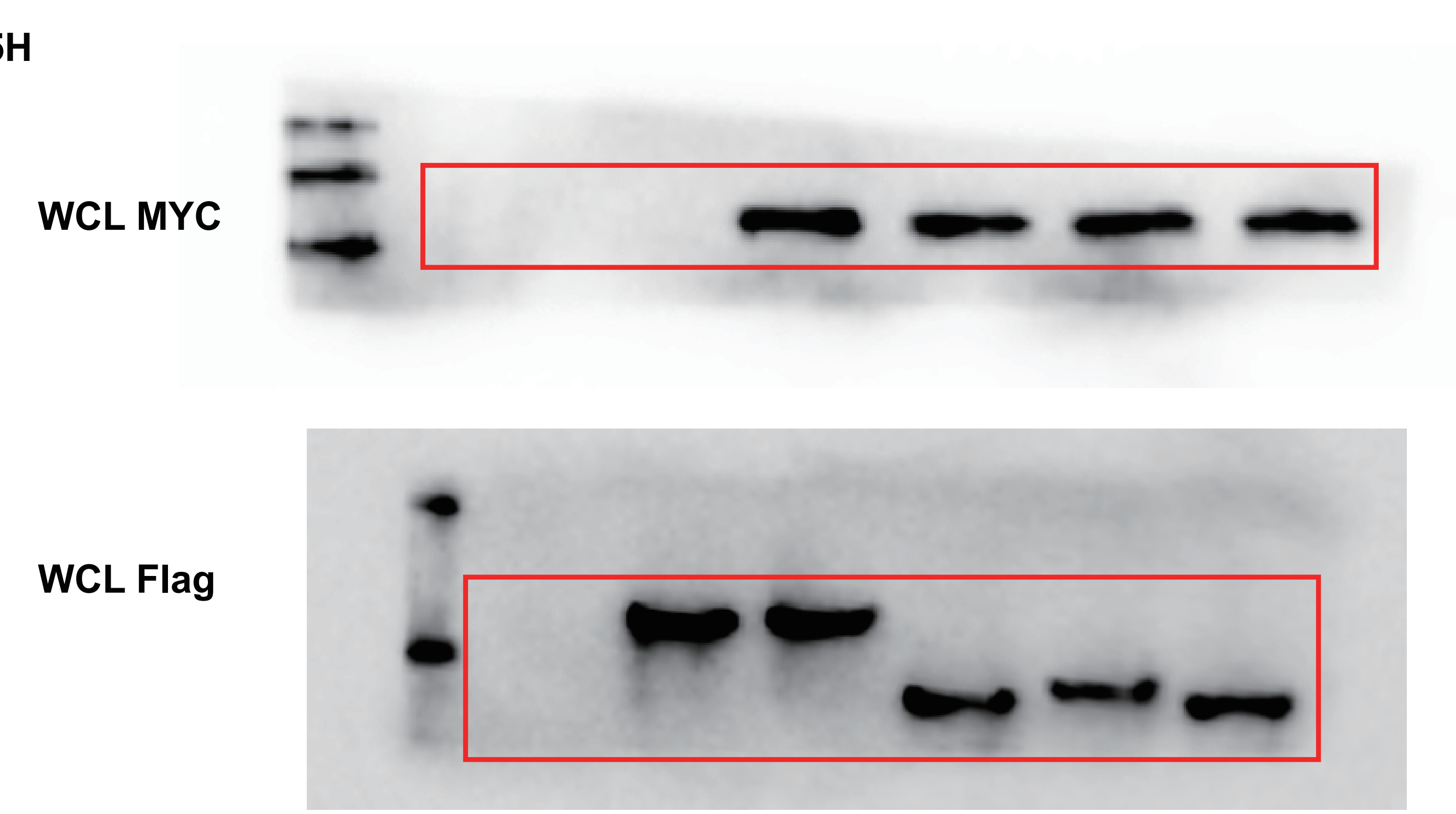

Fig5H

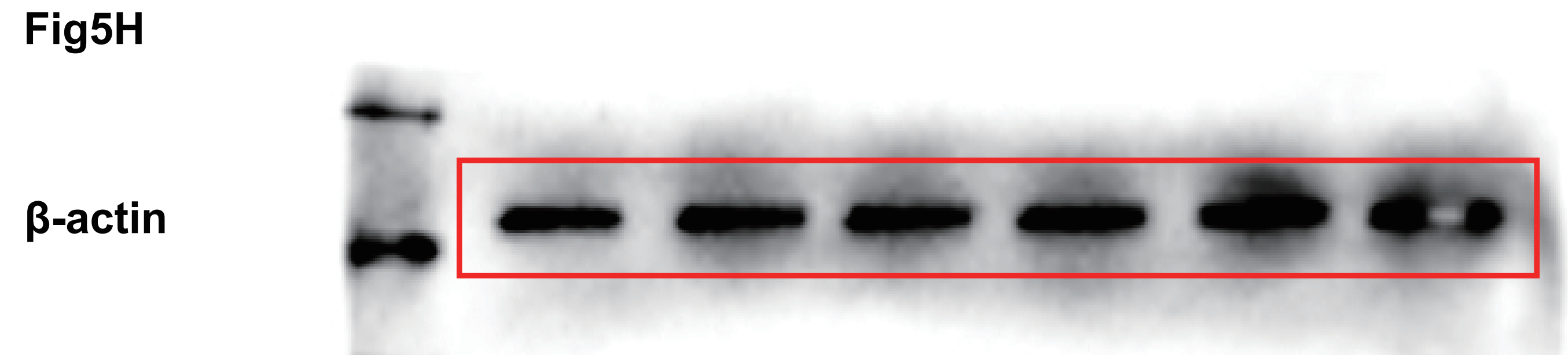

Fig5H

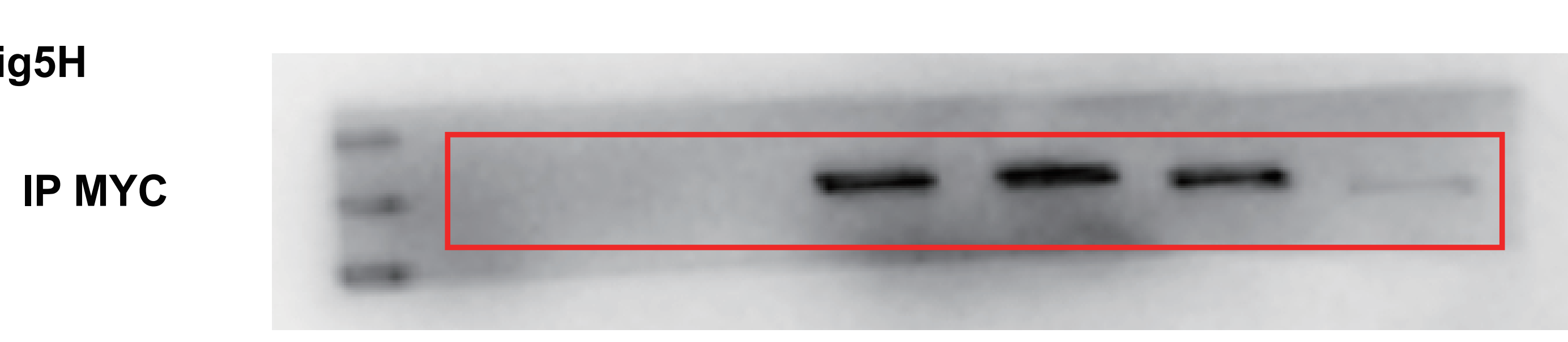

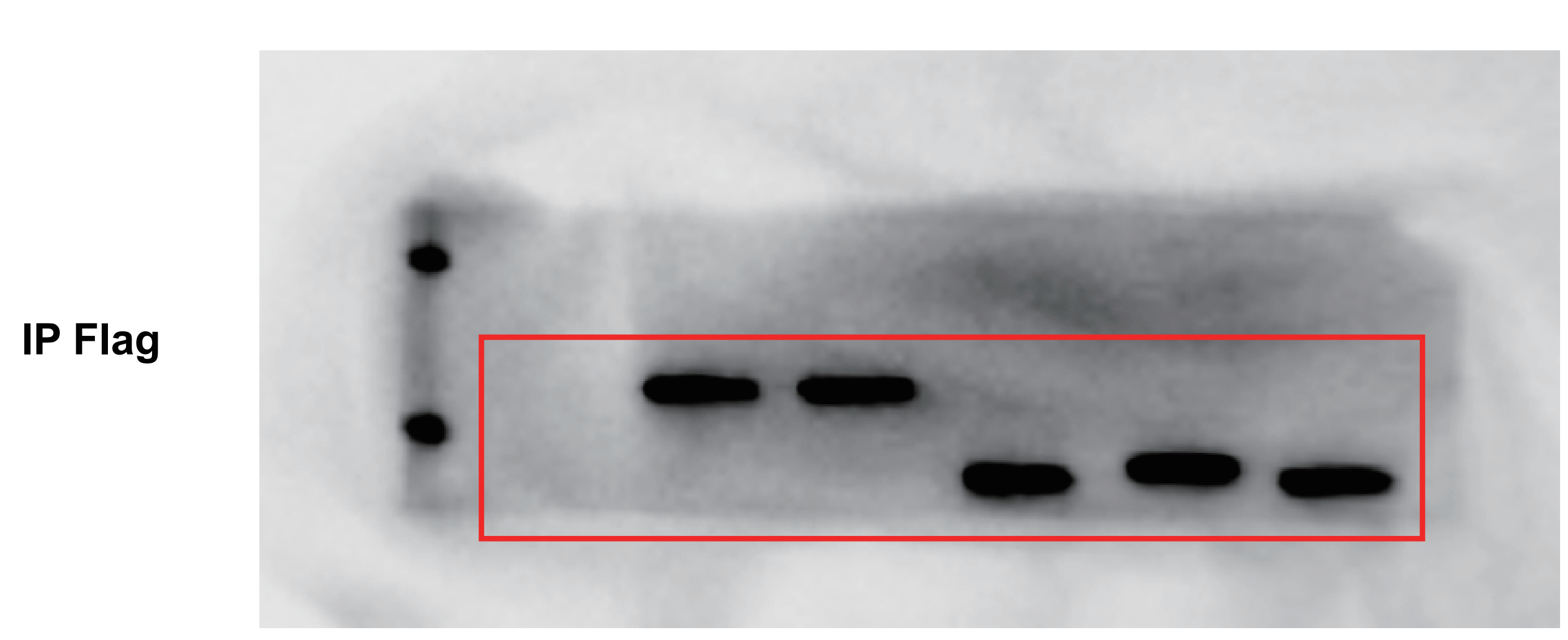

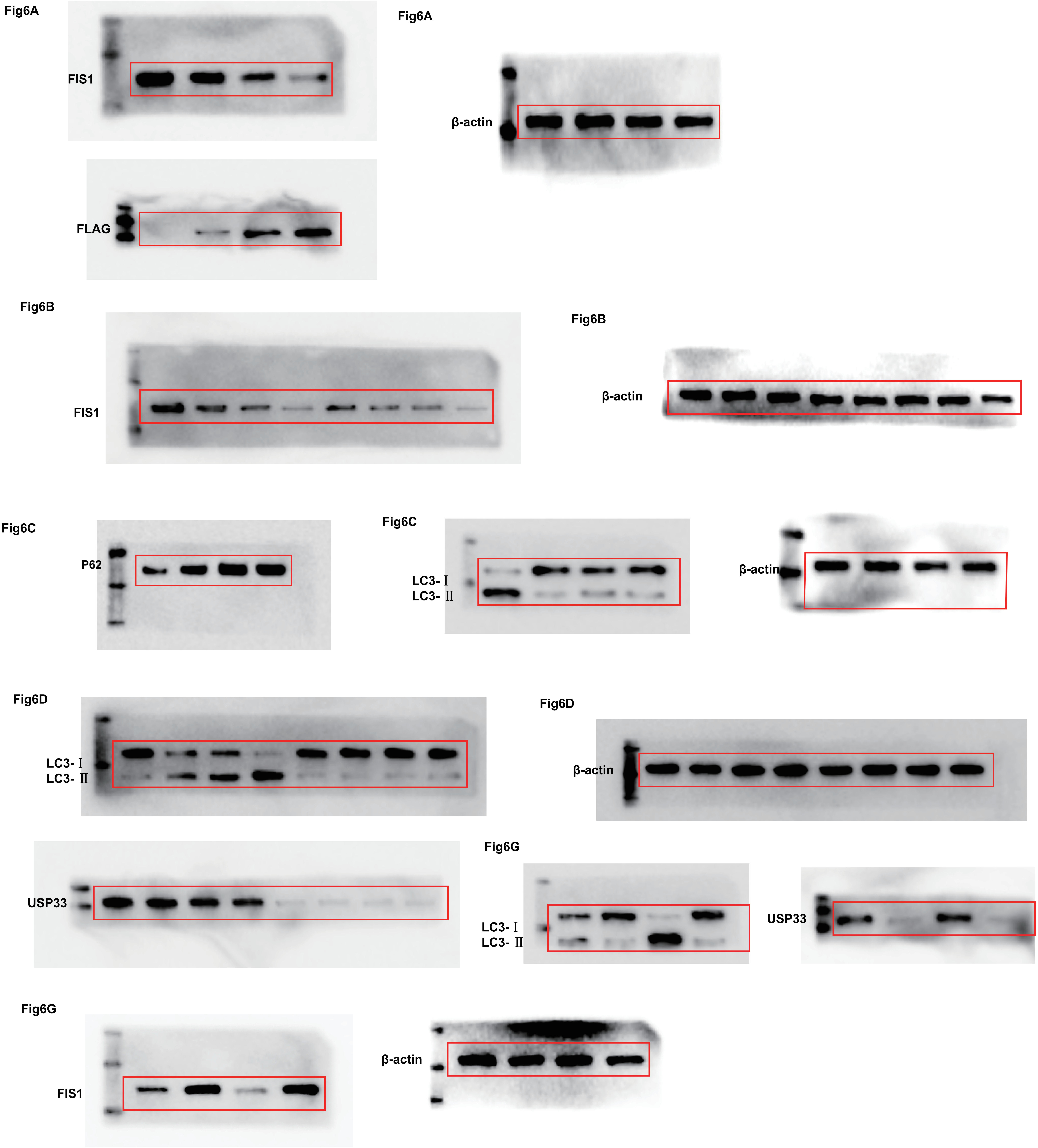
Fig6A
FIS1
FLAG
Fig6A
β-actin
Fig6B
FIS1
Fig6B
β-actin
Fig6C
P62
Fig6C
LC3- Ⅰ
LC3- Ⅱ
β-actin
Fig6D
LC3- Ⅰ
LC3- Ⅱ
Fig6D
β-actin
USP33
Fig6G
LC3- Ⅰ
LC3- Ⅱ
USP33
Fig6G
FIS1
β-actin

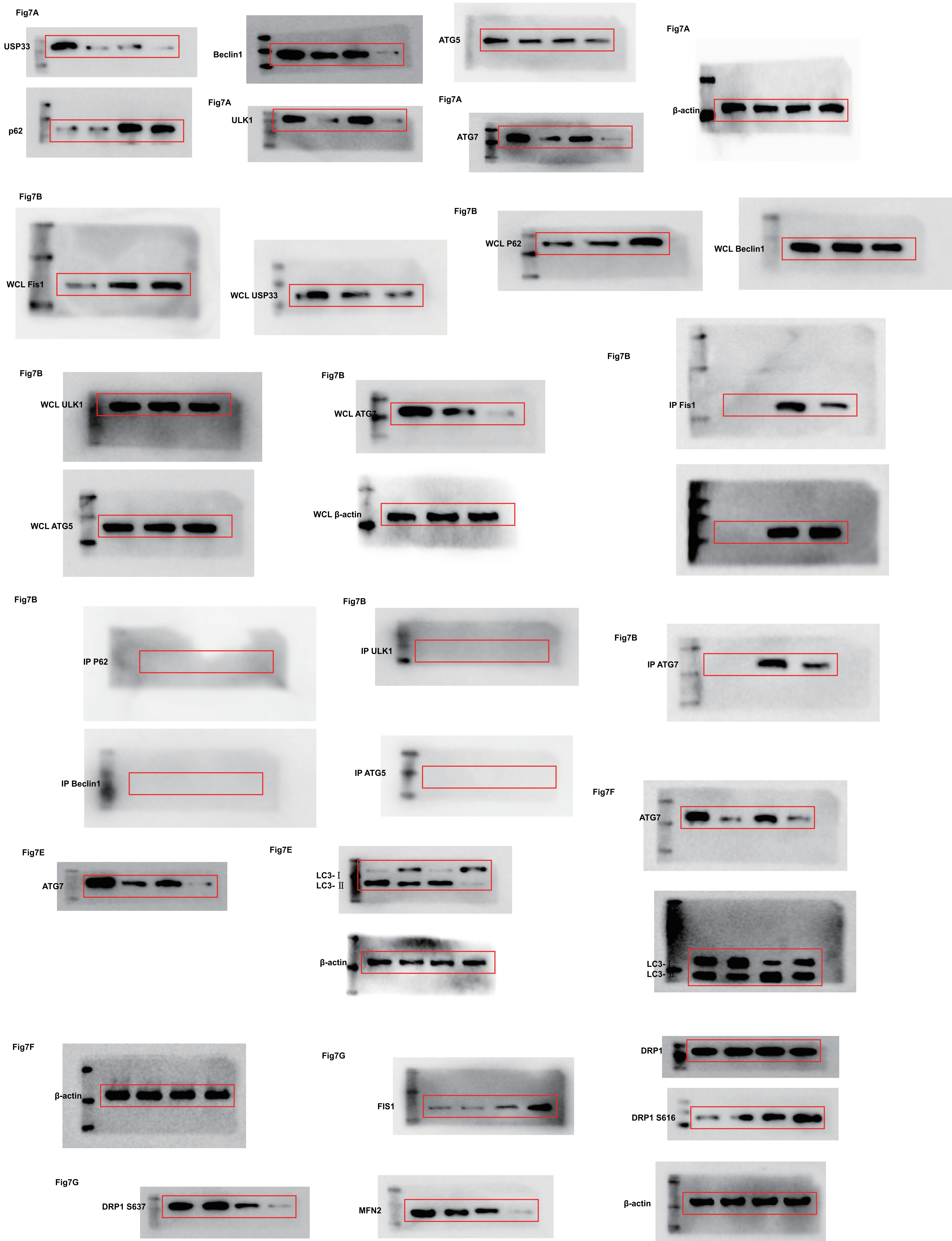

Fig7A
USP33
Beclin1
ATG5
Fig7A
p62
Fig7A
ULK1
Fig7A
ATG7
β-actin
Fig7B
WCL Fis1
WCL USP33
Fig7B
WCL P62
WCL Beclin1
Fig7B
WCL ULK1
Fig7B
WCL ATG7
Fig7B
IP Fis1
WCL ATG5
WCL β-actin
Fig7B
IP P62
Fig7B
IP ULK1
Fig7B
IP ATG7
IP Beclin1
IP ATG5
Fig7F
ATG7
Fig7E
ATG7
Fig7E
LC3- Ⅰ
LC3- Ⅱ
β-actin
LC3- Ⅰ
LC3- Ⅱ
Fig7F
β-actin
Fig7G
FIS1
DRP1
DRP1 S616
Fig7G
DRP1 S637
MFN2
β-actin

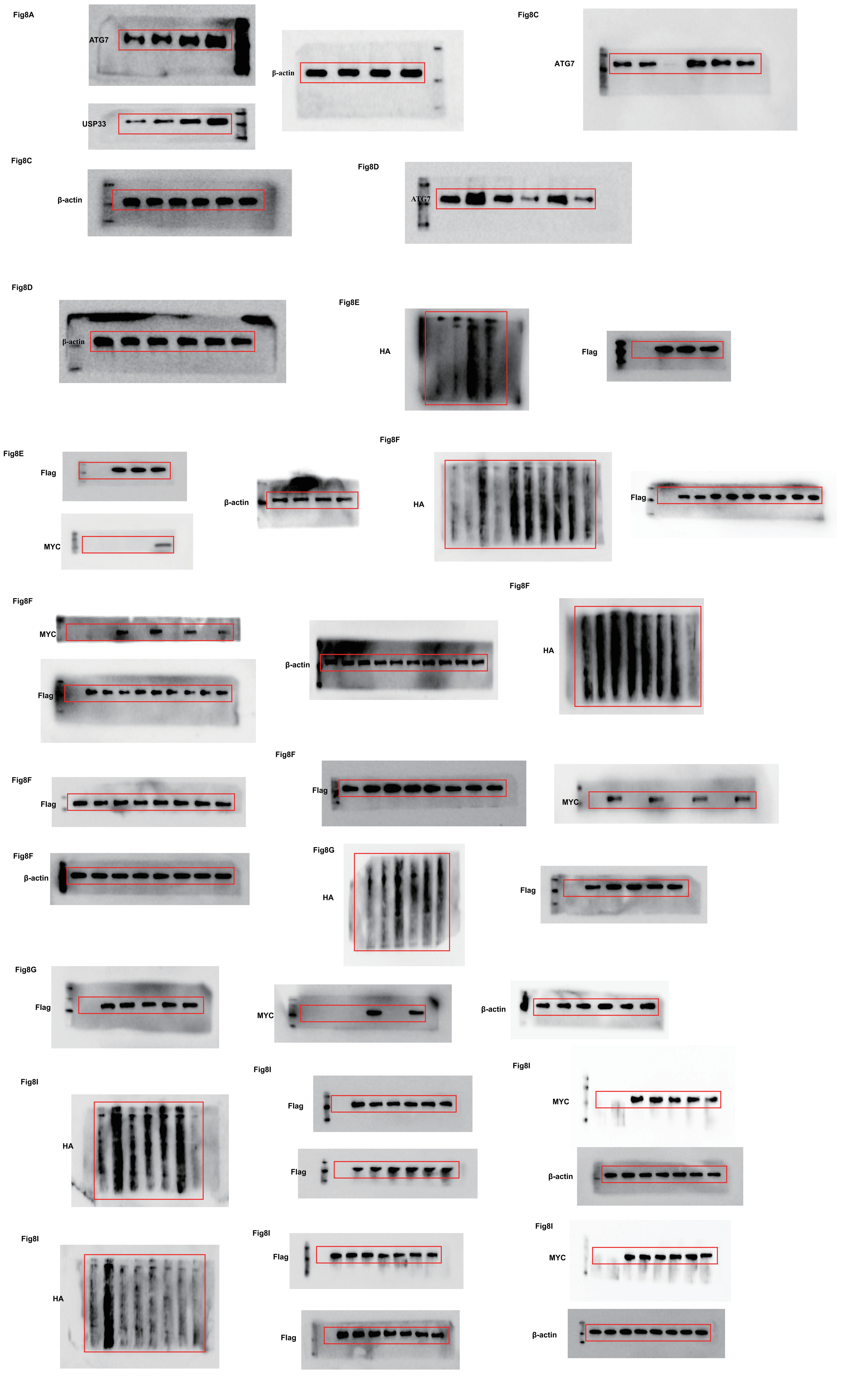
Fig8A
ATG7
β-actin
USP33
Fig8C
ATG7
Fig8C
β-actin
Fig8D
ATG7
Fig8D
β-actin
Fig8E
HA
Flag
Fig8E
Flag
β-actin
MYC
Fig8F
HA
Flag
Fig8F
MYC
β-actin
Flag
Fig8F
HA
Fig8F
Flag
Fig8F
Flag
MYC
Fig8F
β-actin
Fig8G
HA
Flag
Fig8G
Flag
MYC
β-actin
Fig8I
HA
Fig8I
Flag
Flag
Fig8I
MYC
β-actin
Fig8I
HA
Fig8I
Flag
Flag
Fig8I
MYC
β-actin

Fig9A

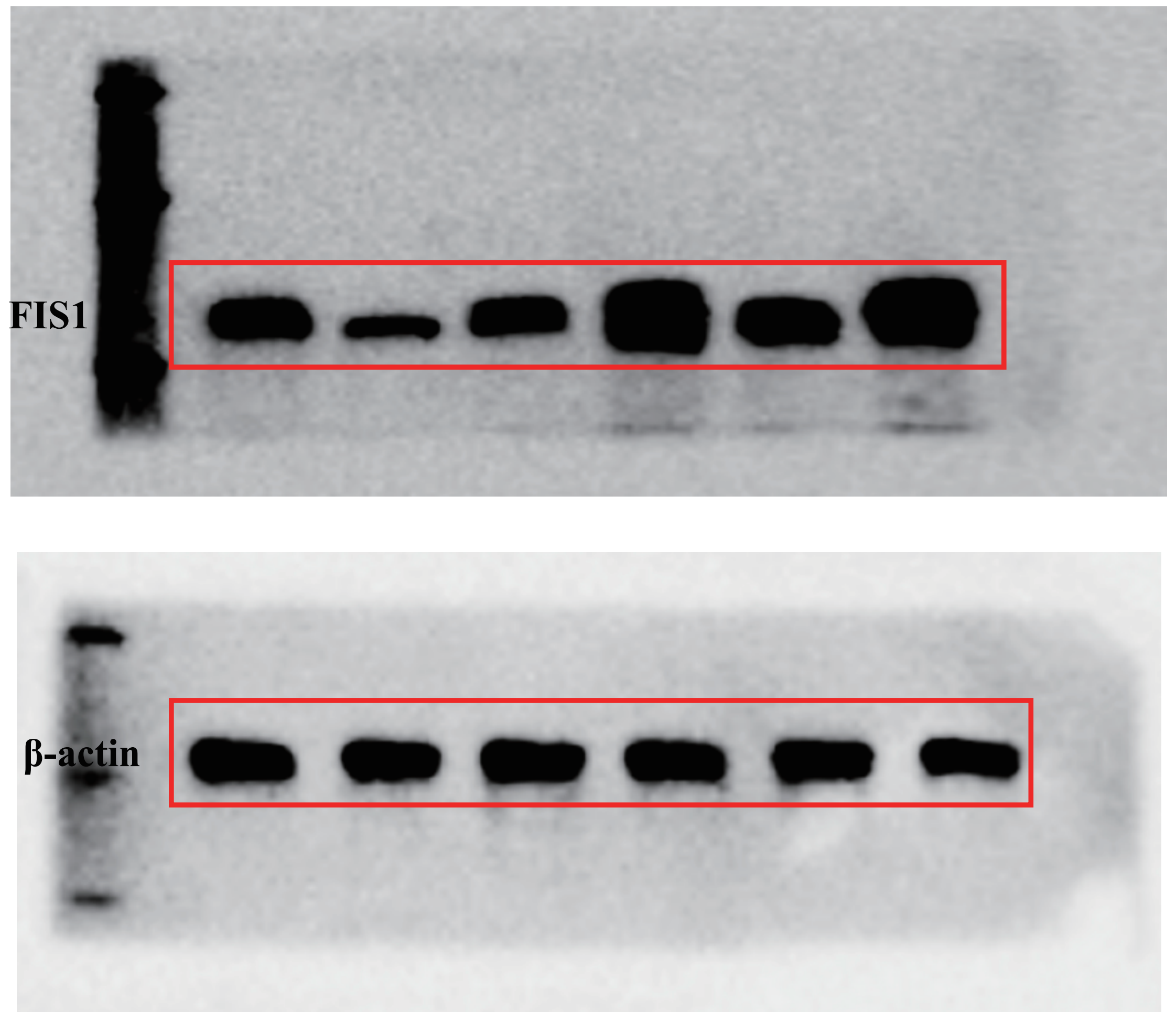

Fig9K

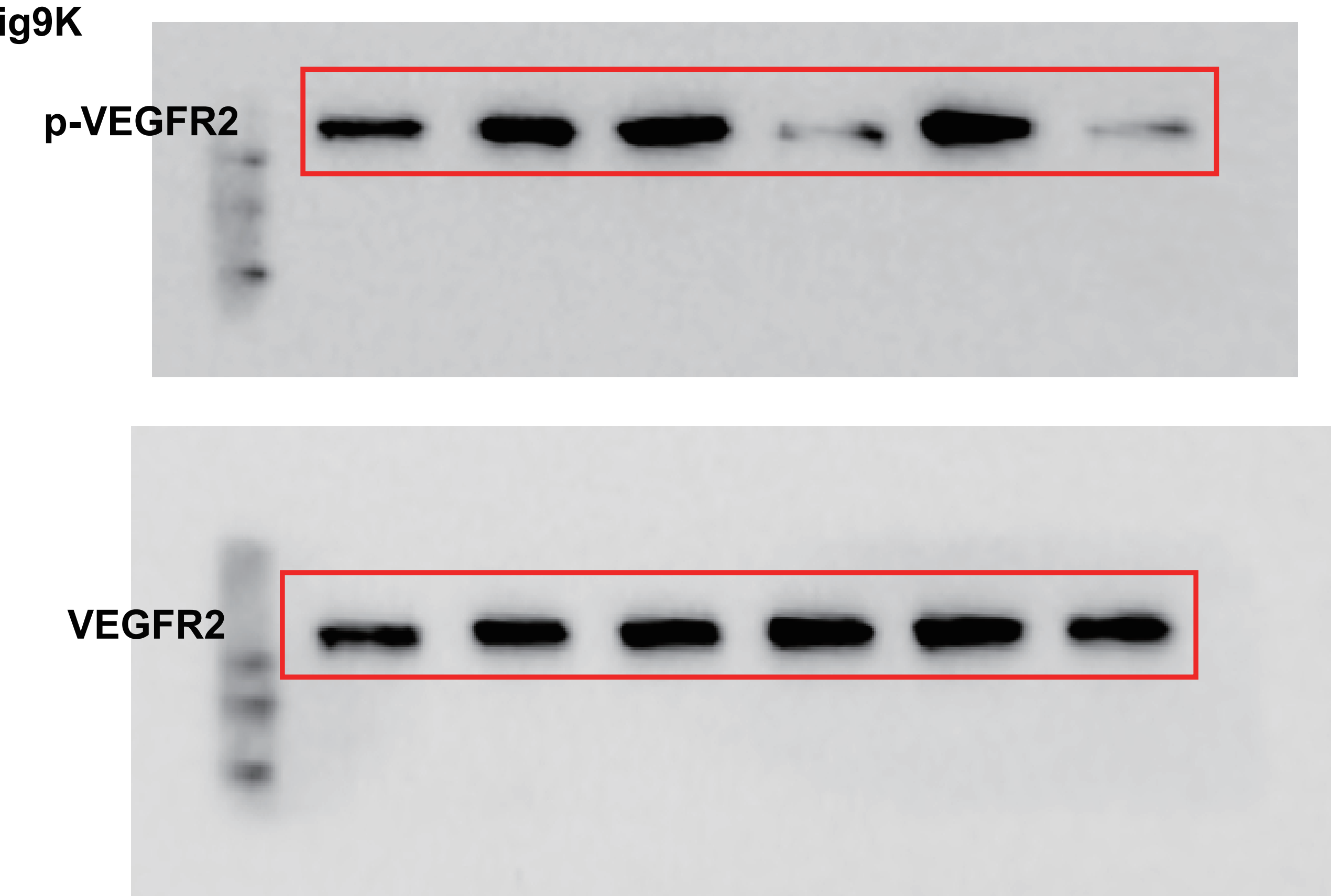

Fig9K

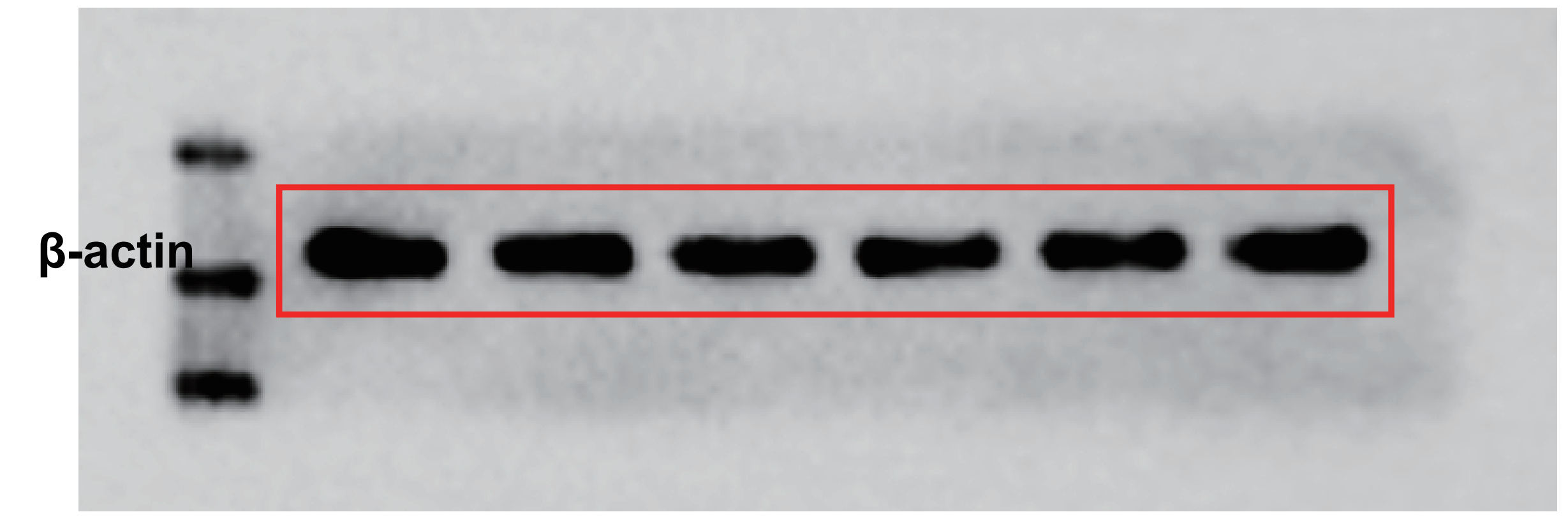

Fig9L

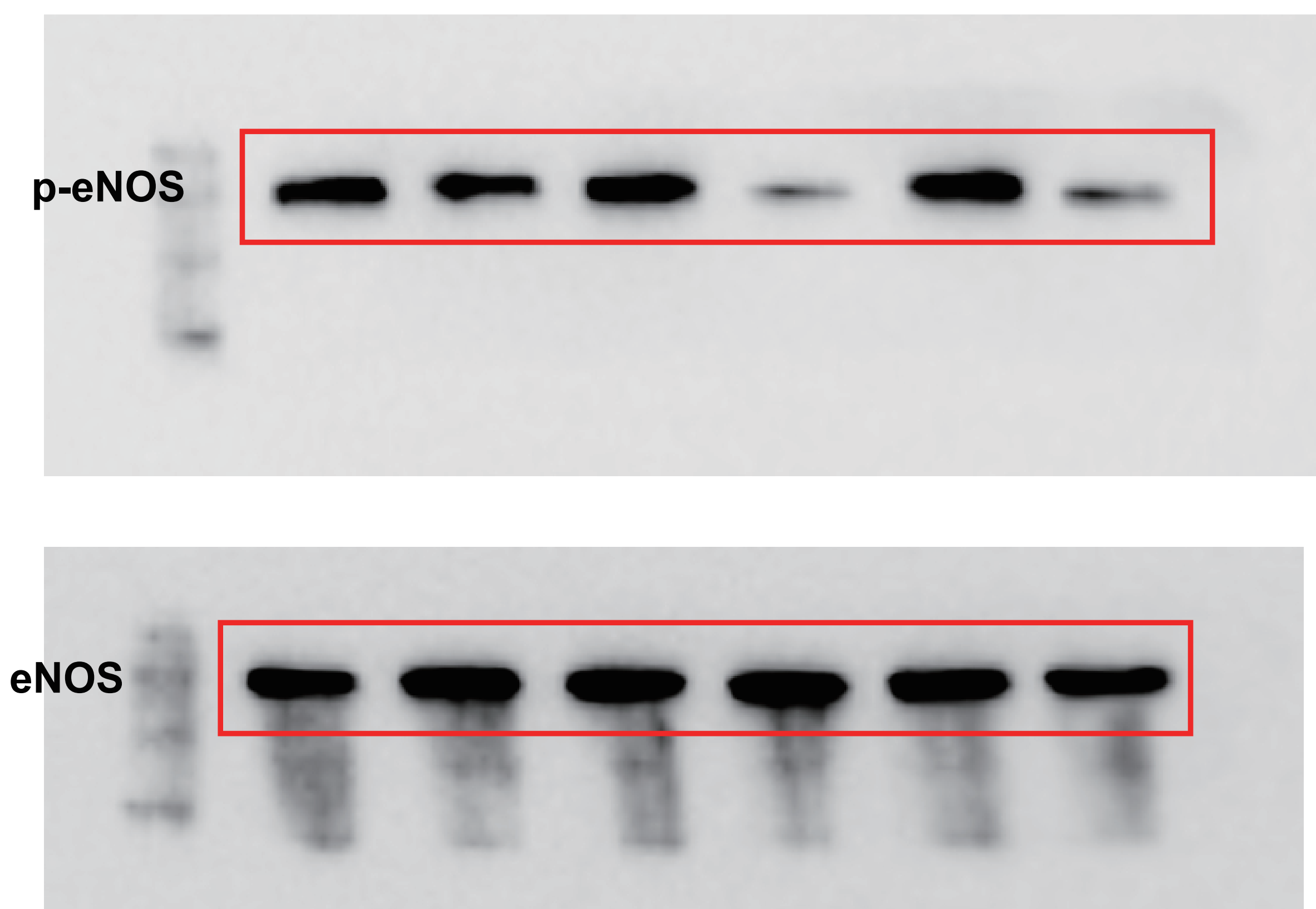

Fig9L

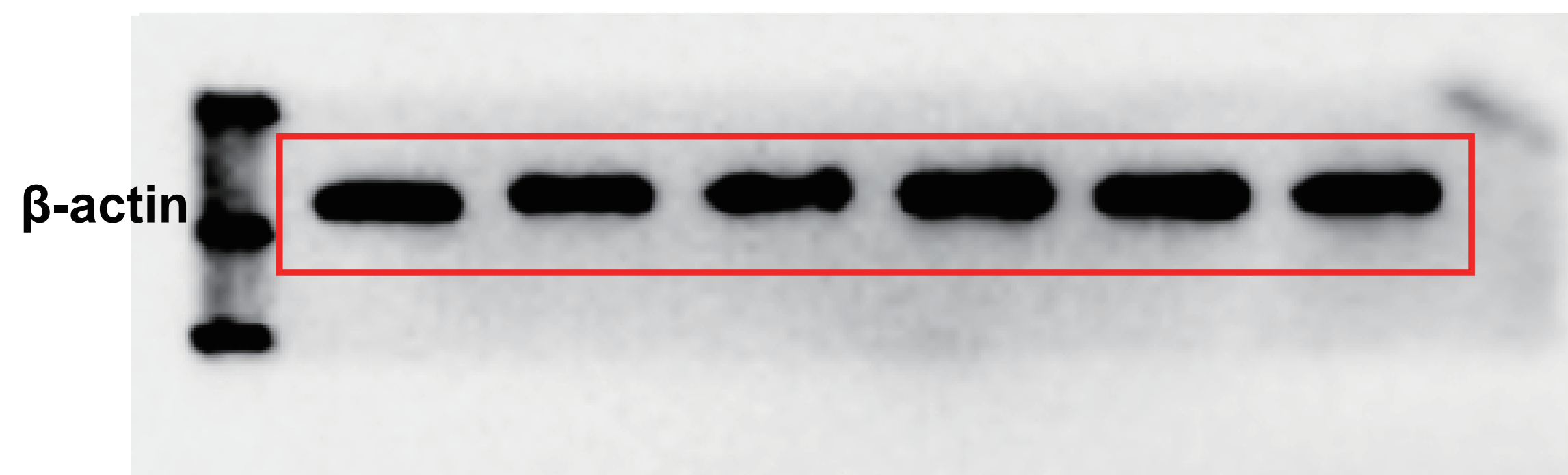

Fig9N

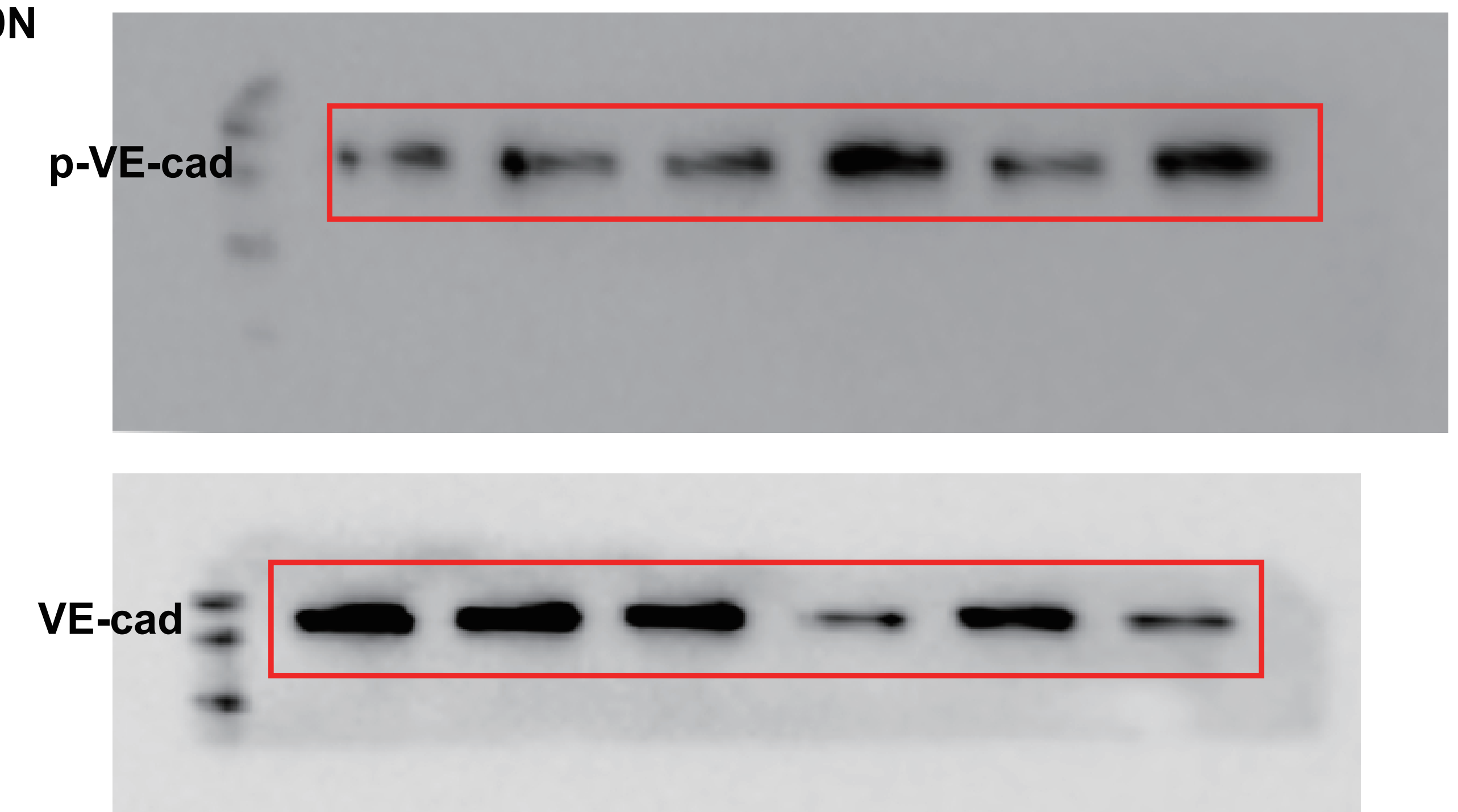

Fig9N

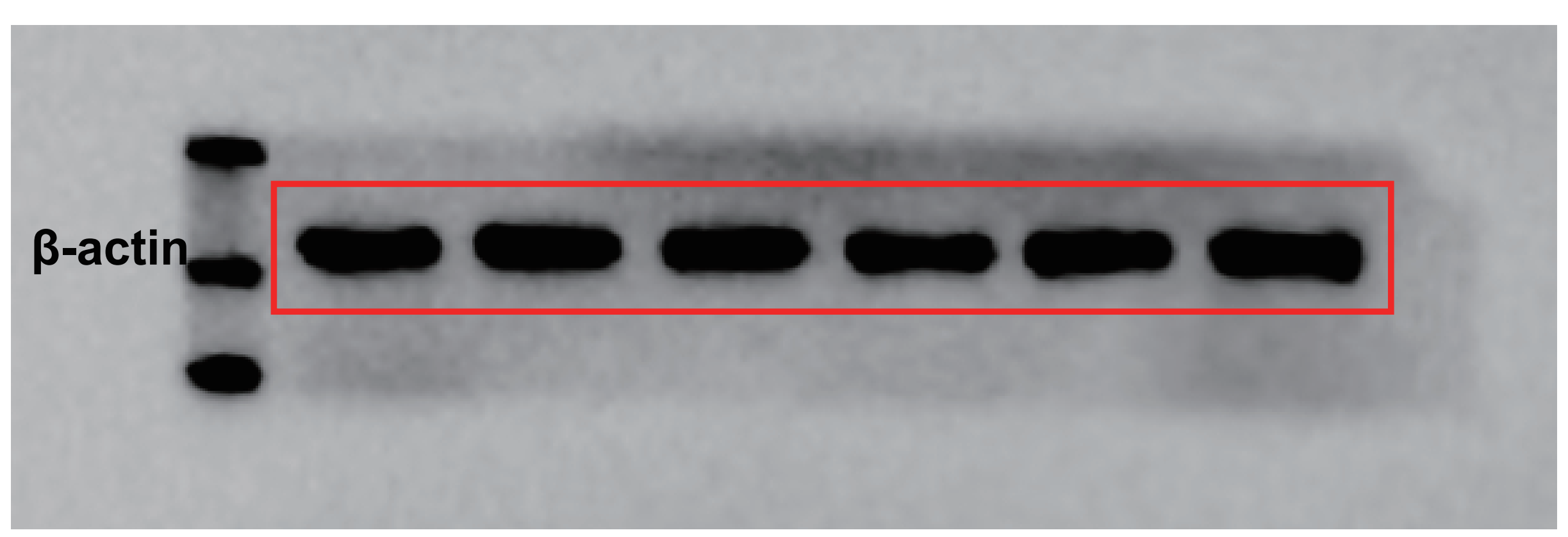

Fig9O

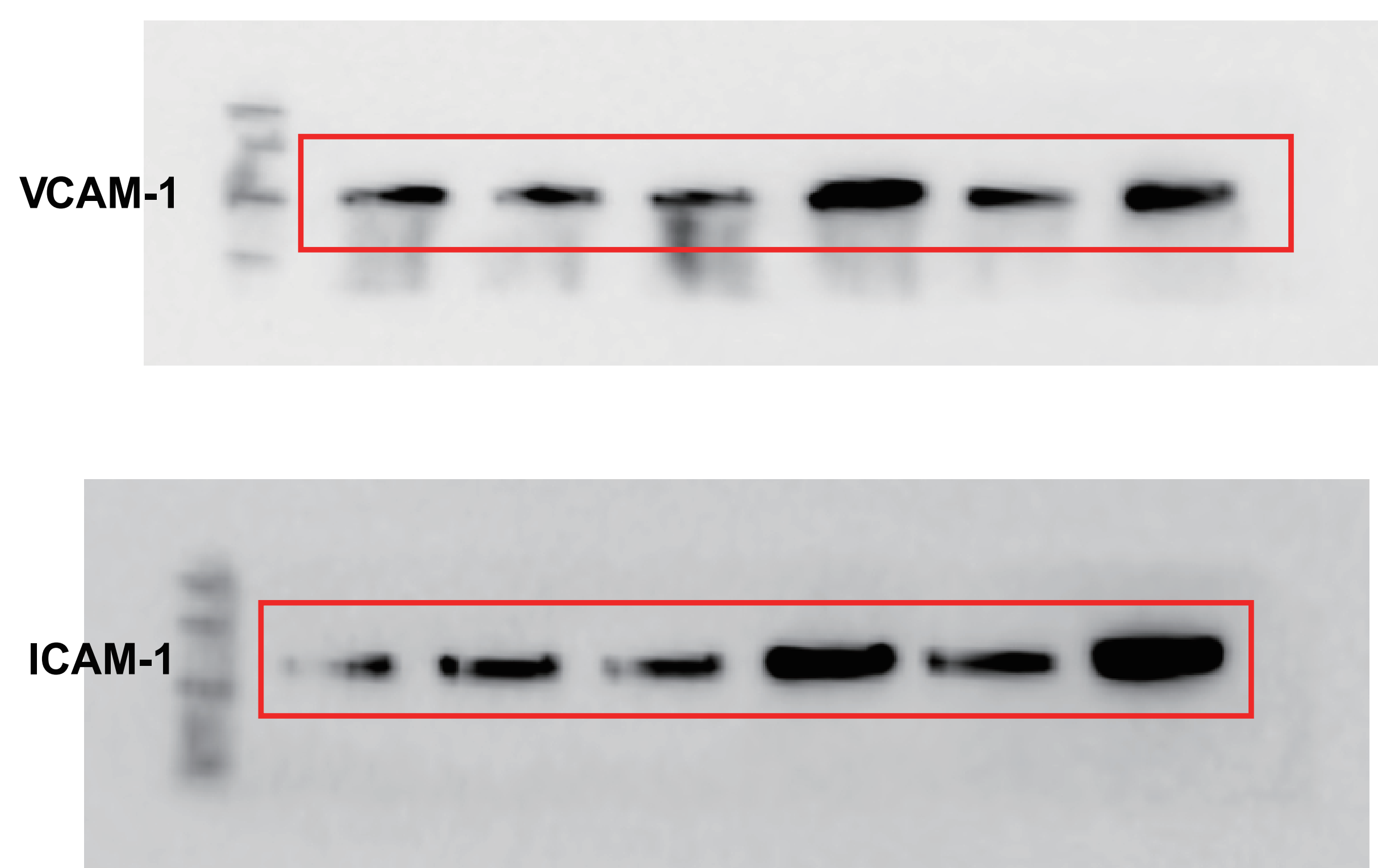

Fig9O

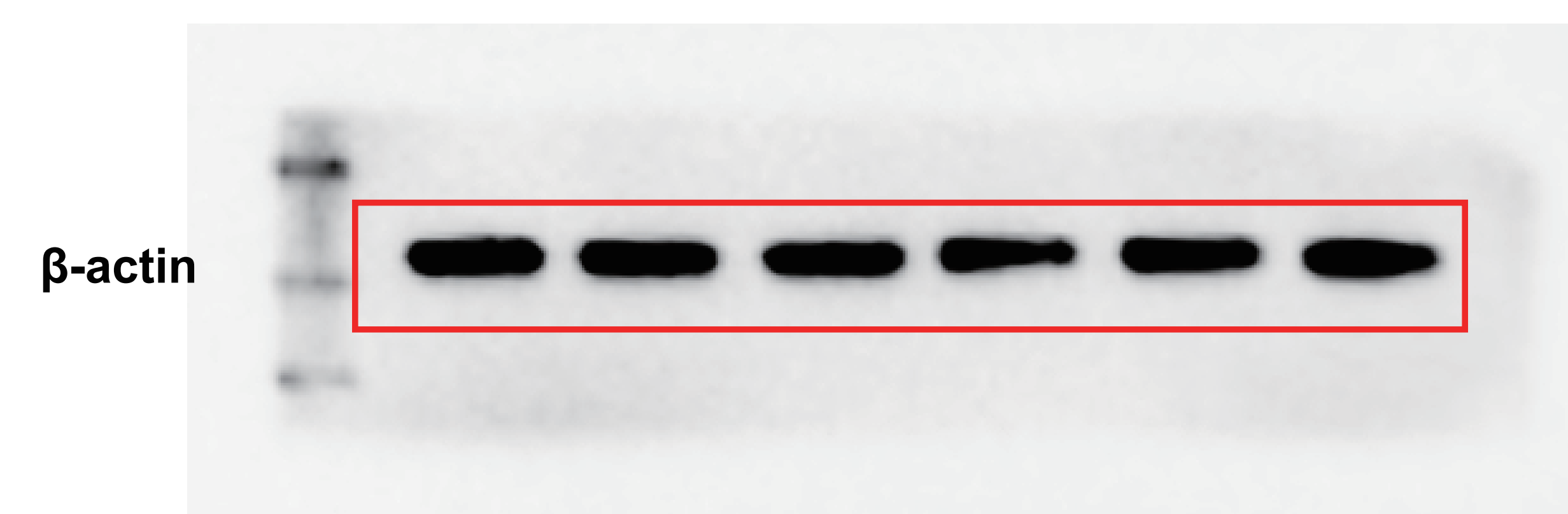

Fig10C

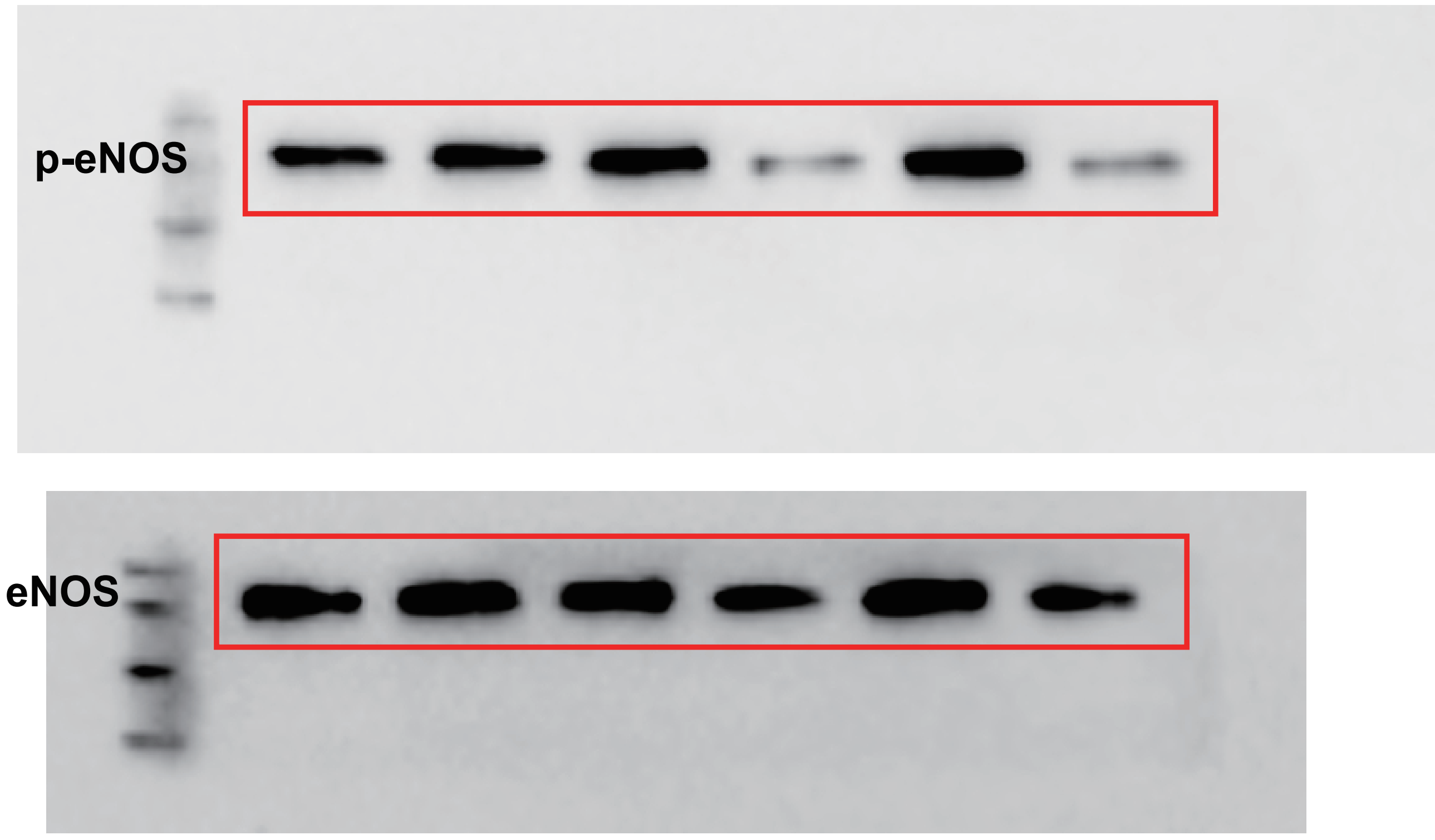

Fig10C

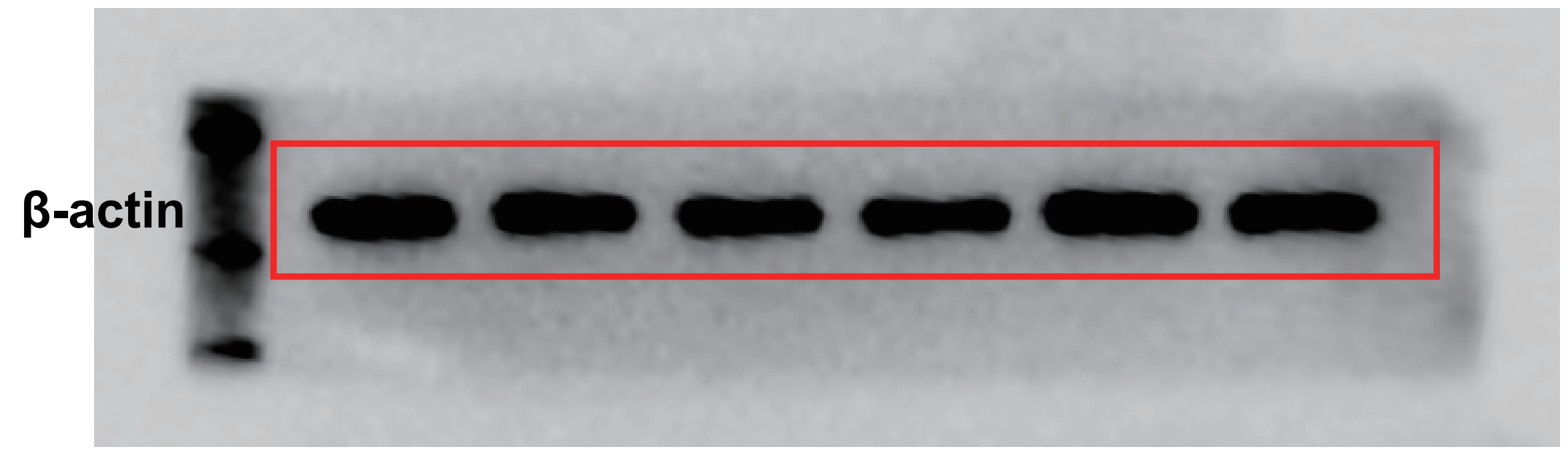

Fig10D

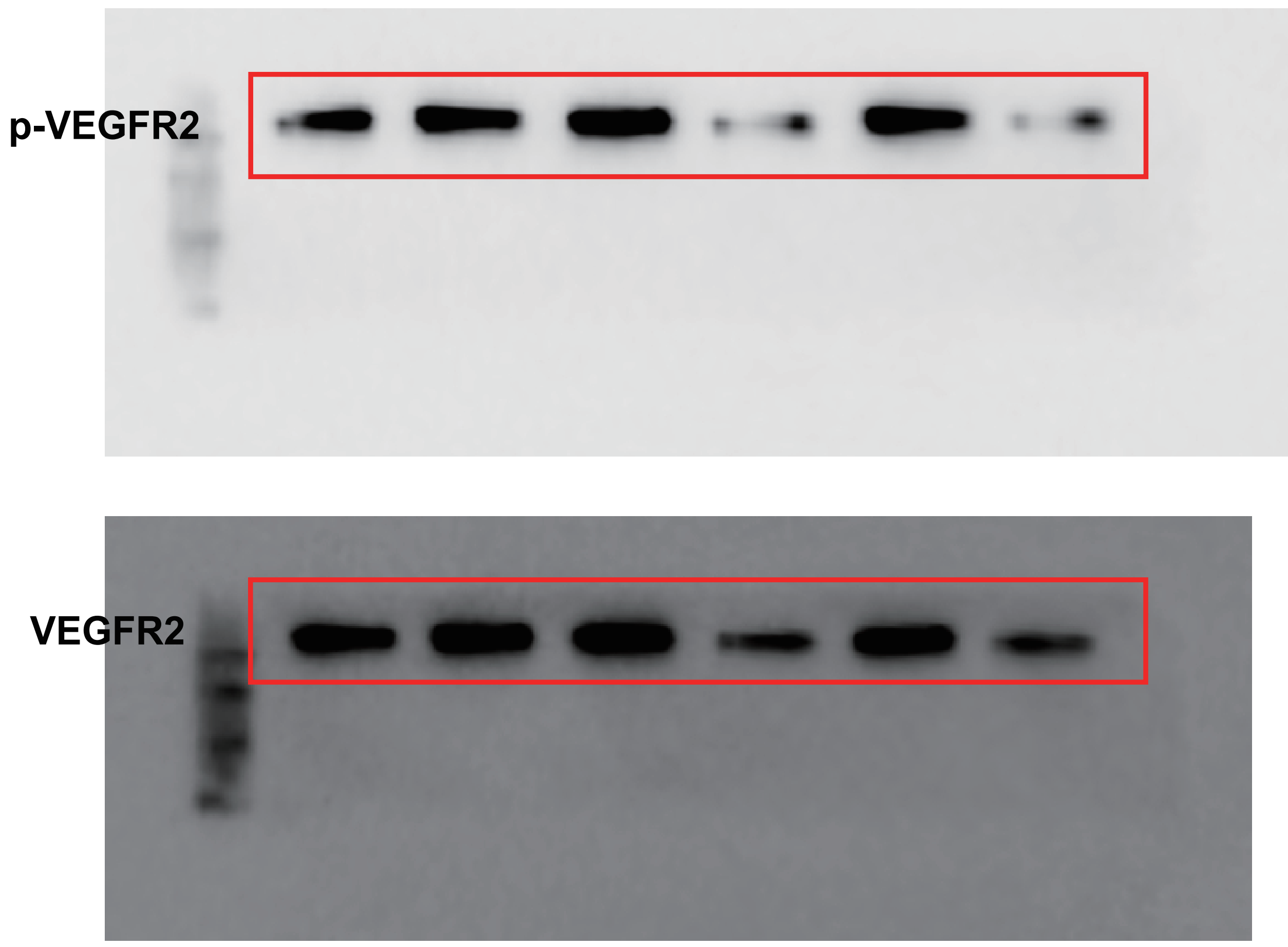

Fig10D

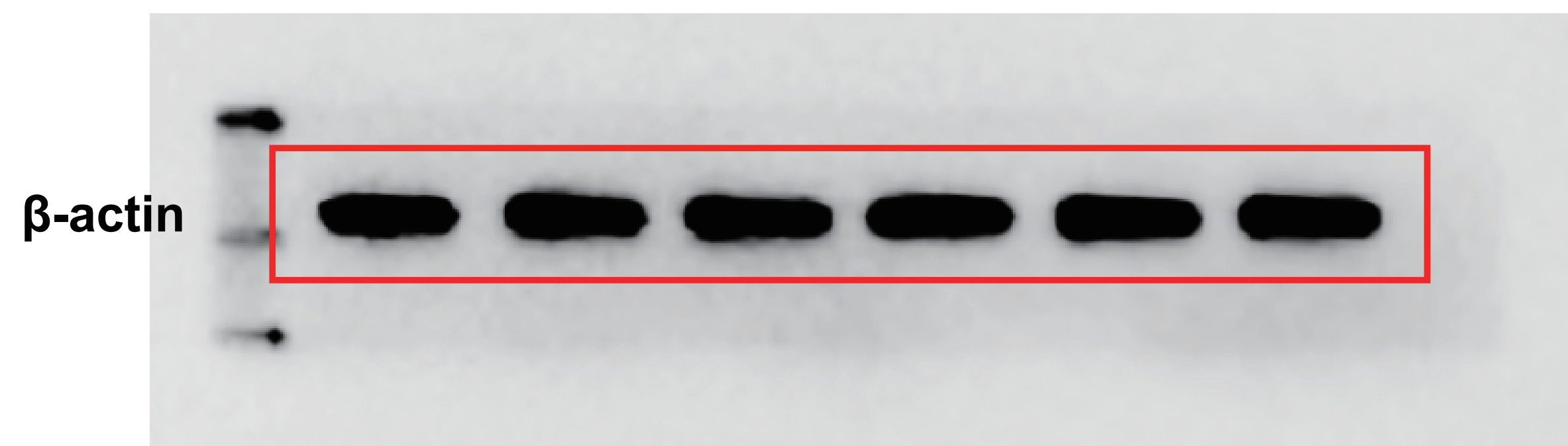

Fig10E

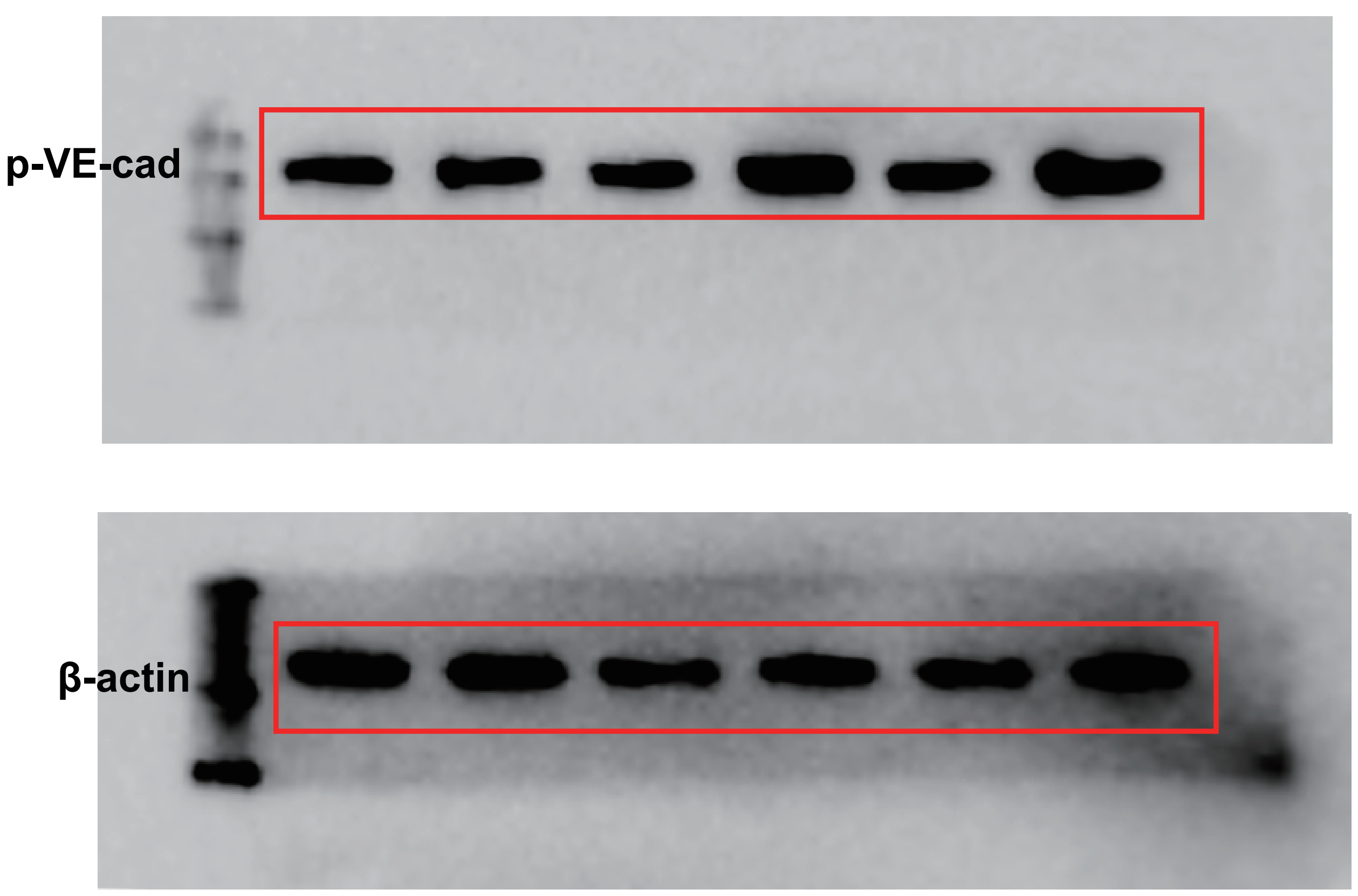

Fig10E

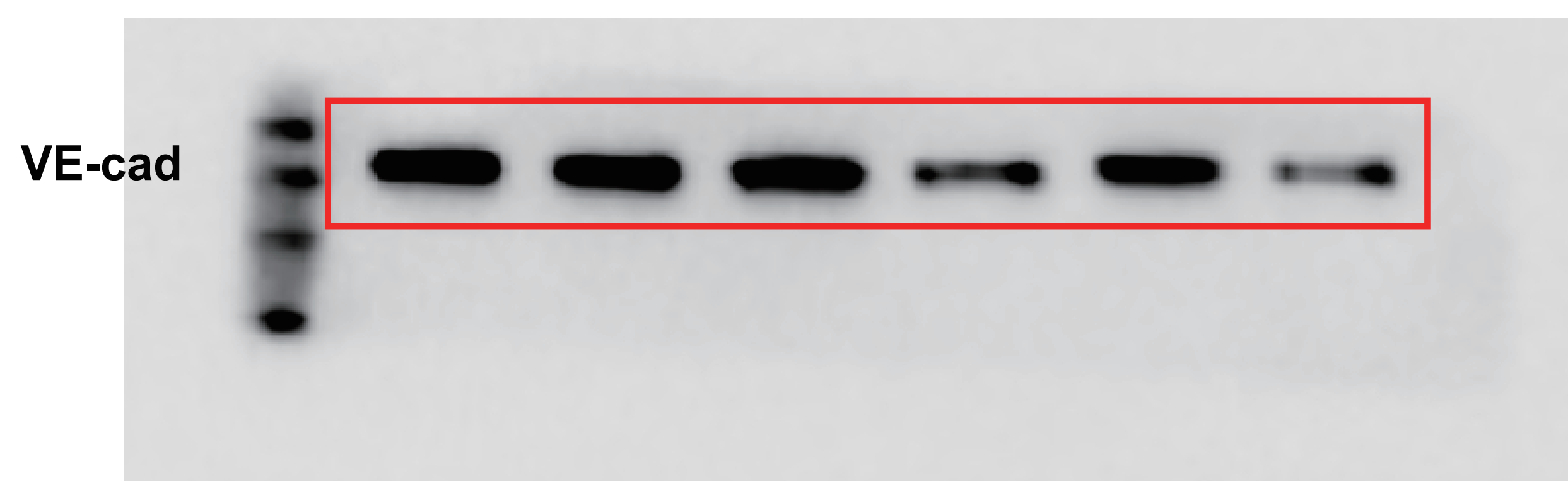

FigS1E

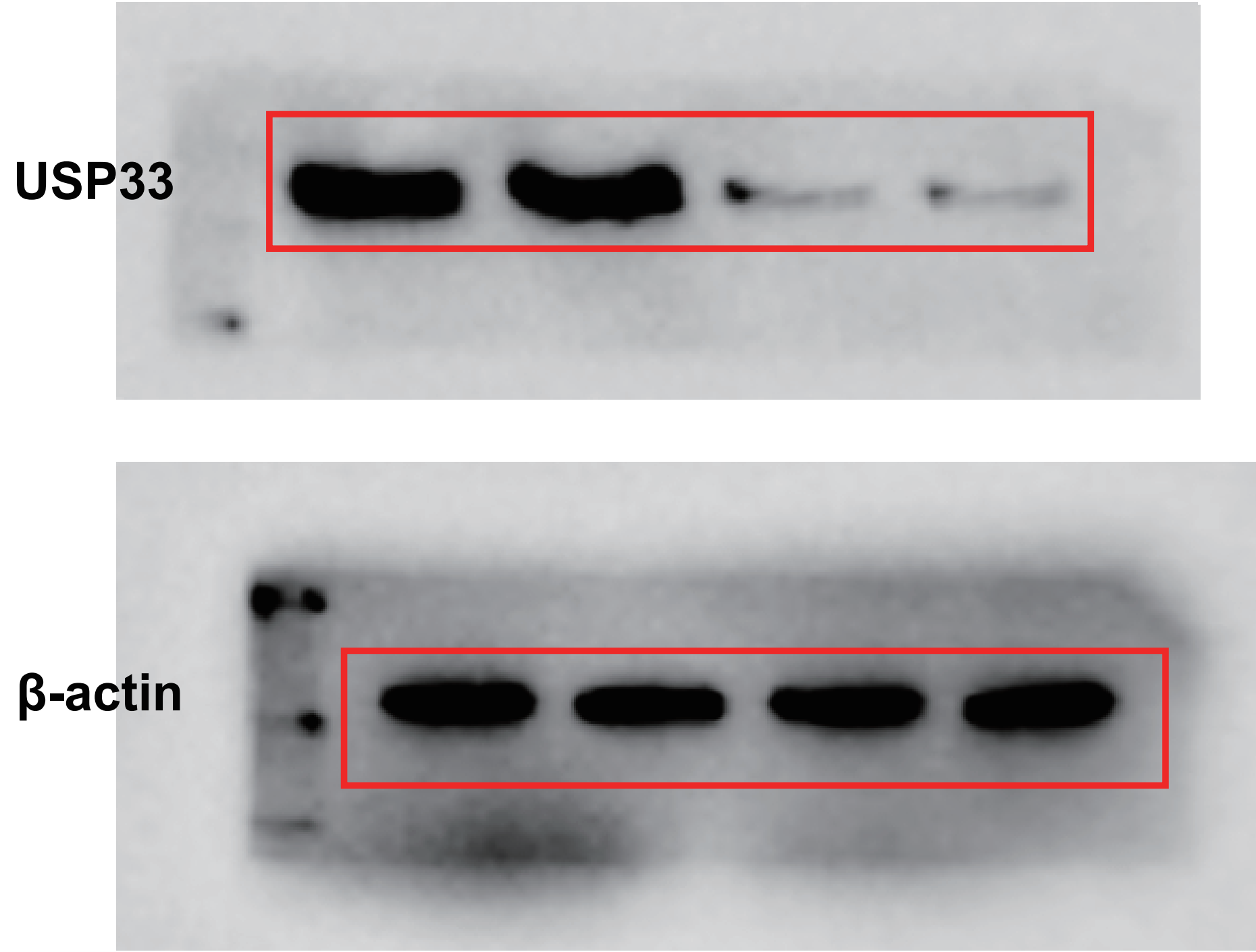

FigS1E

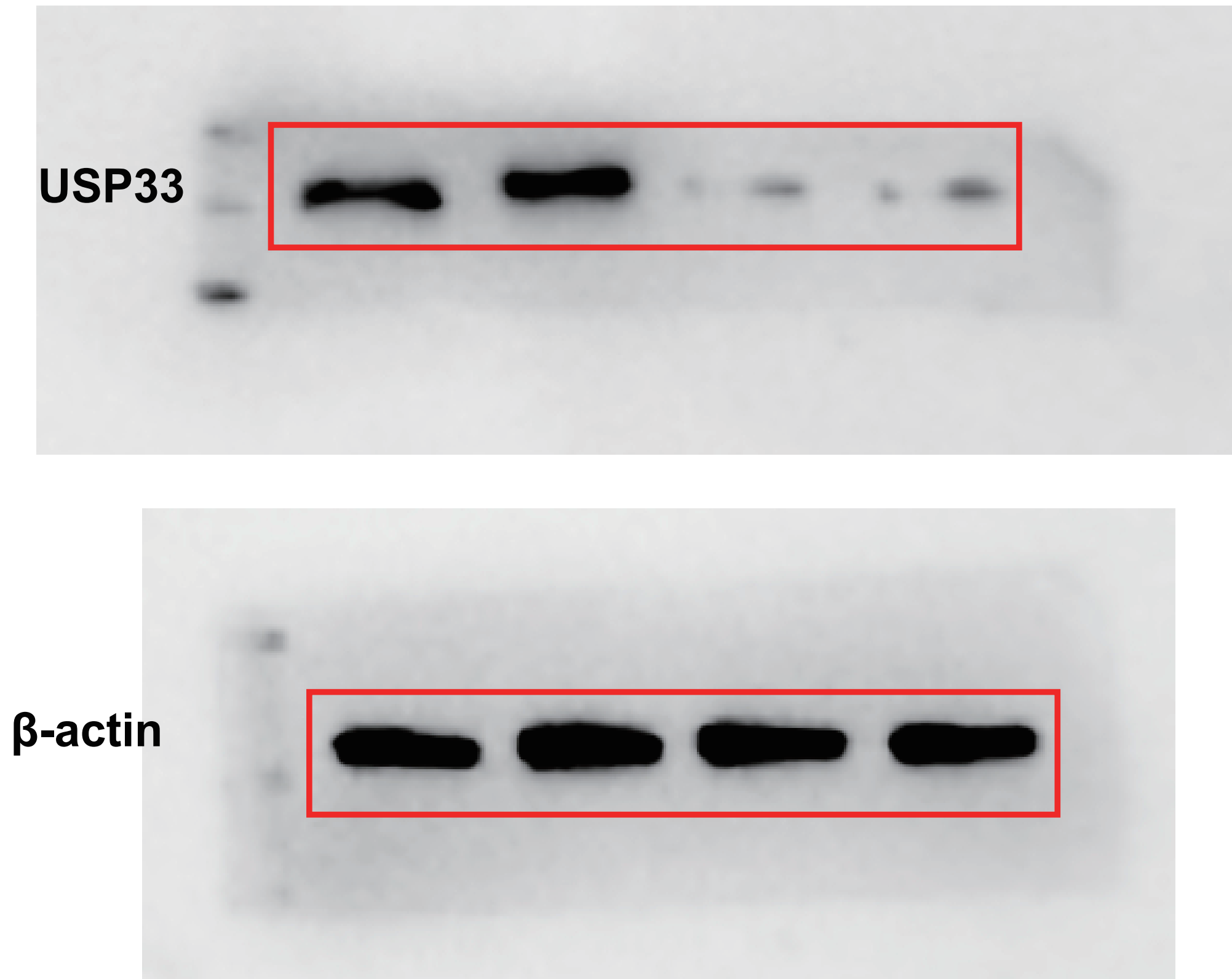

FigS3A

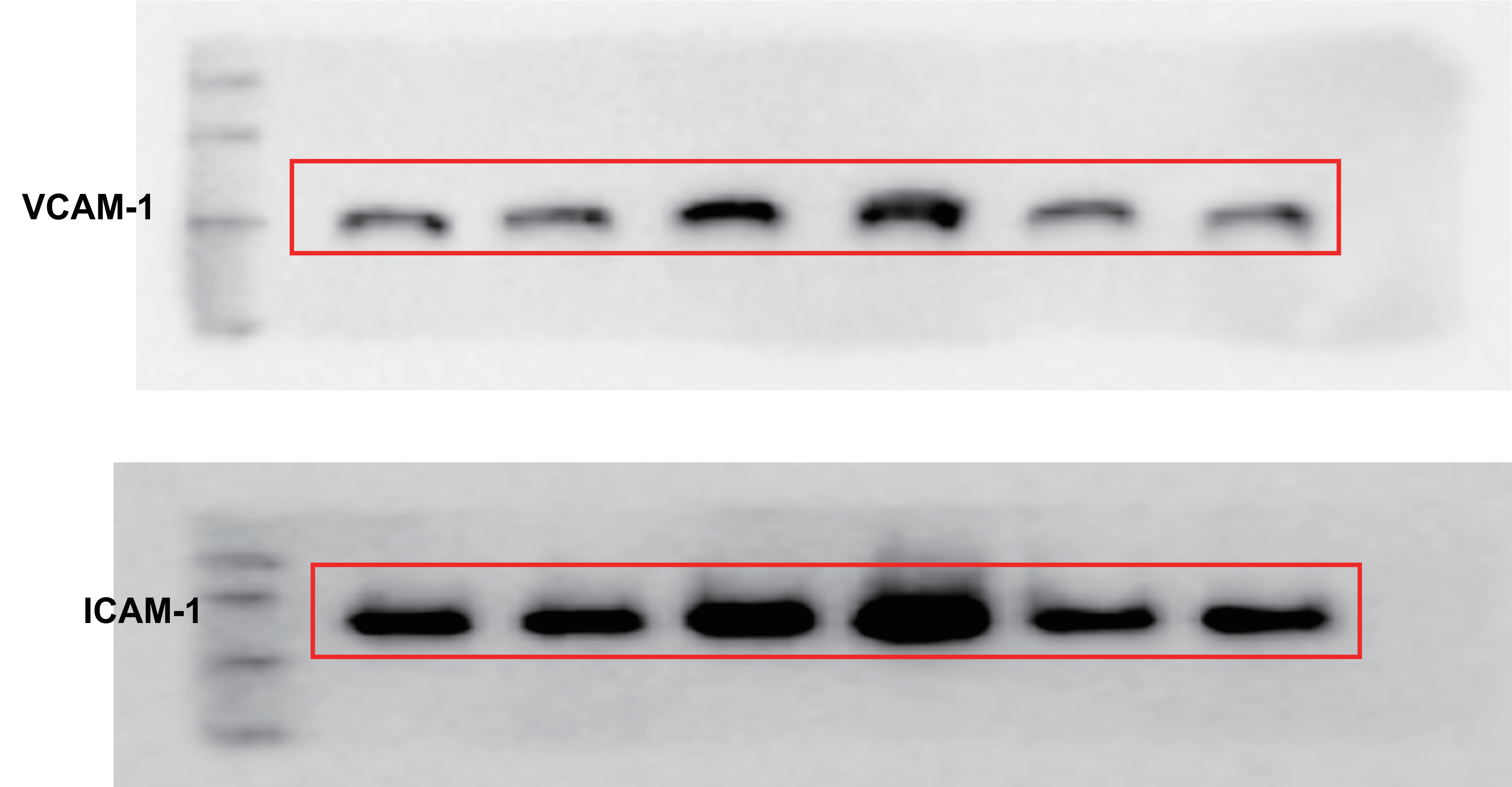

FigS3A

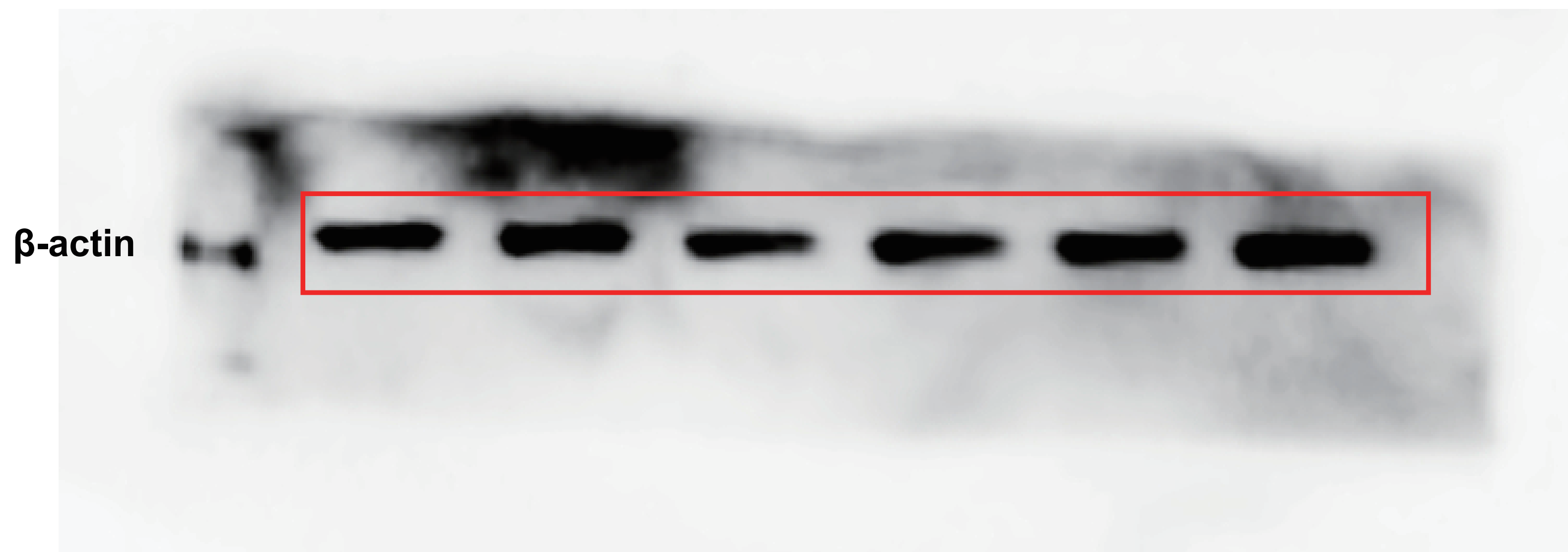

FigS3B

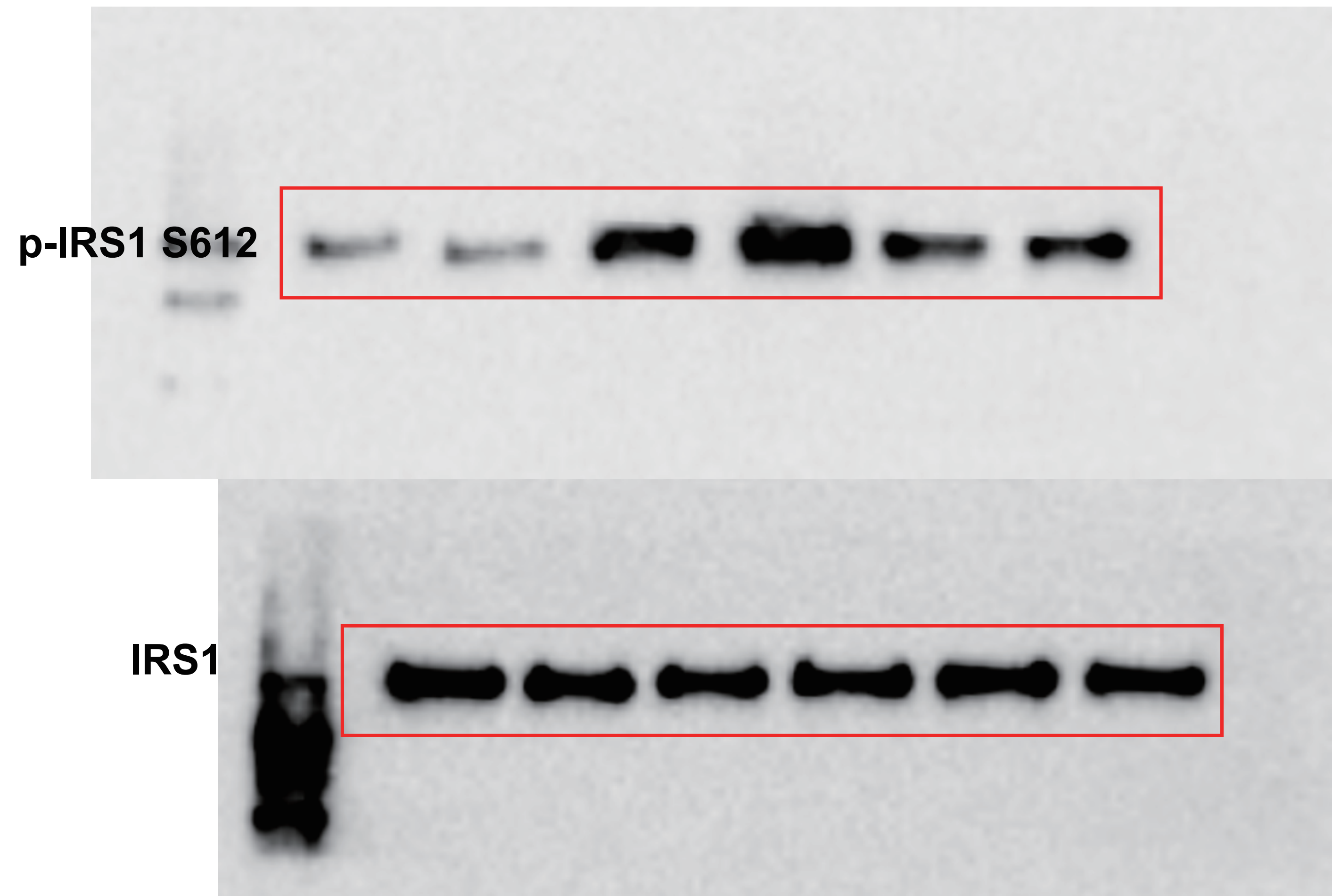

FigS3B

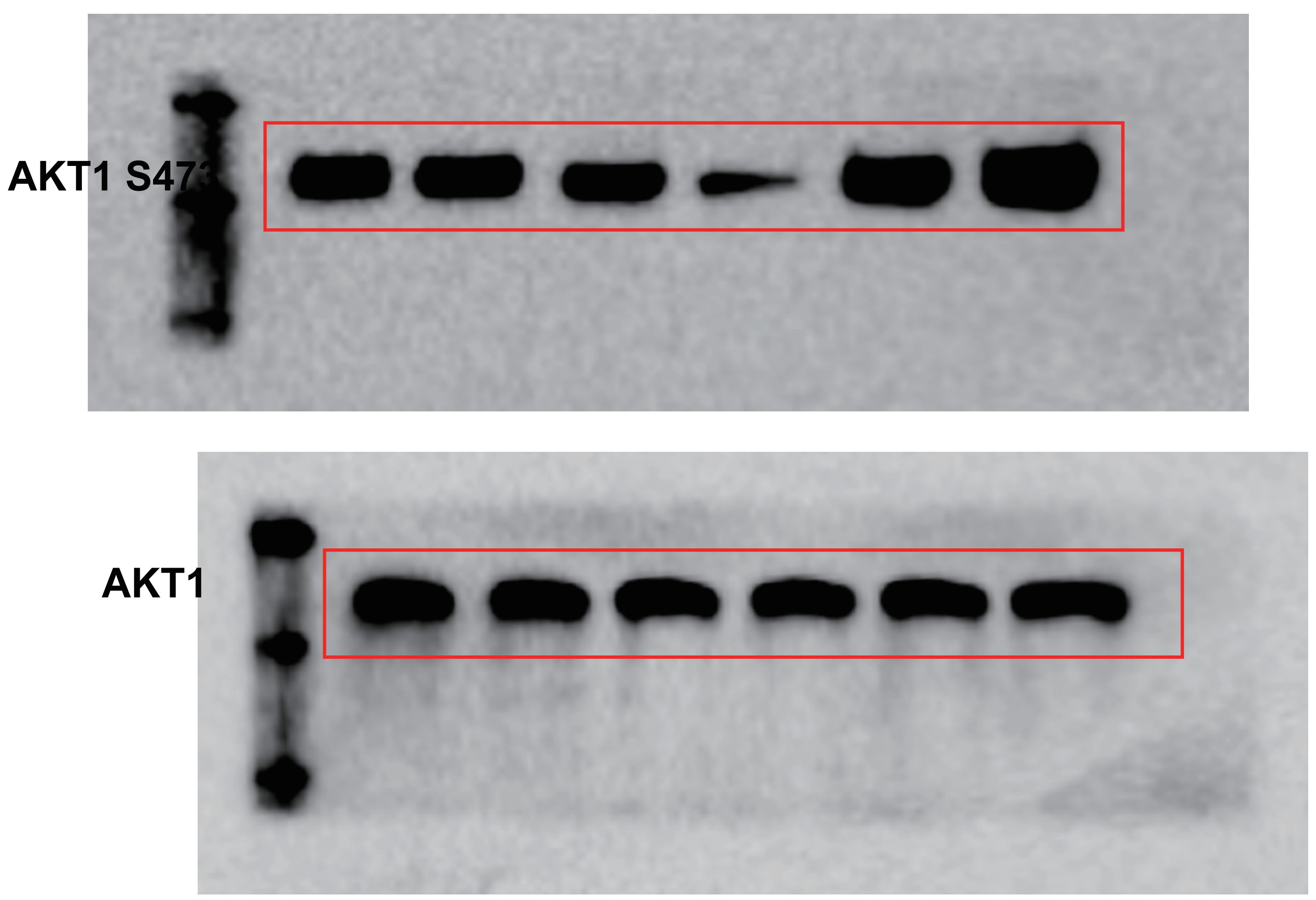

FigS3B

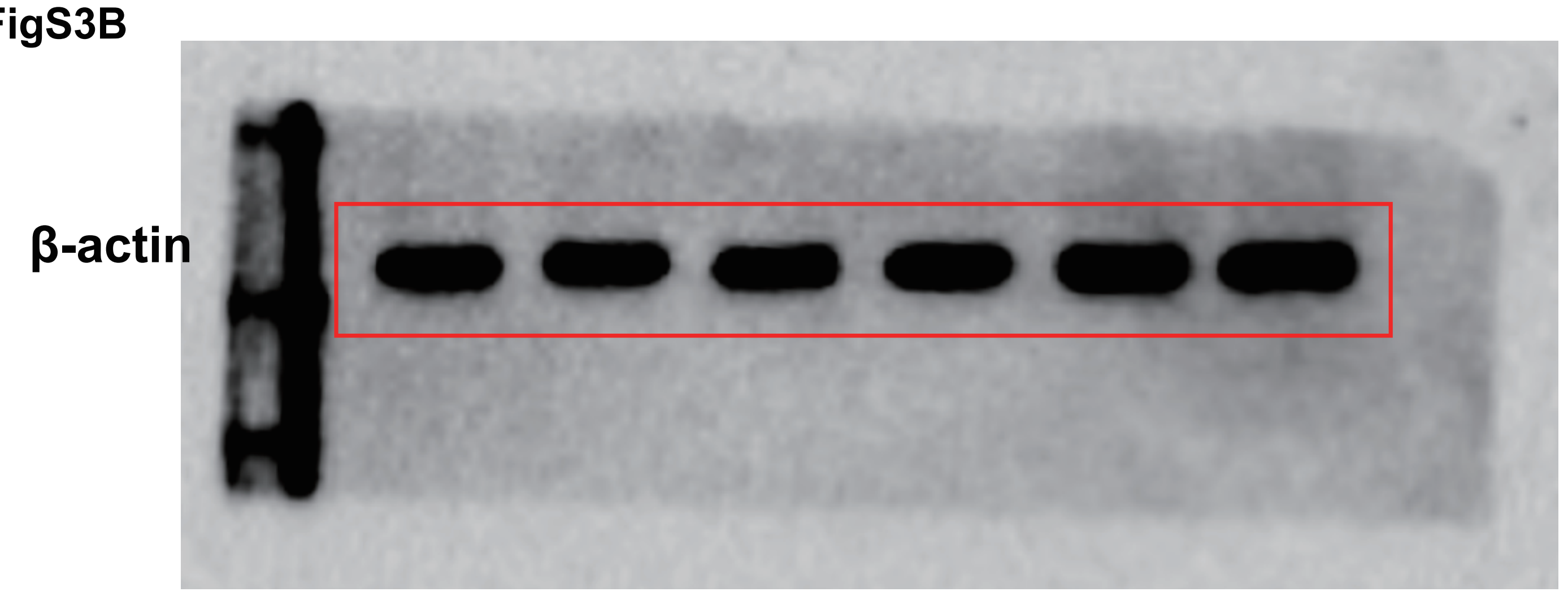

FigS4C

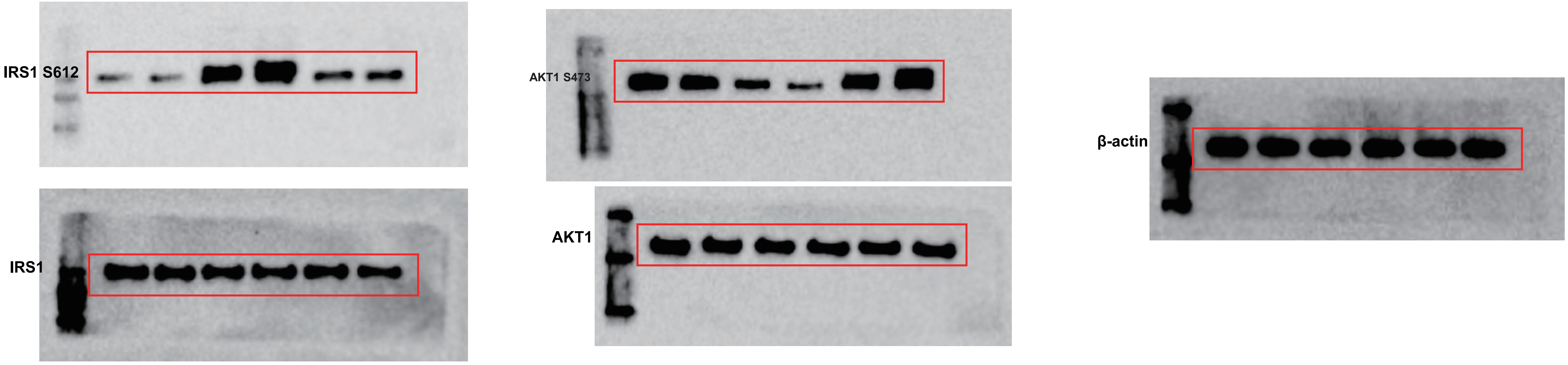

FigS4E

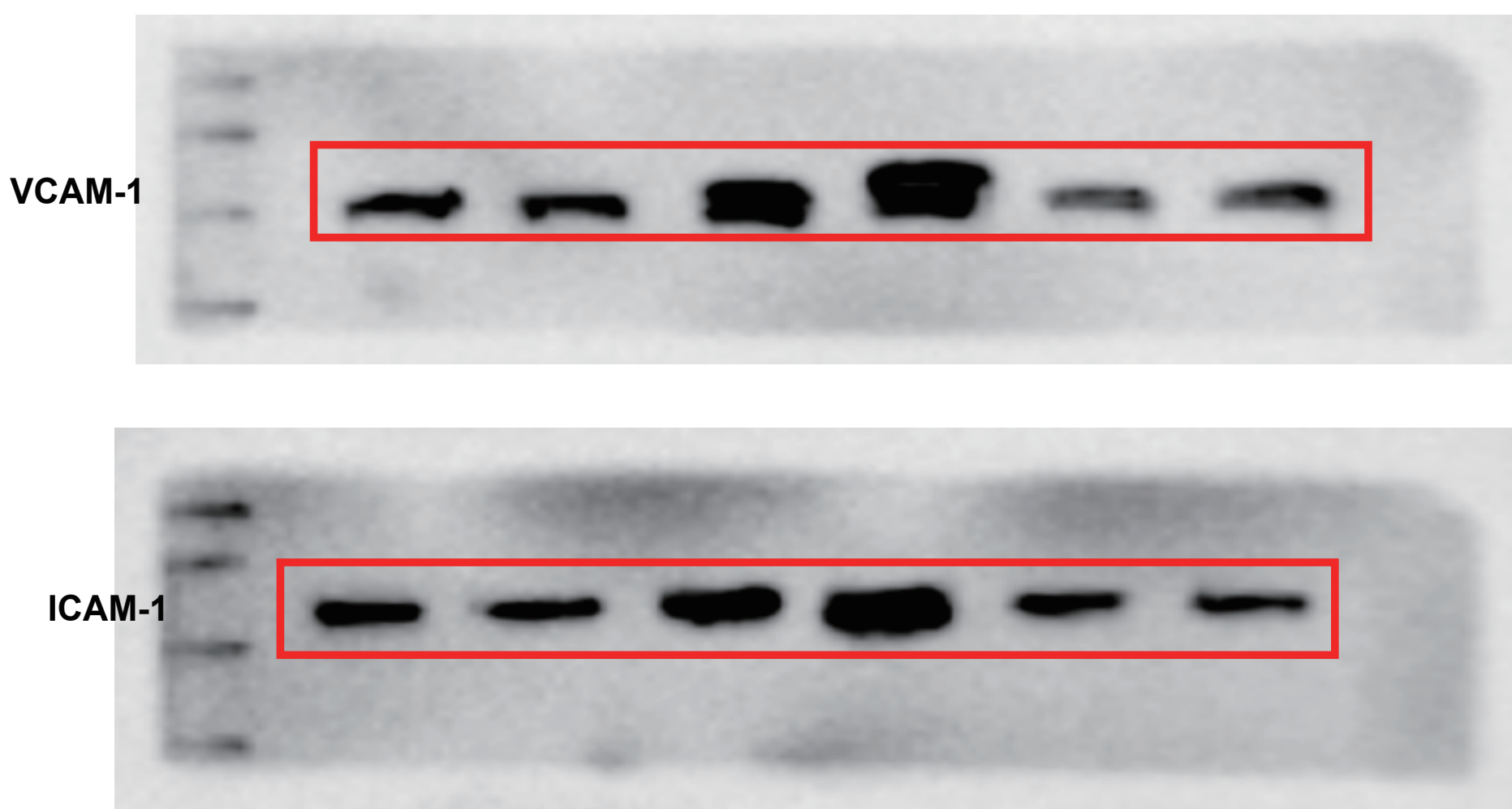

FigS4E

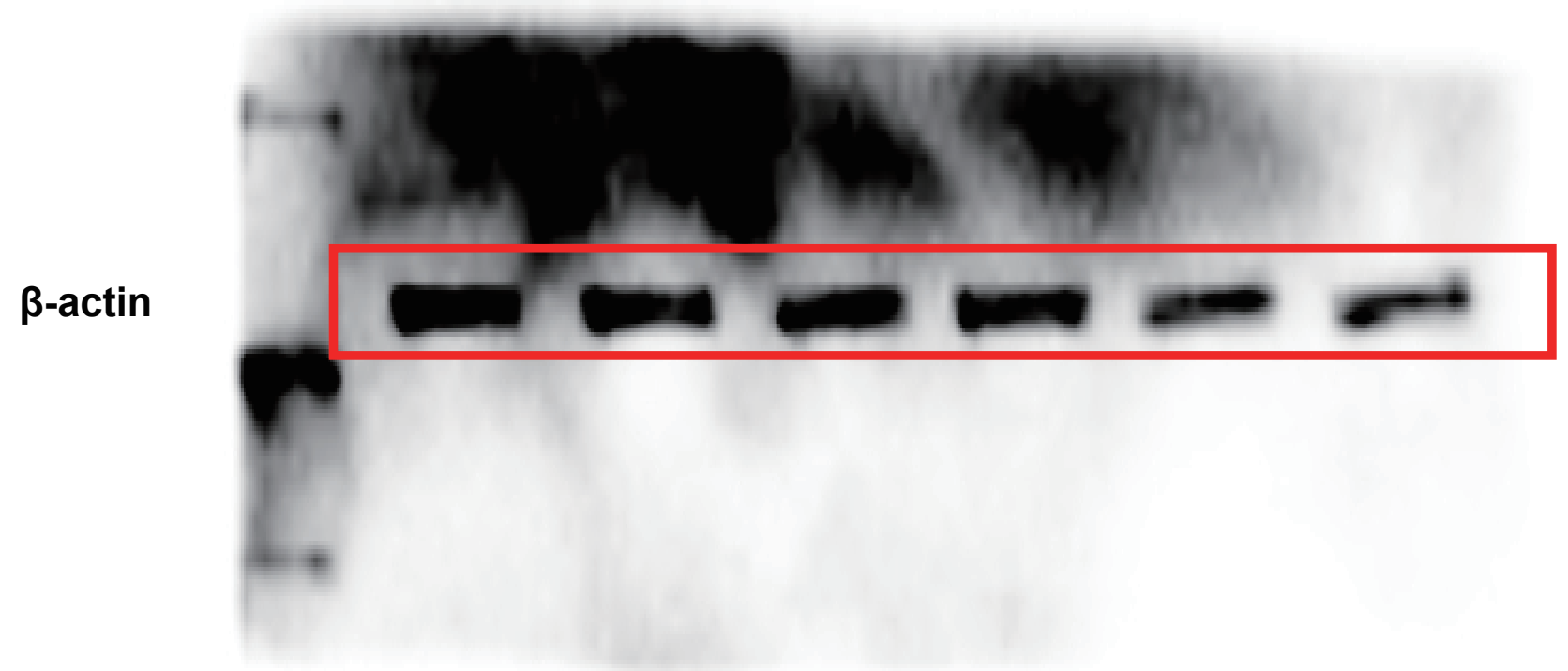

FigS5A

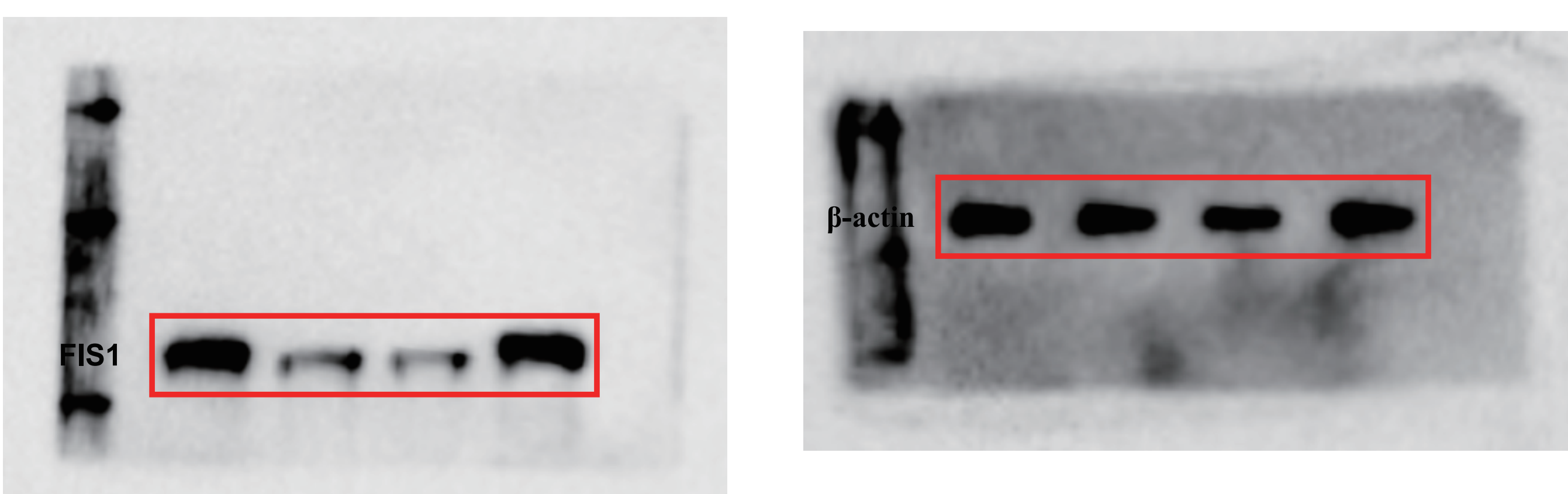

FigS5B

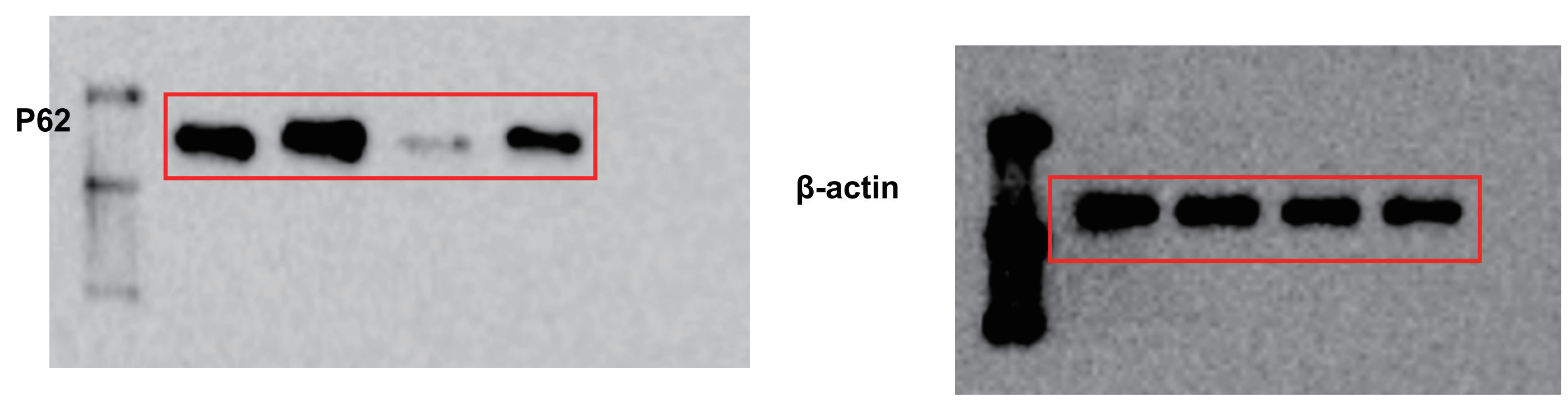

FigS6A

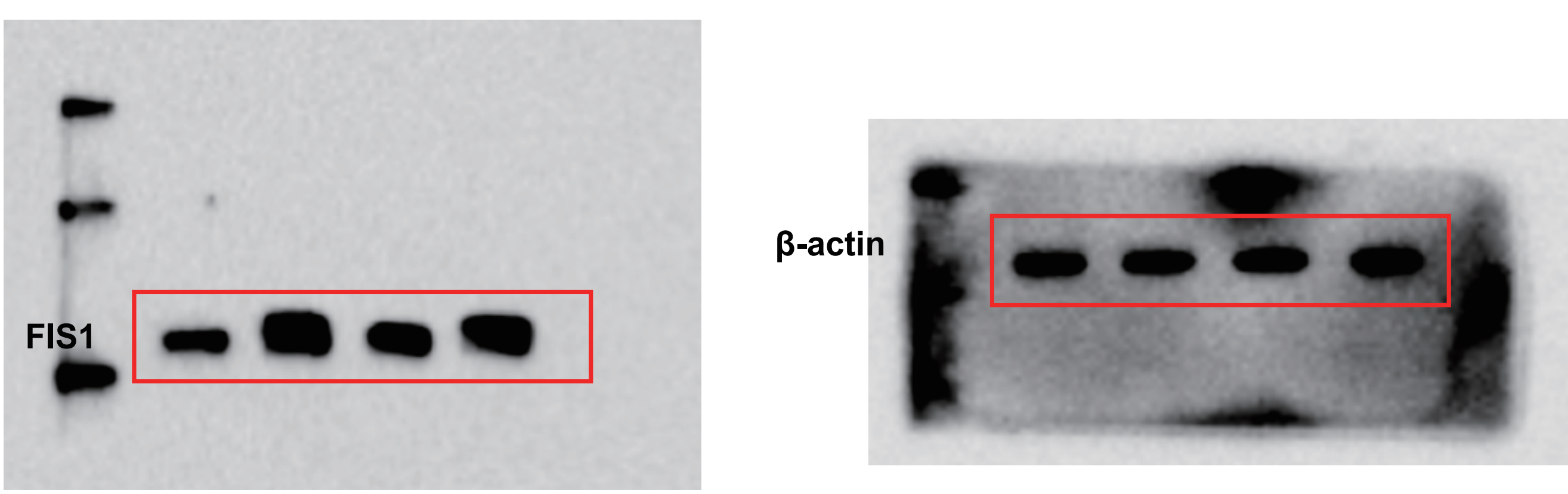

FigS6C

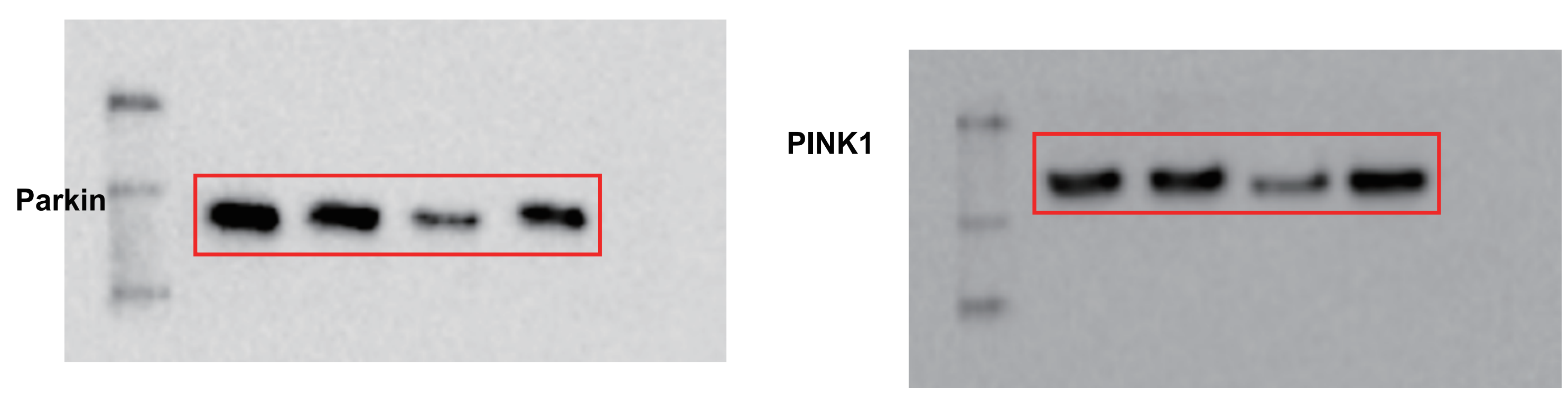

FigS6C

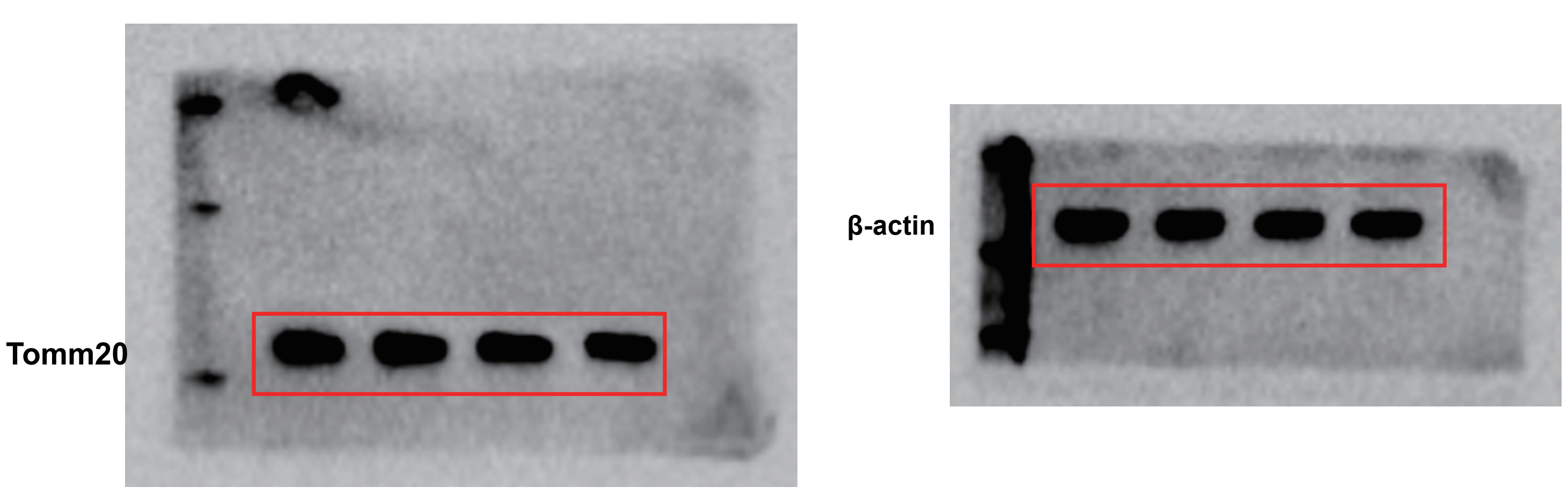

FigS6E

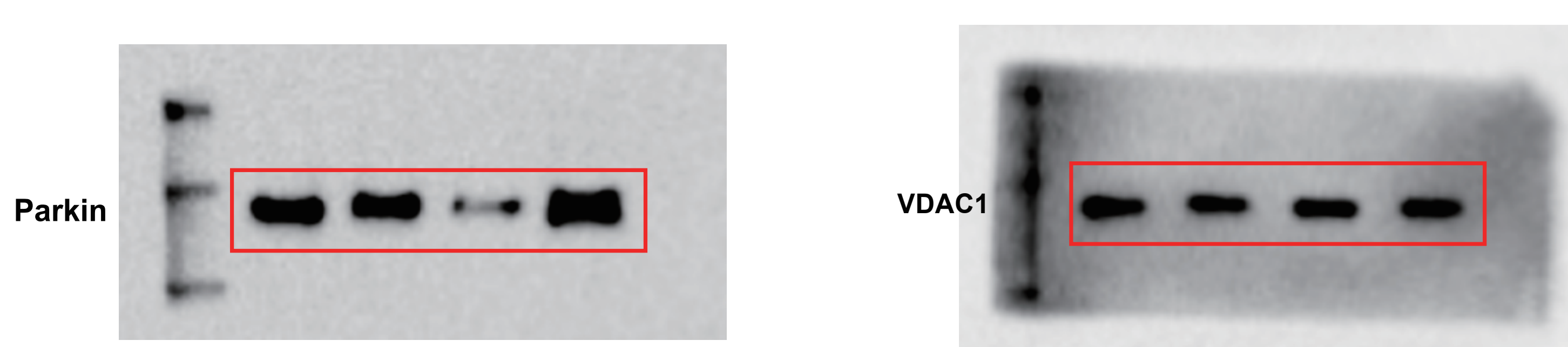

Figs7A

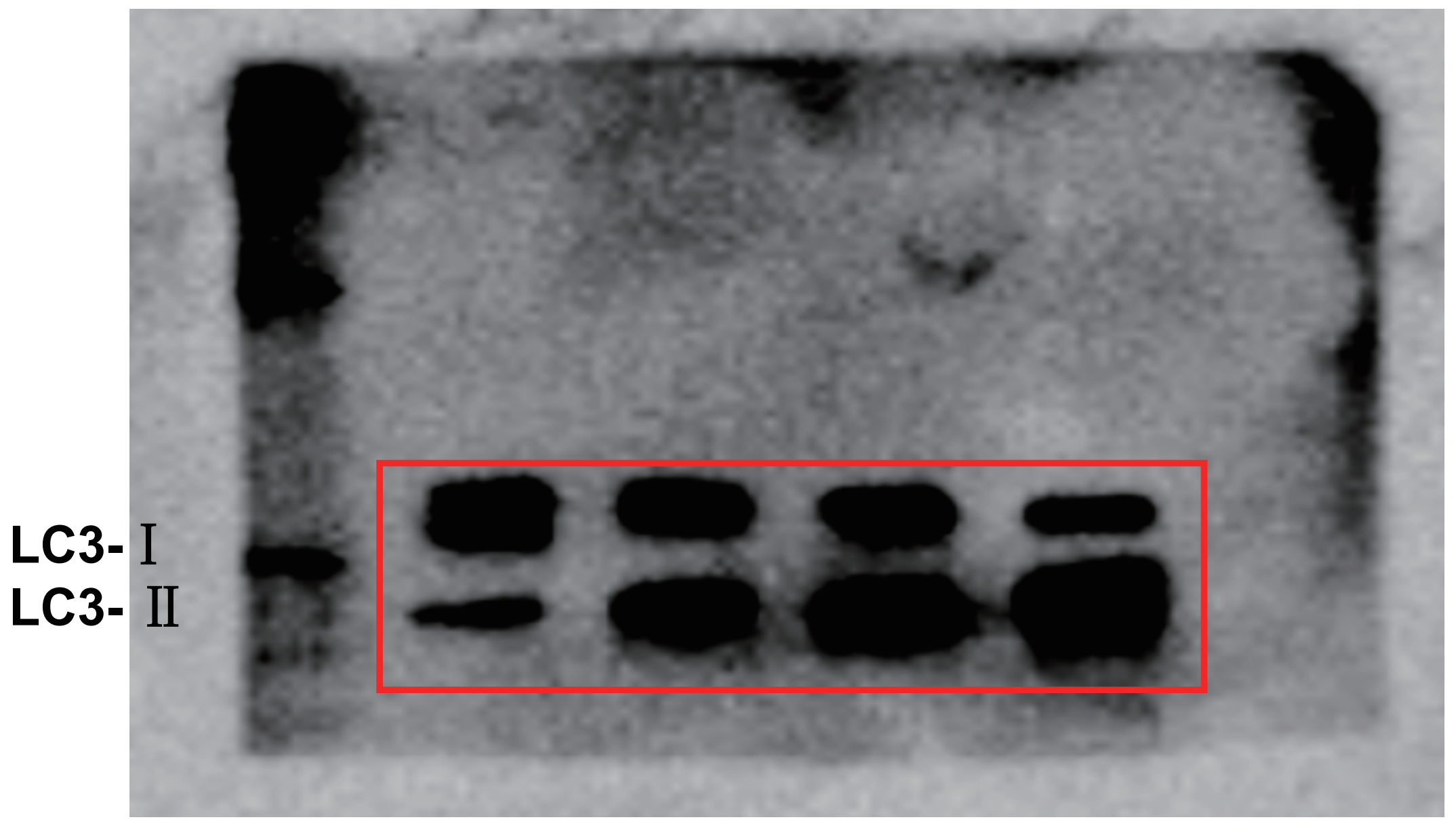

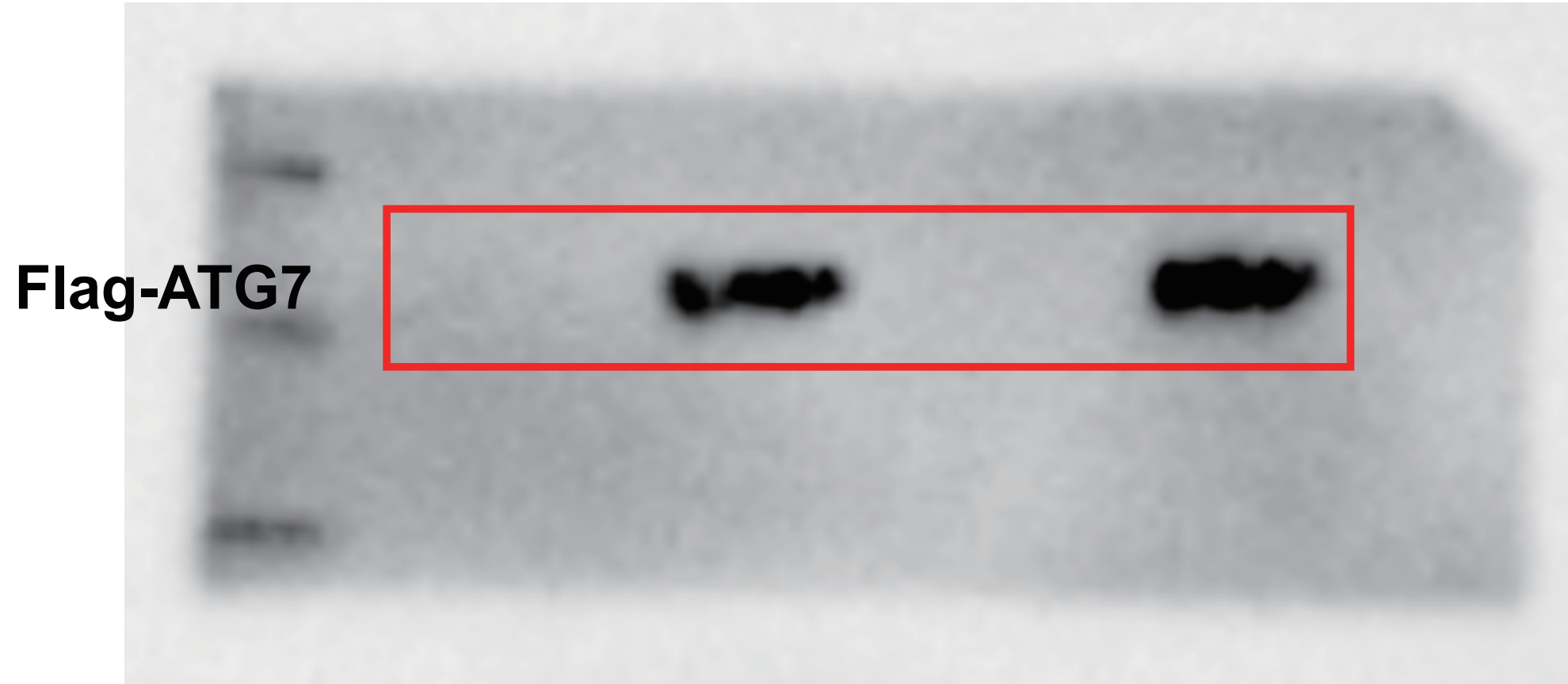

FigS7A

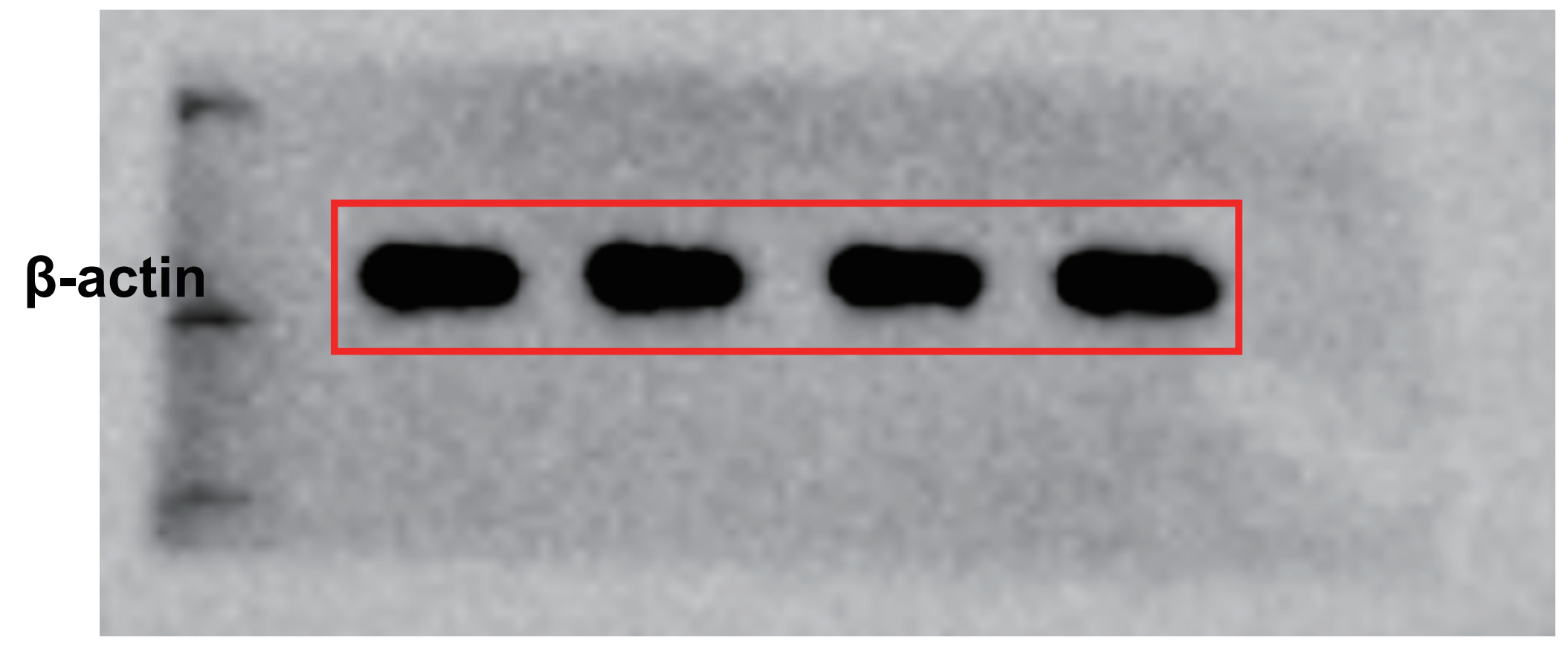

FigS7C

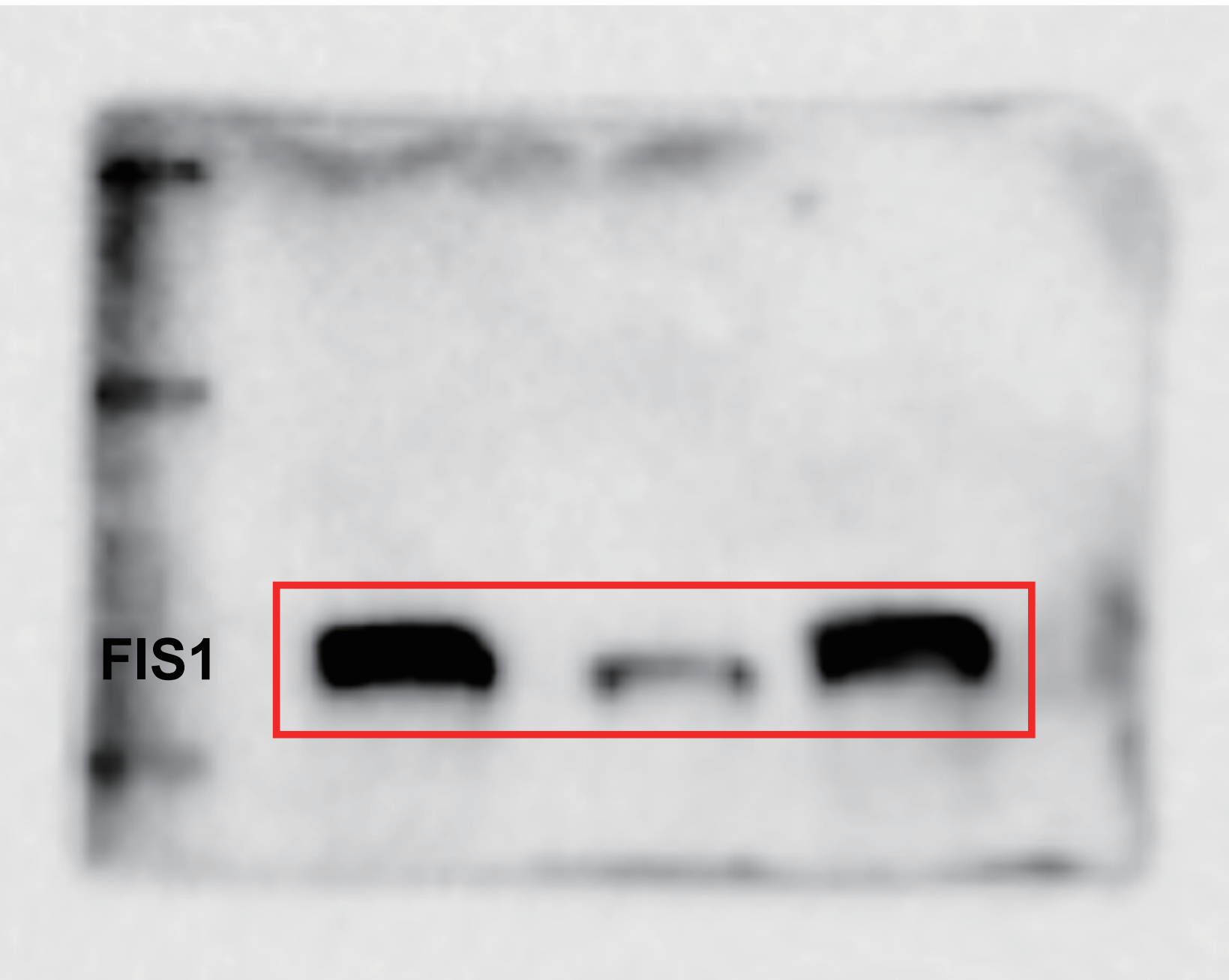

FigS7C

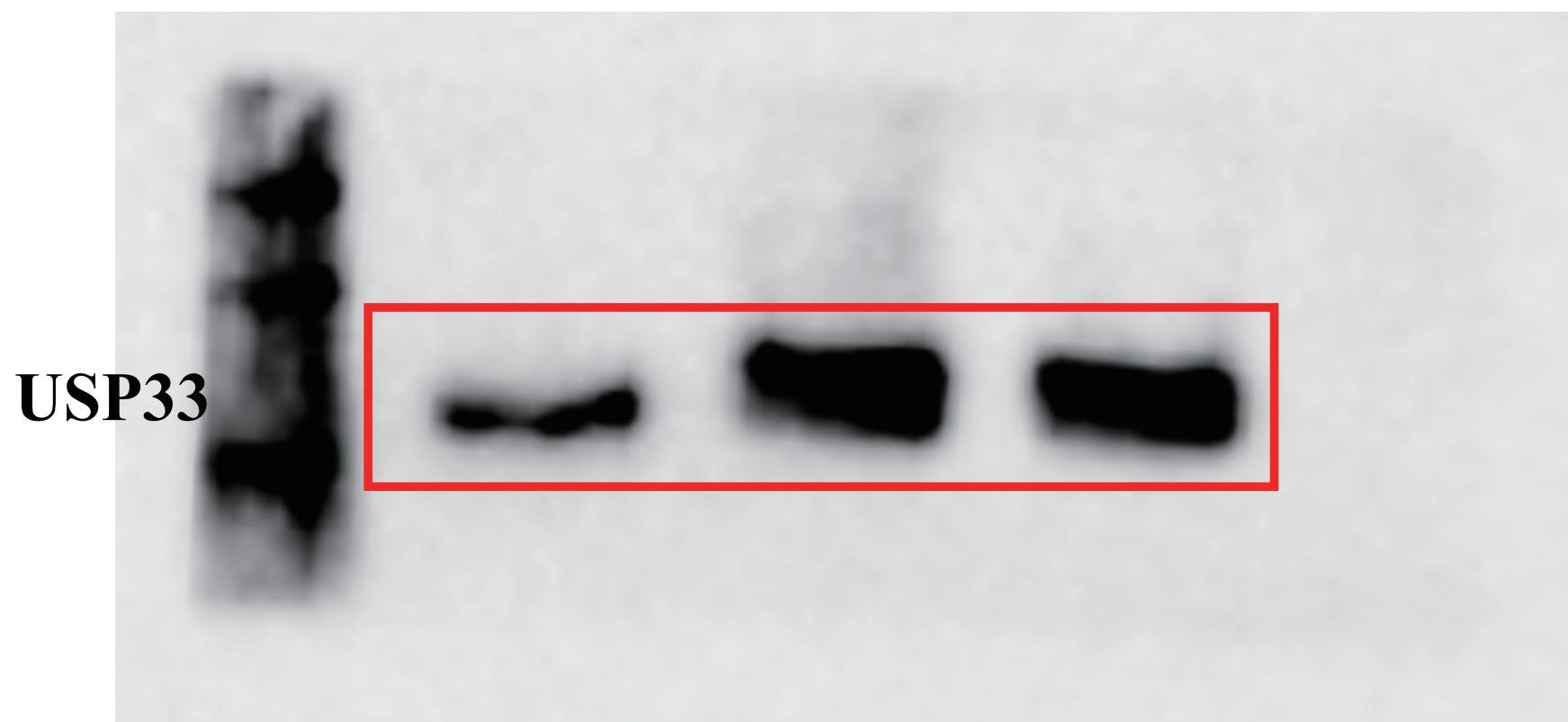

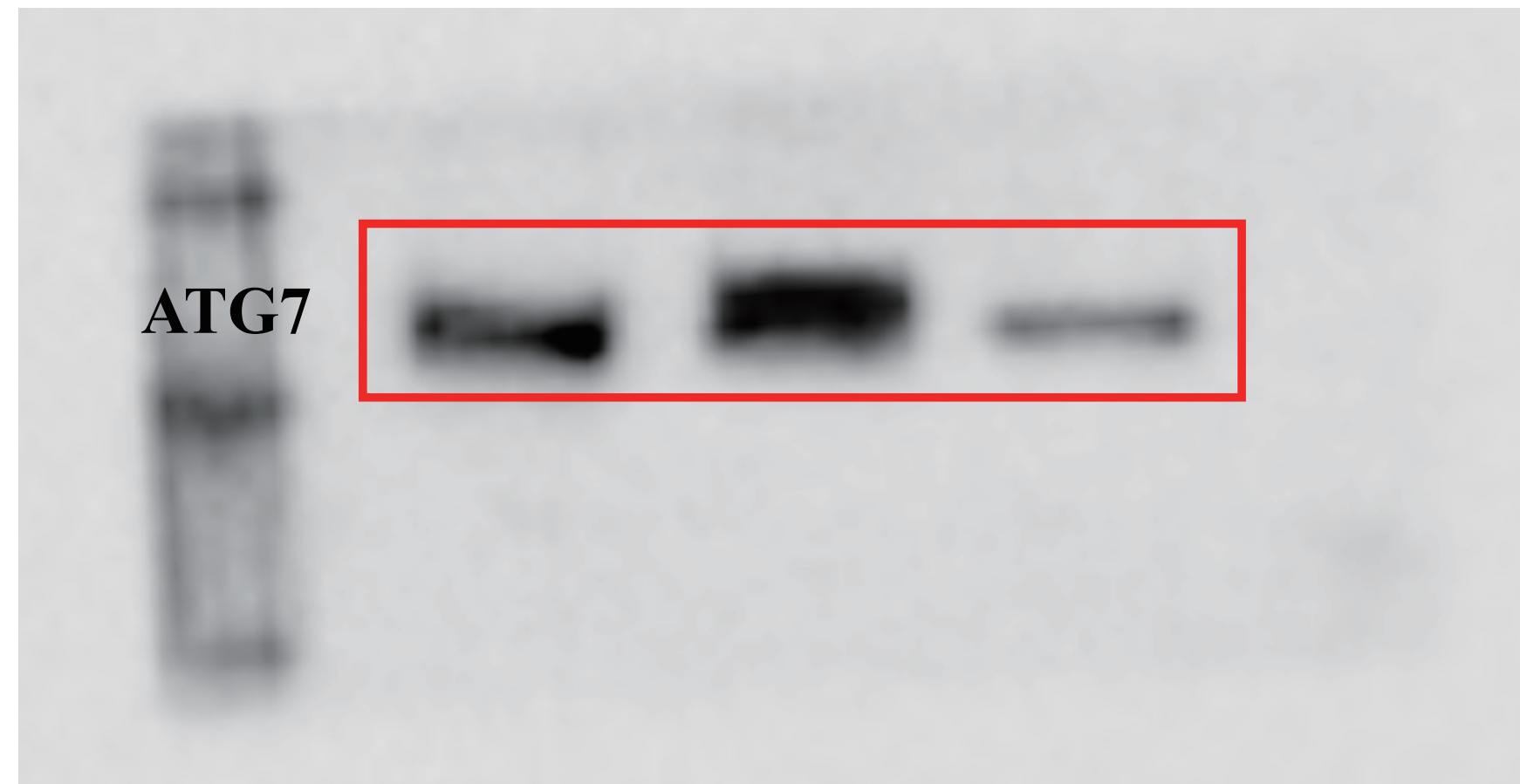

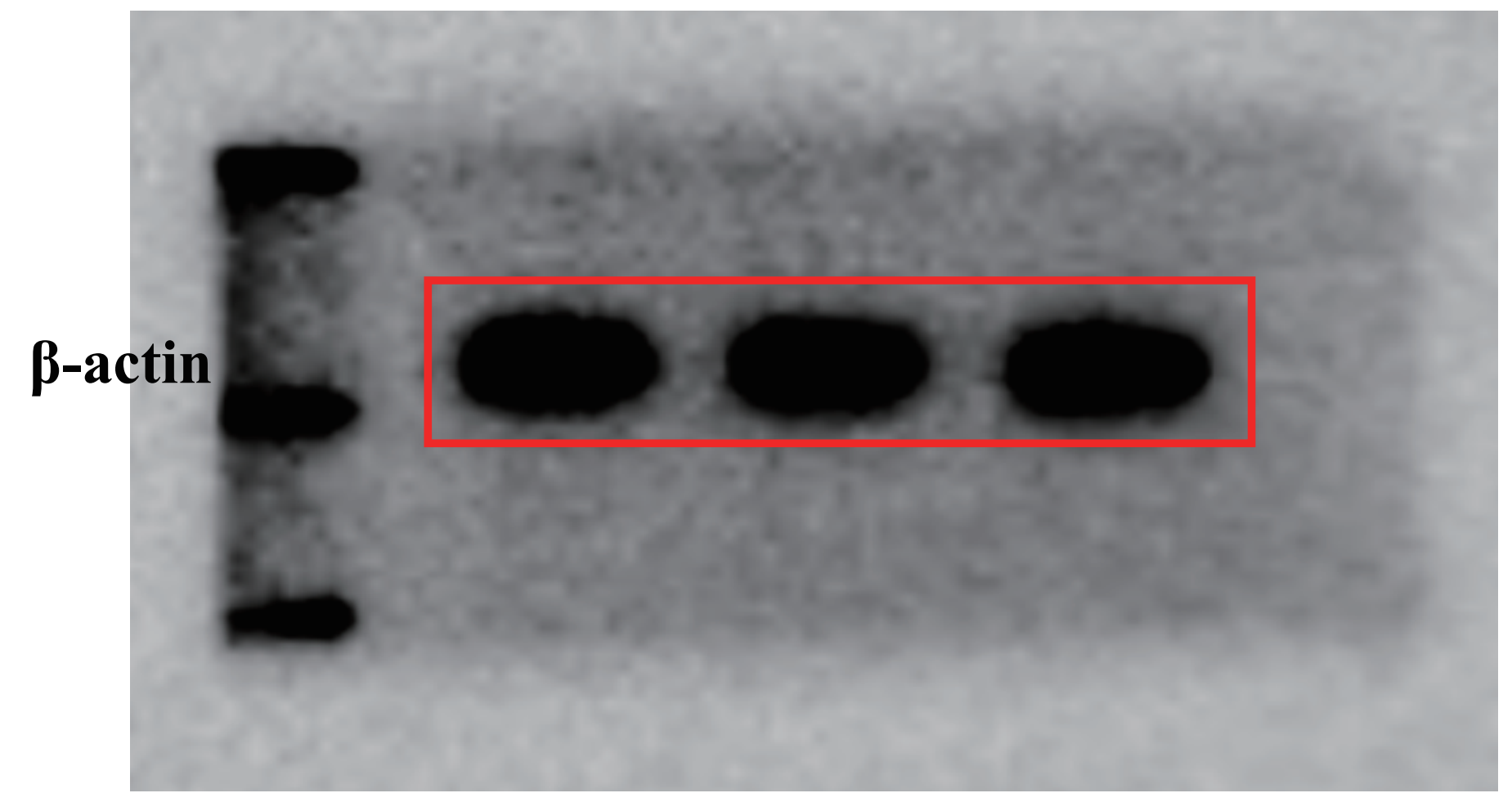

FigS7D

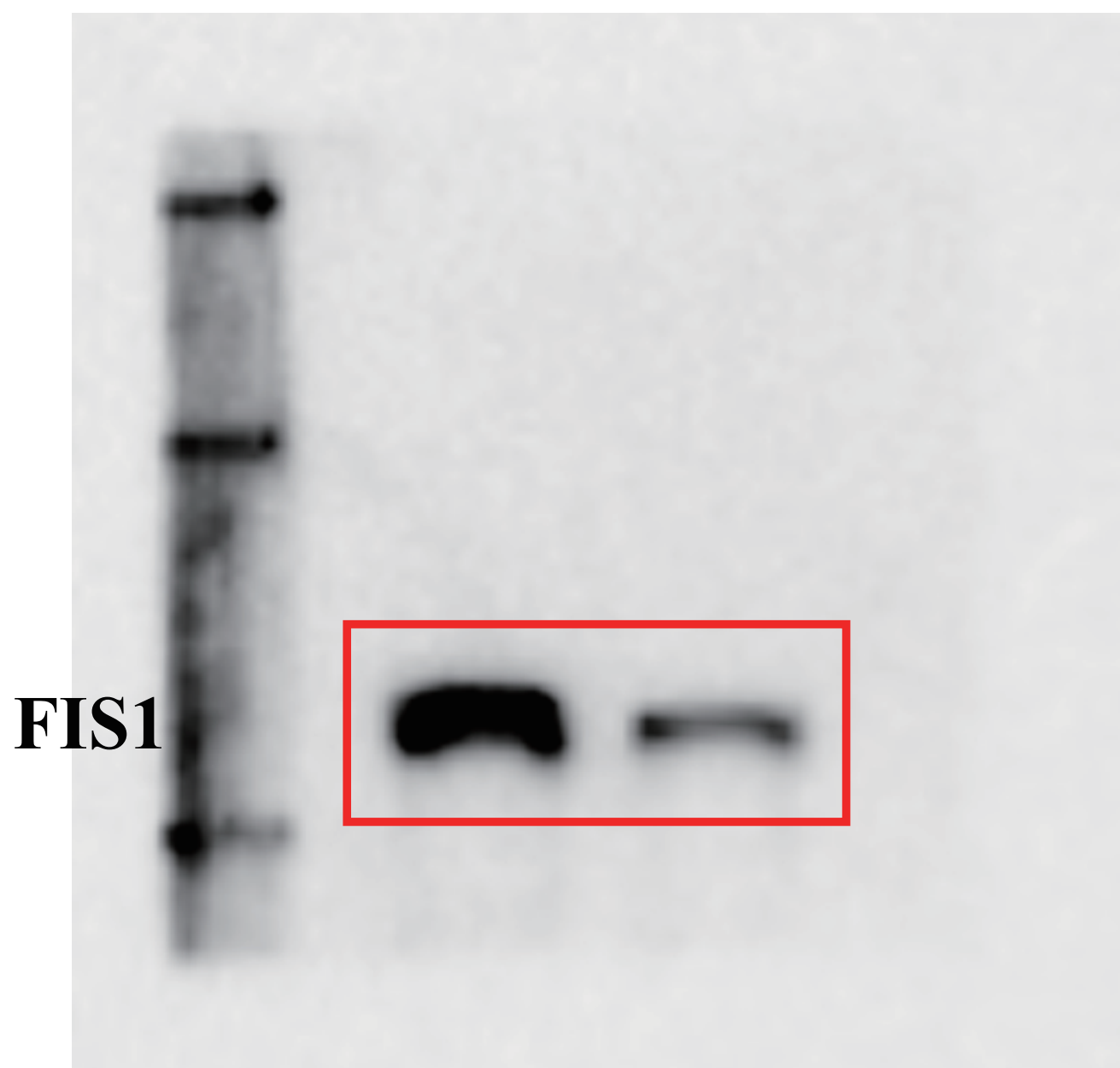

FigS7D

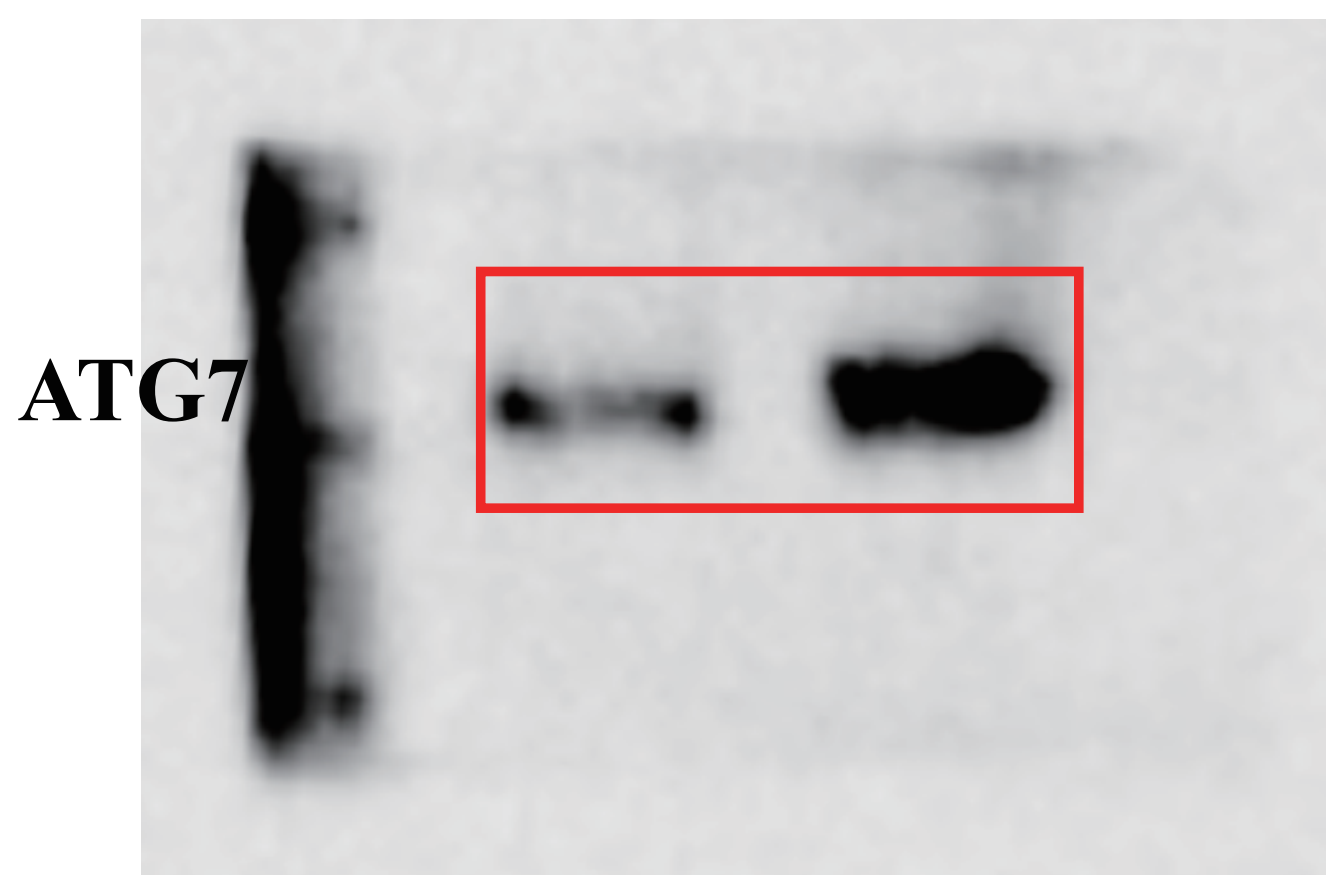

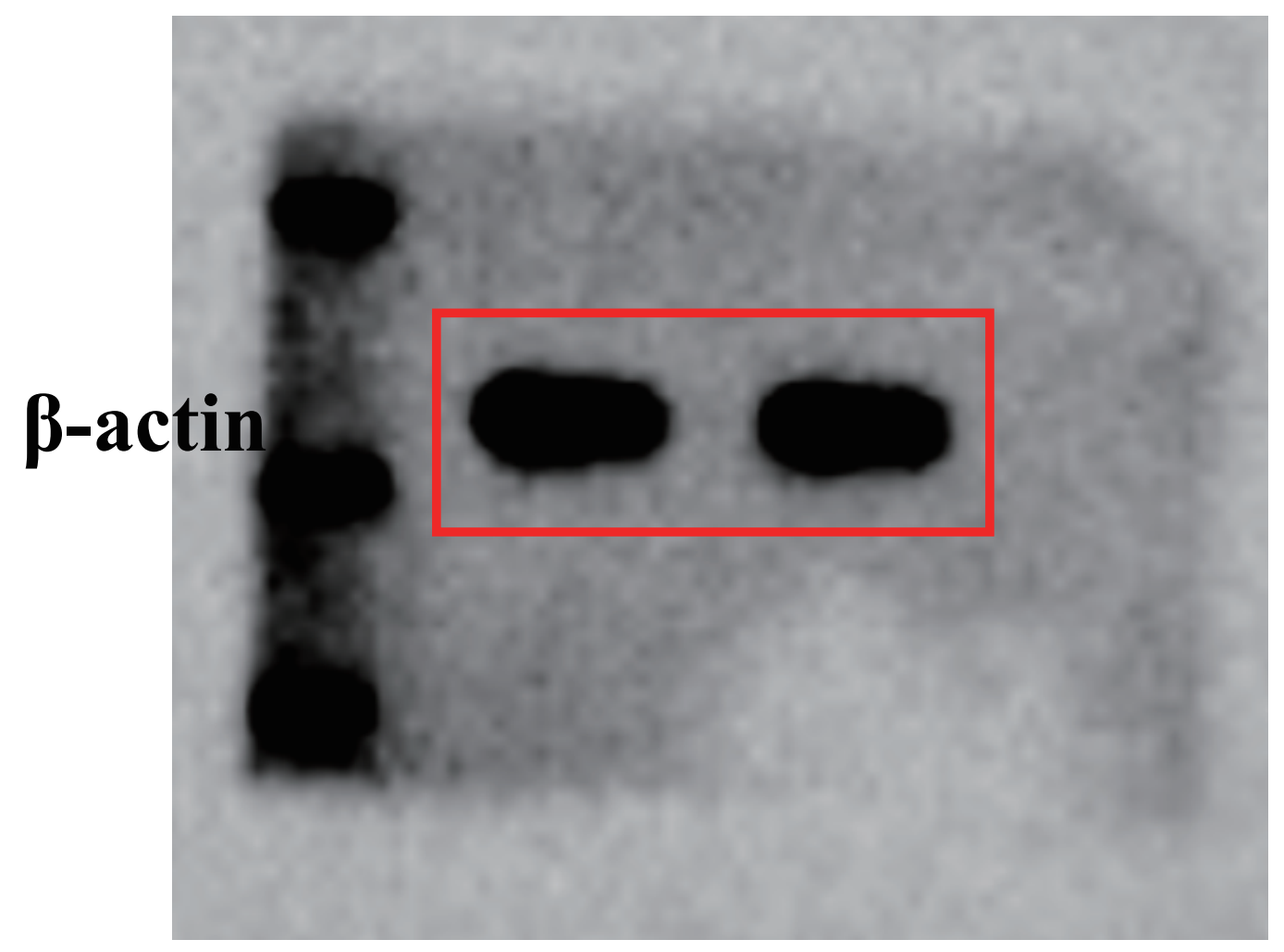

Supplement: Supplementary file 2 [file 41419_2026_8930_MOESM2_ESM.pdf]
